# Reduced APPL1 impairs osteogenic differentiation of mesenchymal stem cells by facilitating MGP expression to disrupt the BMP2 pathway in osteoporosis

**DOI:** 10.1016/j.jbc.2023.104823

**Published:** 2023-05-13

**Authors:** Weiquan Yuan, Wenjie Liu, Yunhui Zhang, Xinglang Wang, Chenhao Xu, Quanfeng Li, Pengfei Ji, Jiaxin Wang, Pei Feng, Yanfeng Wu, Huiyong Shen, Peng Wang

**Affiliations:** 1 Department of Orthopedics, The Eighth Affiliated Hospital, Sun Yat-sen University, Shenzhen, China; 2 Center for Biotherapy, The Eighth Affiliated Hospital, Sun Yat-sen University, Shenzhen, China

**Keywords:** APPL1, MSCs, MGP, osteogenic differentiation, osteoporosis

## Abstract

An imbalance of human mesenchymal stem cells (MSCs) adipogenic and osteogenic differentiation plays an important role in the pathogenesis of osteoporosis. Our previous study verified that Adaptor protein, phosphotyrosine interacting with PH domain and leucine zipper 1 (APPL1)/myoferlin deficiency promotes adipogenic differentiation of MSCs by blocking autophagic flux in osteoporosis. However, the function of APPL1 in the osteogenic differentiation of MSCs remains unclear. This study aimed to investigate the role of APPL1 in the osteogenic differentiation of MSCs in osteoporosis and the underlying regulatory mechanism. In this study, we demonstrated the downregulation of APPL1 expression in patients with osteoporosis and osteoporosis mice. The severity of clinical osteoporosis was negatively correlated with the expression of APPL1 in bone marrow MSCs. We found that APPL1 positively regulates the osteogenic differentiation of MSCs *in vitro* and *in vivo*. Moreover, RNA sequencing showed that the expression of MGP, an osteocalcin/matrix Gla family member, was significantly upregulated after APPL1 knockdown. Mechanistically, our study showed that reduced APPL1 impaired the osteogenic differentiation of mesenchymal stem cells by facilitating Matrix Gla protein expression to disrupt the BMP2 pathway in osteoporosis. We also evaluated the significance of APPL1 in promoting osteogenesis in a mouse model of osteoporosis. These results suggest that APPL1 may be an important target for the diagnosis and treatment of osteoporosis.

Osteoporosis is a systemic skeletal disease with an imbalance in bone mass characterized by decreased bone mineral density (BMD) and skeletal fragility (1). More than 200 million people worldwide suffer from osteoporosis (2). As the elderly population increases, the prevalence of osteoporosis continues to rise. Fractures and other complications caused by osteoporosis are debilitating for patients and their families, can lead to premature death, and impose a serious financial burden on society (3). Maintaining bone mass balance depends on the mutual regulation of osteoblasts and osteoclasts (4). Bone marrow mesenchymal stem cells (MSCs) are nonhematopoietic stem cells that exist in the bone marrow and have various differentiation potentials. MSCs differentiate into osteoblasts, which create new bone during bone formation and growth and play a major role in maintaining bone mass (5). However, the molecular mechanisms regulating the osteogenic differentiation of MSCs remain largely unknown, hindering the further application of MSCs in clinical treatment. Therefore, accelerating the differentiation of MSCs into osteoblasts and understanding the molecular mechanism regulating the osteogenic differentiation of MSCs are of great significance for the clinical treatment of osteoporosis.

Adaptor protein, phosphotyrosine interacting with PH domain and leucine zipper 1 (APPL1) is a ligand that binds directly to the intracellular regions of the adiponectin (APN) receptor (AdipoR) and plays a central role in APN and insulin signaling (6, 7). APPL1 interacts with AdipoR and mediates p38-mitogen-activated protein kinase (MAPK) and AMP-activated protein kinase activation, thereby regulating APN signaling and downstream events (8, 9). Our previous study verified that APPL1/myoferlin deficiency promotes adipogenic differentiation of MSCs by blocking autophagic flux in osteoporosis (10). Recent studies have shown that APPL1 is involved in regulating the osteogenic differentiation ability of MSCs. Knockdown of APPL1 downregulated the expression of osteogenic genes and calcium nodes induced by APN (11, 12). However, the molecular mechanism by which APPL1 regulates the osteogenic differentiation of MSCs has not been fully elucidated.

Matrix Gla protein (MGP), a member of the osteocalcin/matrix Gla family, has been reported as a physiological inhibitor of ectopic tissue calcification (13). Studies have confirmed that MGP inhibits arterial, synovial, and cartilage calcification and genetic deficiencies of MGP in mice and humans have been linked to abnormal mineralization of soft tissues (14, 15, 16). In addition, previous studies have shown that MGP is associated with osteoarthritis. Studies have reported a significant association between hand osteoarthritis and genetic variants in MGP (17, 18, 19). Despite these findings, the mechanisms involved in the effects of MGP on maintaining regeneration and osteogenic differentiation of MSCs remain unclear.

Transforming growth factor-beta (TGF-β) and bone morphogenetic protein (BMP) signaling play fundamental roles in embryonic skeletal development and bone homeostasis (20, 21). TGF-βs and BMPs transduce signals to both the canonical and the noncanonical Smad-independent signaling pathways and activate downstream transcription factors, such as RUNX family transcription factor 2 (Runx2), to enhance osteogenic and chondrogenic differentiation of MSCs (20, 22, 23, 24). Dysregulation of these signals can lead to a range of bone diseases in humans. Knockout or mutation of TGF-β and BMP signaling-related genes in mice resulted in different degrees of skeletal abnormalities (25, 26, 27). Moreover, it has been reported that MGP can stimulate vascular endothelial growth factor expression by increasing TGF-β activity in endothelial cells (28). MGP, a regulatory protein of BMP2, has been shown to bind BMP2 and interfere with BMP2 binding to its receptor and downstream Smad1 activation (29). However, whether MGP regulates the osteogenic differentiation of MSCs through the BMP2 pathway remains unexplored.

In this study, we detected the levels of APPL1 in patients with osteoporosis and osteoporosis mice and analyzed the gene expression profile of osteogenic differentiation of human bone marrow MSCs with APPL1 knockdown, aiming to explore the mechanism of APPL1 in osteogenic differentiation of MSCs in osteoporosis. We also evaluated the significance of APPL1 in promoting osteogenesis in a mouse model of osteoporosis.

## Results

### APPL1 expression is decreased in osteoporosis

**Figure 1:**
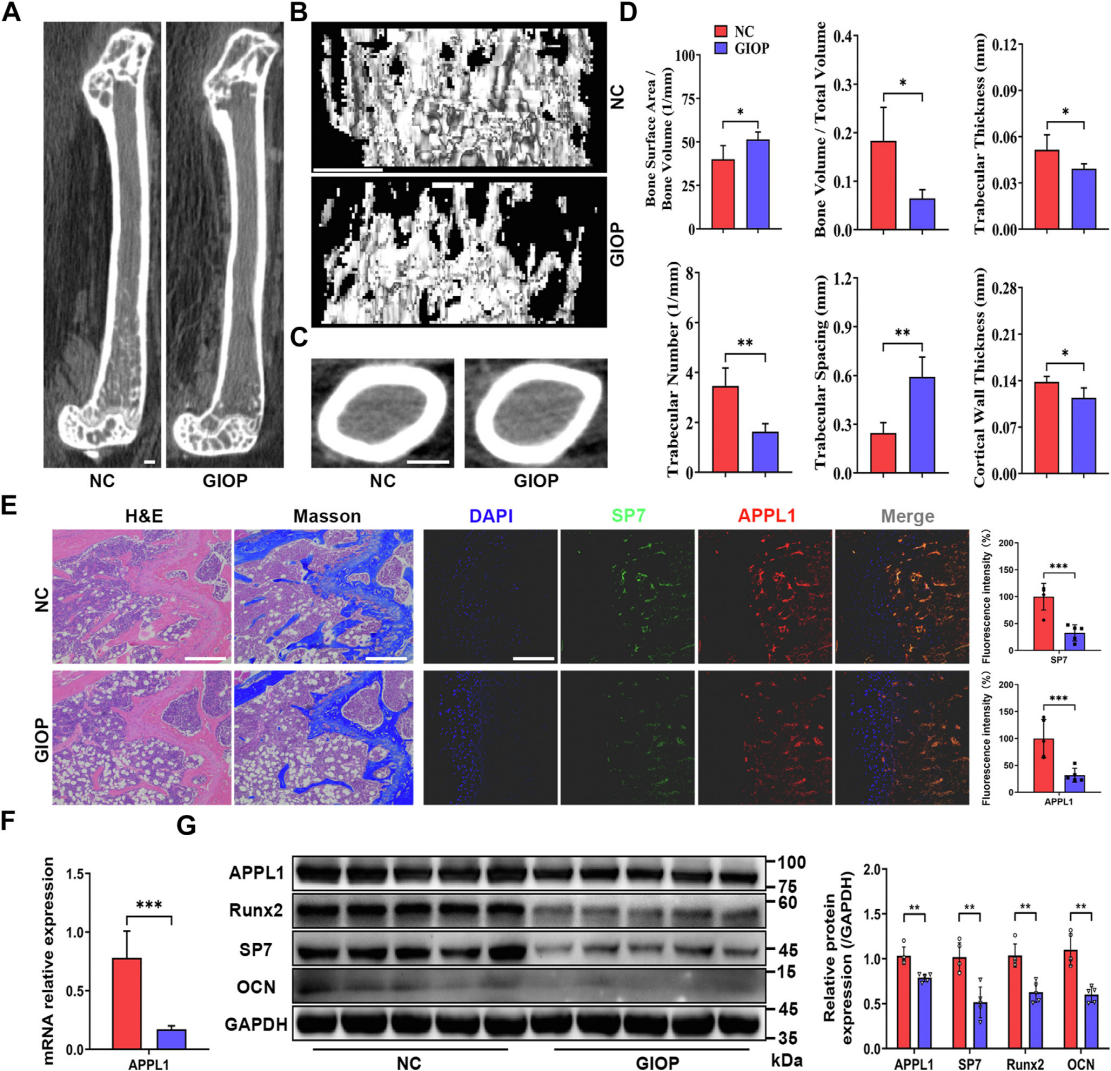
**APPL1 expression is decreased in osteoporosis.***A*, sagittal view of the femur in the GIOP mouse model. *B*, representative micro-CT 3D reconstruction of trabecular bone in the proximal femur. *C*, midsection of the femur in the GIOP mouse model. *D*, measurements of BSA/BV, BV/TV, Tb.Th, Tb.N, and Tb.Sp in proximal femur trabecular bone and Ct.Th in the mid-femur after 8 weeks of intervention. *E*, *left*: H&E staining and Masson staining of bone tissue in NC and GIOP groups of mice, *right*: immunofluorescence staining and quantitative analyses of SP7 and APPL1 in bone tissue of NC and GIOP groups of mice. *F* and *G*, the mRNA and protein levels of APPL1 in the tibia of the negative control and GIOP groups were measured by qRT‒PCR and Western blotting (Data were normalized to GAPDH). The data are shown as the mean ± SD (n = 5 per group). ∗*p* < 0.05, ∗∗*p* < 0.01, ∗∗∗*p* < 0.001 compared with the NC group. Scale bar = 100 μm. APPL1, Adaptor protein, phosphotyrosine interacting with PH domain and leucine zipper 1; GIOP, glucocorticoid-induced osteoporosis; micro-CT, micro-computed tomography; NC, normal control.

Our previous study showed that the expression of APPL1 was downregulated in osteoporosis and that the impairment of APPL1/myoferlin facilitates adipogenic differentiation of MSCs by blocking autophagic flux (10). To further clarify the role of APPL1 in osteogenesis in osteoporosis, we successfully established a mouse model of glucocorticoid-induced osteoporosis (GIOP). The femurs were harvested and reconstructed by micro-CT, and trabecular and cortical parameters were evaluated. After prolonged and continuous administration of glucocorticoids, the microstructure of the mouse femur was disrupted in the GIOP group (Fig. 1, *A* and *B*). In addition, the trabecular bone morphological parameters (BV/TV, BSA/BV, Tb.Th, Tb.N, and Tb.Sp) used to evaluate bone strength were poor, and the cortical thickness also became thinner (Fig. 1, *C* and *D*). Moreover, H&E and Masson staining of bone tissue were consistent with the micro-CT scan results (Fig. 1*E*). Immunofluorescence staining and quantitative analyses showed that APPL1 and osteogenic markers SP7 expression was decreased in osteoporotic femurs (Fig. 1*E*). Subsequently, bone marrow MSCs were isolated from the tibia of mice, and qRT‒PCR and Western blotting showed that APPL1 was significantly decreased in GIOP mice (Fig. 1, *F* and *G*).

### APPL1 is upregulated during the osteogenic differentiation of MSCs

**Figure 2:**
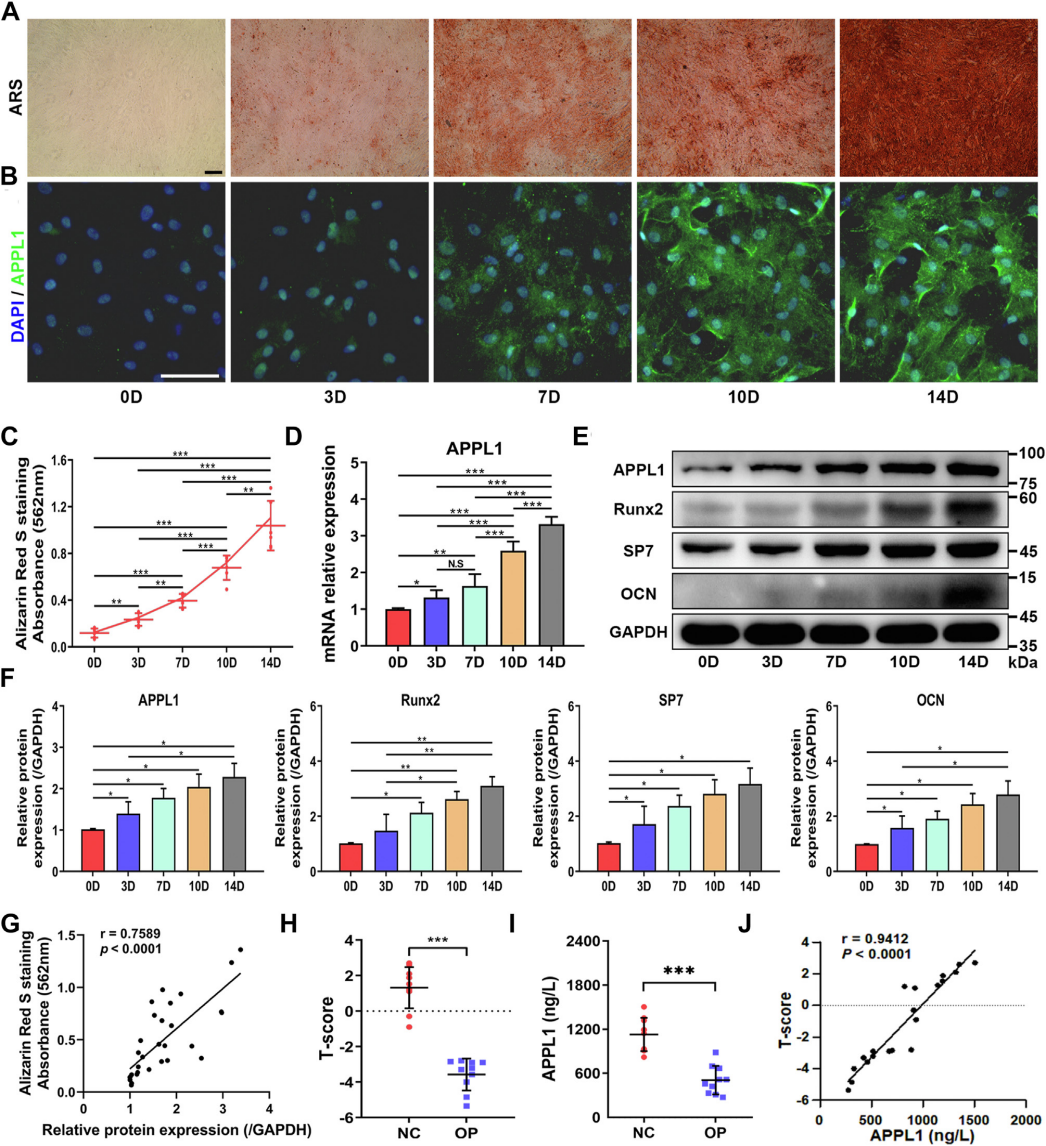
**APPL1 is upregulated during the osteogenic differentiation of MSCs.***A* and *C*, ARS staining and quantitative analyses of MSCs after osteogenic induction at different times. *B*, immunofluorescence staining of APPL1 during osteogenic differentiation of MSCs. *D*, the expression of APPL1 mRNA in MSCs at different osteogenic induction times was measured by qRT‒PCR. *E* and *F*, Western blotting was used to detect the expression of APPL1 and osteogenesis-associated marker proteins Runx2, SP7, and OCN at different osteogenic induction times in MSCs. *G*, Pearson correlation analysis showed that there was a correlation between APPL1 expression and ARS staining levels during osteogenic differentiation of MSCs. The data are shown as the mean ± SD (n = 6 per group). ∗*p* < 0.05, ∗∗*p* < 0.01, ∗∗∗*p* < 0.001, and N.S. indicates no significant difference. Scale bar = 100 μm. *H*, T-score differences between osteoporosis patients and healthy volunteers. *I*, the APPL1 concentration in MSCs lysates was detected by ELISA. *J*, Pearson correlation analysis showed that APPL1 expression was correlated with the severity of osteoporosis in different human MSCs. The data are shown as the mean ± SD (n = 10 per group). ∗∗∗*p* < 0.001 compared with the NC group. APPL1, Adaptor protein, phosphotyrosine interacting with PH domain and leucine zipper 1; ARS, alizarin red S; MSCs, mesenchymal stem cells.

**Supporting Figure S1:**
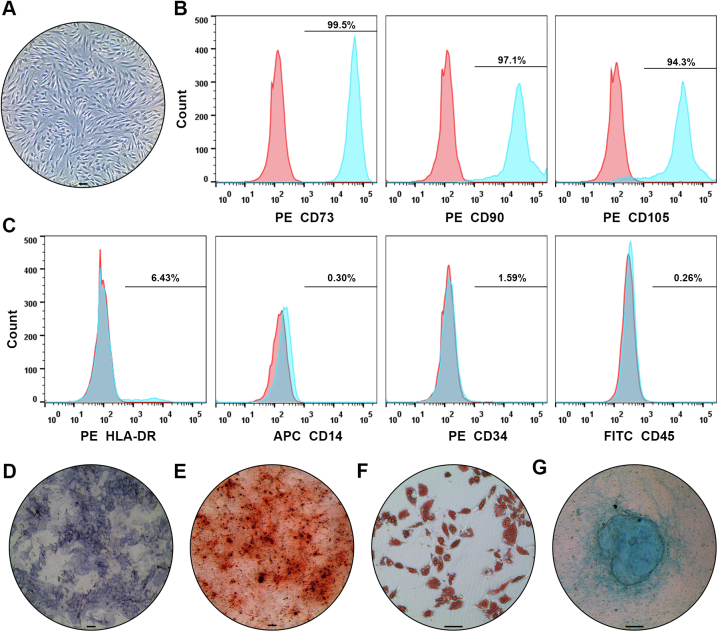
**Phenotypic identification and trilineage differentiation potential of MSCs.***A*, the morphology of MSCs. *B* and *C*, MSCs were positive for CD73, CD90, and CD105 but negative for HLA-DR, CD14, CD34, and CD45. *D*–*G*, the MSCs were differentiated into osteoblasts (ALP staining after 10 days; ARS staining after 14 days), adipocytes (ORO staining after 14 days) and chondrocytes (Alcian blue staining after 21 days). The data are shown as the mean ± SD (n = 6 per group). Scale bar = 50 μm.

MSCs differentiate into osteoblasts and play a critical role in the pathogenesis of osteoporosis (5). To further clarify the role of APPL1 in the osteogenic differentiation of MSCs in osteoporosis, MSCs were isolated and cultured from the bone marrow of osteoporotic patients and healthy volunteers (Fig. S1*A*). Flow cytometry was used to identify the phenotype of cells obtained from bone marrow. The results showed positive expression of CD73, CD90, and CD105 (Fig. S1*B*) and negative expression of CD14, CD34, CD45, and HLA-DR (Fig. S1*C*). MSCs are characterized by tri-lineage differentiation potential. We confirmed that the MSCs we isolated were able to differentiate into osteoblasts, chondrocytes, and adipocytes *in vitro* (Fig. S1, *D*–*G*). ARS staining of calcium deposition is an indicator of osteogenic differentiation. After 14 days of osteogenic induction, the osteogenic differentiation ability of MSCs was quantified by ARS staining. Consistent with our previous results, ARS staining showed a gradual increase in calcium nodule formation from days 0 to 14 (30) (Fig. 2, *A* and *C*). Subsequently, we evaluated the expression of APPL1 in MSCs during osteogenesis. qRT‒PCR showed that APPL1 significantly increased during osteogenic differentiation (Fig. 2*D*). Western blotting showed that the expression of the osteogenic markers Runx2, SP7, and OCN increased during osteogenic differentiation. Similarly, the expression of APPL1 increased gradually (Fig. 2, *E* and *F*). Moreover, we analyzed the correlation of the expression levels of APPL1 with ARS staining and found a strong positive relationship in the osteogenic differentiation of MSCs (Fig. 2*G*). In addition, the immunofluorescence showed that the expression of APPL1 increased with time during osteogenic differentiation (Fig. 2*B*). Finally, we recruited ten patients with osteoporosis and ten healthy volunteers to determine the relationship between the severity of osteoporosis and APPL1 (Table S2). T-score was measured in patients with osteoporosis and healthy volunteers, and the T-score was significantly lower in patients with osteoporosis (Fig. 2*H*). MSCs lysates were used to detect APPL1 concentration. The concentration of APPL1 decreased significantly in patients with osteoporosis and was positively correlated with the T-score (Fig. 2, *I* and *J*).

### APPL1 positively regulated the osteogenic differentiation of MSCs *in vitro*

**Figure 3:**
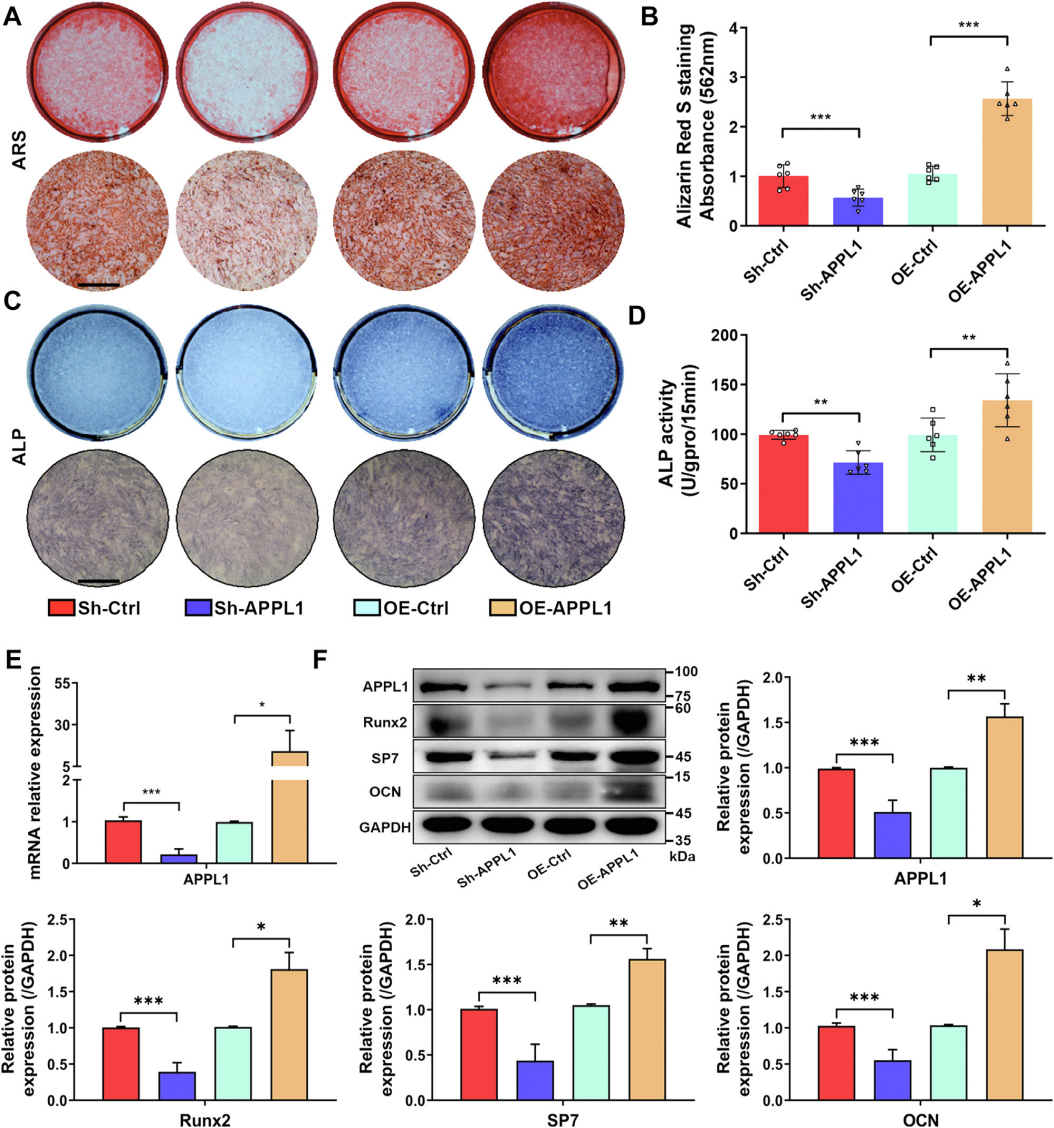
**APPL1 positively regulated the osteogenic differentiation of MSCs *in vitro*.***A* and *B*, ARS staining and quantitative analysis were performed after interference with APPL1 expression. *C* and *D*, ALP staining and activity observed after interference with APPL1 expression. *E*, relative mRNA expression of APPL1 after knockdown and overexpression of APPL1. *F*, APPL1, Runx2, SP7, and OCN were determined by Western blot after knockdown and overexpression of APPL1. The data are shown as the mean ± SD (n = 6 per group). ∗*p* < 0.05, ∗∗*p* < 0.01, ∗∗∗*p* < 0.001. Scale bar = 100 μm. ALP, alkaline phosphatase; APPL1, Adaptor protein, phosphotyrosine interacting with PH domain and leucine zipper 1; MSCs, mesenchymal stem cells.

To further investigate the effect of APPL1 on the osteogenic differentiation of MSCs, we designed and synthesized lentivirus to knock down and overexpress APPL1. qRT‒PCR and Western blotting verified that APPL1 had good knockdown and overexpression efficiency (Fig. 3, *E* and *F*). The results of ARS staining demonstrated that knocking down APPL1 expression decreased calcium nodule formation, whereas the overexpression of APPL1 increased calcium nodule formation during osteogenesis (Fig. 3, *A* and *B*). The results of ALP staining and activity measurement were consistent with ARS staining. Downregulation of APPL1 expression resulted in decreased staining and intensity during osteogenesis, while overexpression of APPL1 resulted in increased staining density and darker color during osteogenesis (Fig. 3, *C* and *D*). In addition, we detected the expression levels of the osteogenic markers Runx2, SP7, and OCN. The results showed that knocking down APPL1 inhibited the expression of Runx2, SP7, and OCN while overexpressing APPL1 increased the expression of Runx2, SP7, and OCN (Fig. 3*F*). In summary, APPL1 positively regulated the osteogenic differentiation of MSCs *in vitro*.

### MGP expression is increased after APPL1 knockdown in MSCs

**Figure 4:**
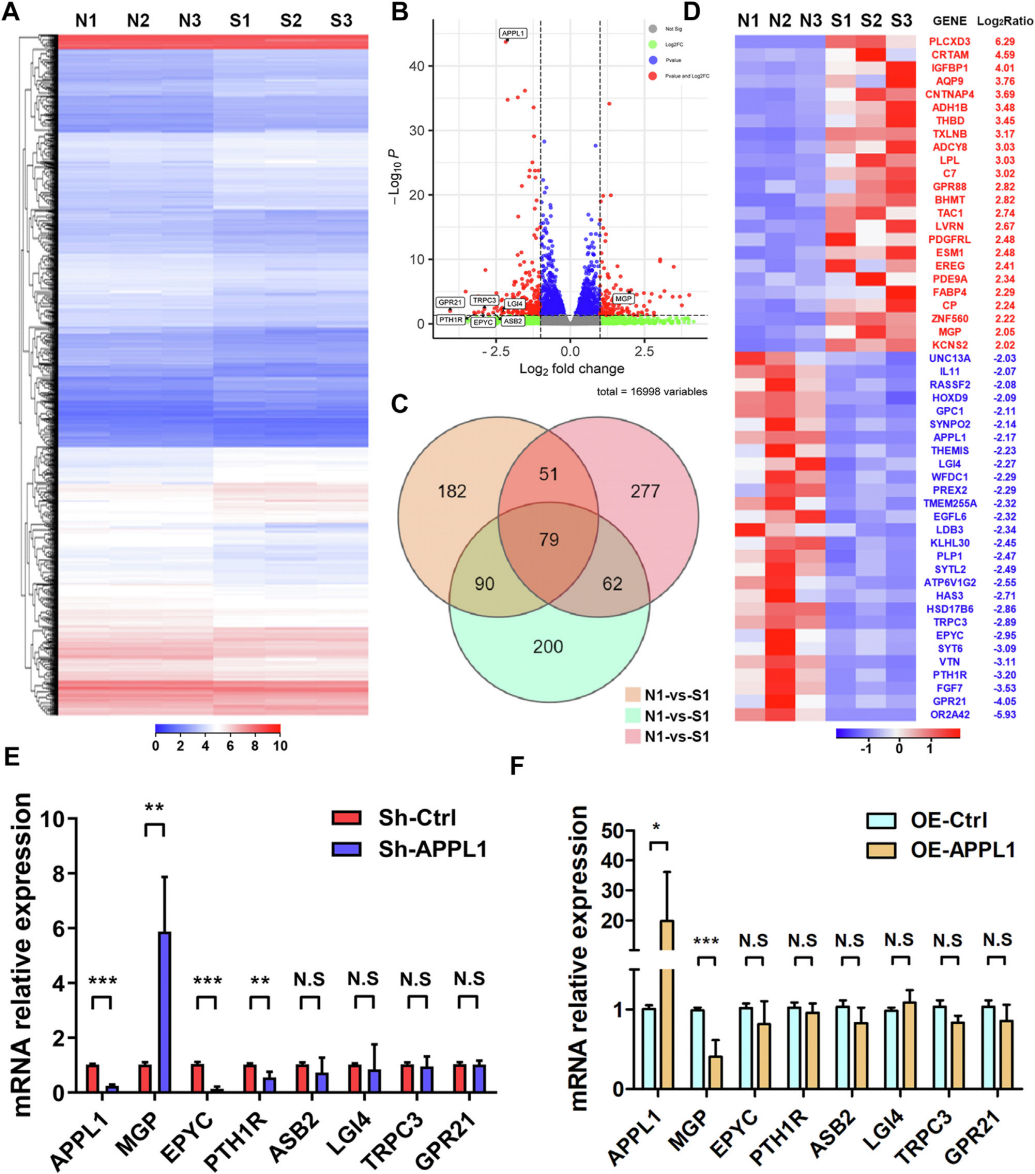
**MGP expression is increased after APPL1 knockdown in MSCs.***A* and *B*, cluster heatmap and volcano map for negative control and knockdown APPL1 treatment. *C*, Venn diagram of the negative control and APPL1 knockdown groups. *D*, the top 79 differentially expressed mRNAs identified by RNA sequencing. (n = 3 per group). FC ≥ 2，*p* ≤ 0.01. *E* and *F*, after knockdown and overexpression of APPL1, the expression of related mRNAs was measured by qRT‒PCR. The data are shown as the mean ± SD (n = 9 per group). ∗*p* < 0.05, ∗∗*p* < 0.01, ∗∗∗*p* < 0.001, and N.S. indicates no significant difference. APPL1, Adaptor protein, phosphotyrosine interacting with PH domain and leucine zipper 1; MGP, Matrix Gla protein; MSCs, mesenchymal stem cells.

**Supporting Figure S2:**
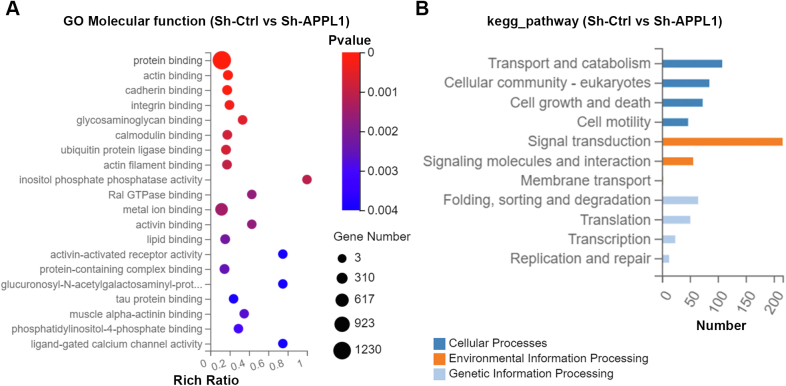
**Bioinformatic analysis of RNA sequencing data.***A*, GO analysis of the molecular function terms identified by RNA sequencing. *B*, KEGG pathway analysis after APPL1 knockdown. (n = 3 per group).

To explore the downstream mechanism of APPL1 regulating osteogenic differentiation of MSCs, we identified differentially expressed genes (DEGs) of MSCs from the control and APPL1 knockdown groups using RNA sequencing (RNA-seq). RNA-seq analysis identified 1886 DEGs. Compared with the control group, 864 upregulated genes and 1022 downregulated genes were found in the APPL1 knockdown group (Fig. 4, *A* and *B* and Table S3). As determined by GO molecular function analysis, 1230 mRNAs were enriched in protein binding, 58 mRNAs were enriched in cadherin binding, 37 mRNAs were enriched in calmodulin binding, and 31 mRNAs were enriched in integrin binding (Fig. S2*A*). Moreover, “Transport and catabolism”, “Signal transduction”, and “Folding, sorting and degradation” were the top enriched pathways as determined by KEGG analysis in Cellular Processes, Environmental Information Processing and Genetic Information Processing (Fig. S2*B*). Seventy-nine critical differentially expressed mRNAs were identified by Venn diagram analysis (Fig. 4, *C* and *D*). Then, through in-depth analysis of these genes, we selected MGP, EPYC, PTH1R, ASB2, LGI4, TRPC3, GPR21, and other differential genes related to osteogenesis, cartilage, calcification, extracellular matrix, calcium and phosphorus metabolism for further study. Furthermore, qRT‒PCR results showed that MGP was significantly upregulated after APPL1 knockdown, while EPYC and PTH1R were significantly downregulated, which was consistent with the RNA-seq data (Fig. 4*E*). After overexpression of APPL1, the expression of MGP decreased significantly (Fig. 4*F*). Overall, we found that MGP expression was regulated by APPL1 expression. MGP expression is increased after APPL1 knockdown by MSCs.

### MGP inhibited the osteogenic differentiation of MSCs by regulating the BMP2 pathway

**Figure 5:**
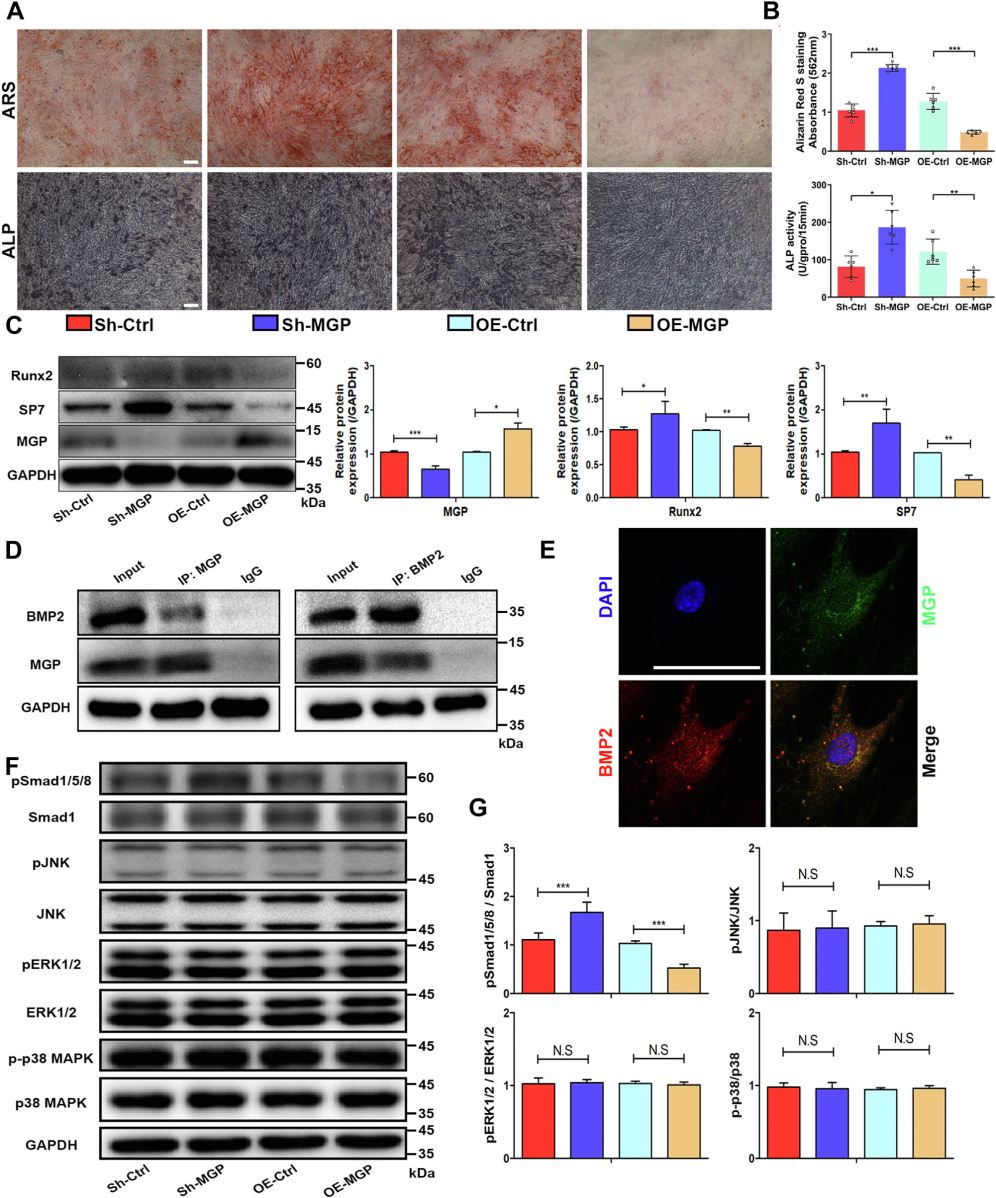
**MGP inhibited the osteogenic differentiation of MSCs by regulating the BMP2 pathway.***A* and *B*, ARS staining and quantitative analysis and ALP staining and activity measurements were performed after interference with MGP expression. *C*, protein levels of MGP, Runx2, and SP7 were determined by Western blot after knockdown and overexpression of MGP. *D*, MSCs lysates were immunoprecipitated with MGP, BMP2, or IgG antibodies. The interactions between the MGP and BMP2 proteins in MSCs were detected using Western blot analysis. *E*, immunofluorescence colocalization analysis of MGP and BMP2 in MSCs. *F* and *G*, activation levels of the Smad1/5/8, p38 MAPK, JNK, and ERK-1/2 signaling pathways in MSCs after interference with MGP expression were determined by Western blotting. The results of Western blotting were quantified as the intensity ratio of phosphorylated to nonphosphorylated proteins. The data are shown as the mean ± SD (n = 6 per group). ∗*p* < 0.05, ∗∗*p* < 0.01, ∗∗∗*p* < 0.001, and N.S. indicates no significant difference. Scale bar = 100 μm. ALP, alkaline phosphatase; ARS, alizarin red S; MGP, Matrix Gla protein; MSCs, mesenchymal stem cells.

**Supporting Figure S3:**
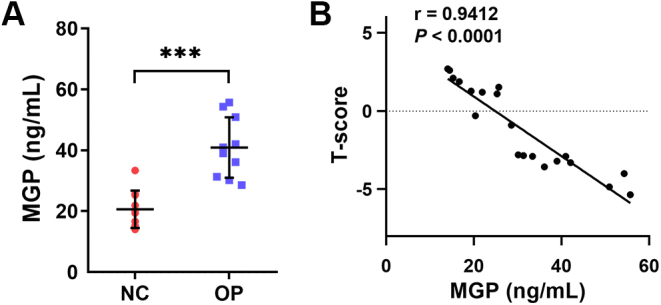
**Increased serum MGP levels in patients with osteoporosis.***A*, the concentration of MGP in serum was determined by ELISA. *B*, Pearson correlation analysis showed that serum MGP concentration was negatively correlated with T-score of osteoporosis. The data are shown as the mean ± SD (n = 10 per group). ∗∗∗*p* < 0.001 compared with the NC group.

To further clarify the effect of MGP on the osteogenic differentiation of MSCs, we designed and synthesized lentiviruses to knock down or overexpress MGP. The results of ARS and ALP staining demonstrated that knocking down MGP expression increased calcium nodule formation, whereas the overexpression of MGP decreased calcium nodule formation during osteogenesis (Fig. 5, *A* and *B*). In addition, Western blotting showed that knocking down MGP increased the expression of Runx2 and SP7 while overexpressing MGP inhibited the expression of Runx2 and SP7 (Fig. 5*C*). Moreover, we detected the concentration of MGP in the serum of patients with osteoporosis and healthy volunteers by ELISA. We found that the serum concentration of MGP in OP patients increased significantly, and was negatively correlated with the T-score of osteoporosis (Fig. S3, *A* and *B*). MGP has been reported to bind with BMP2 (29). To confirm this conclusion, we detected proteins binding to MGP by Co-IP. Consistent with the previous study, our results showed an interaction between MGP and BMP2. Next, we further detected the binding of endogenous BMP2 and MGP in MSCs by a Co-IP assay, and the results showed that endogenous BMP2 in MSCs could bind to MGP (Fig. 5*D*). In addition, the binding of BMP2 and MGP in cells was detected by immunofluorescence staining. Immunofluorescence colocalization analysis showed that BMP2 and MGP were closely colocalized (Fig. 5*E*). MGP combines with BMP2, so how does it regulate osteogenic differentiation? BMPs exert their effects through BMP receptors on the cell surface, activating both Smad and non-Smad pathways (31). Therefore, we detected the phosphorylation levels of Smad1/5/8, p38 MAPK, ERK-1/2 and JNK pathways by Western blot. Our results showed that the knockdown of MGP significantly increased the phosphorylation of Smad1/5/8, while overexpression of MGP significantly inhibited the phosphorylation of Smad1/5/8 (Fig. 5, *F* and *G*). Overall, these results indicated that MGP disturbed the activation of the Smad1/5/8 pathways by binding to BMP2 and eventually inhibited the osteogenic differentiation of MSCs.

### MGP is a key downstream target of APPL1-mediated osteogenic differentiation of MSCs

**Figure 6:**
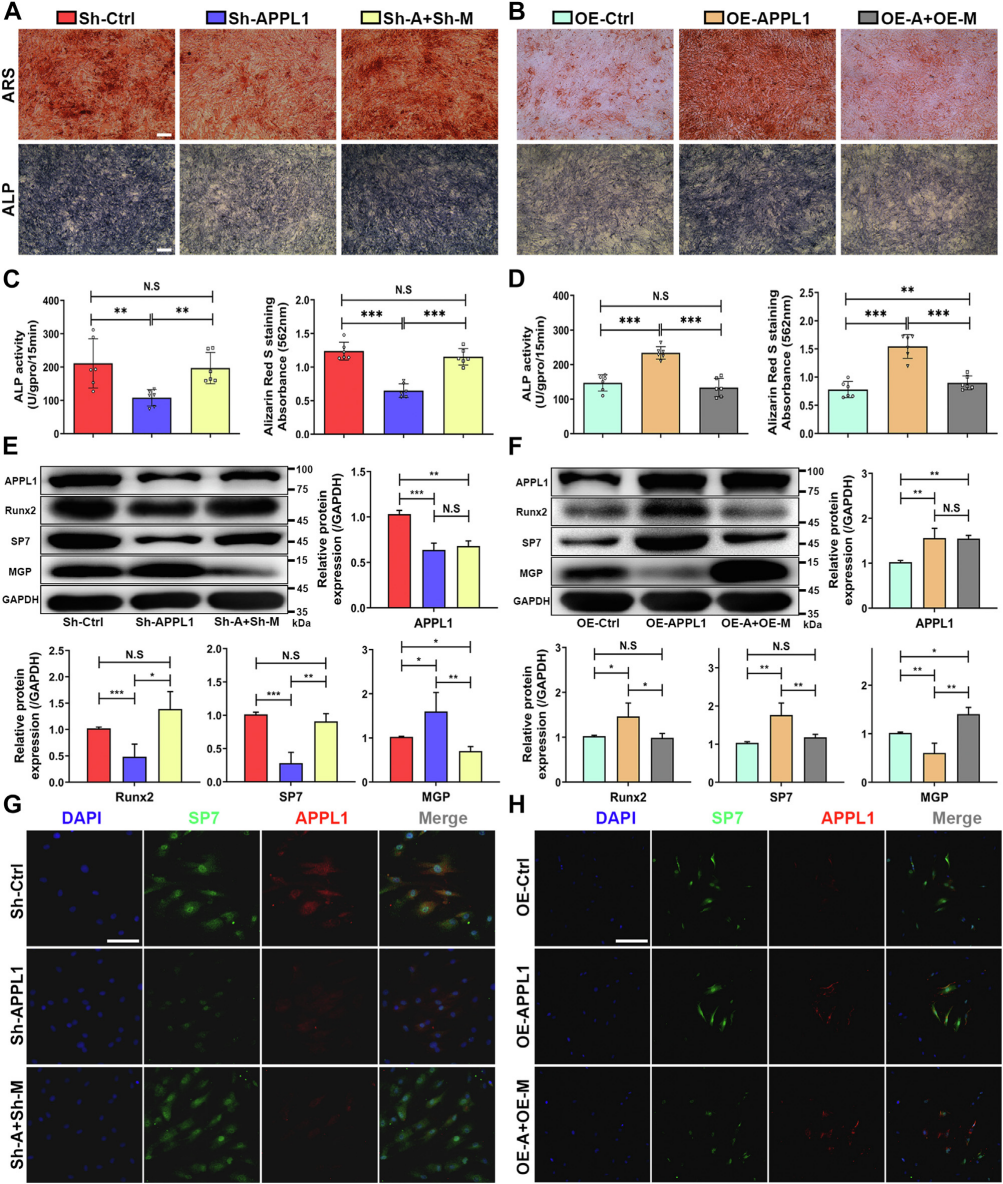
**MGP is a key downstream target of APPL1-mediated osteogenic differentiation of MSCs.***A*–*D*, ARS staining and quantitative analysis and ALP staining and activity measurements were performed after interference with APPL1 and MGP expression. *E* and *F*, protein levels of APPL1, MGP, Runx2, and SP7 were determined by Western blot. *G* and *H*, immunofluorescence staining of APPL1 and SP7 after interference with APPL1 and MGP expression. The data are shown as the mean ± SD (n = 6 per group). ∗*p* < 0.05, ∗∗*p* < 0.01, ∗∗∗*p* < 0.001, and N.S. indicates no significant difference. Scale bar = 100 μm. APPL1, Adaptor protein, phosphotyrosine interacting with PH domain and leucine zipper 1; MGP, Matrix Gla protein; MSCs, mesenchymal stem cells.

**Supporting Figure S4:**
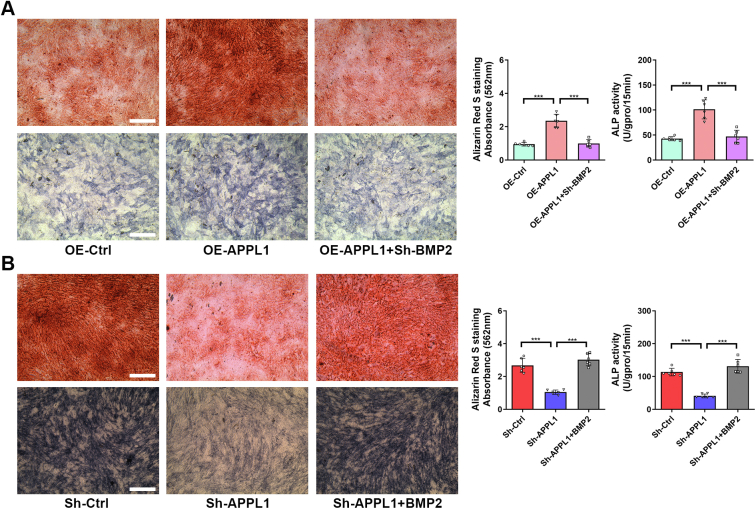
**APPL1 regulates osteogenic differentiation of MSCs through BMP2.***A* and *B*, ARS staining and quantitative analyses, ALP staining and activity measurements. The data are shown as the mean ± SD (n = 6 per group). ∗∗∗*p* < 0.001. Scale bar = 100 μm.

To further detect whether MGP is responsible for APPL1-mediated regulation of osteogenic differentiation of MSCs, we used Lentivirus to knock down or overexpress APPL1 and MGP. In our study, we found that the ARS and ALP staining decreased significantly after APPL1 knockdown. Simultaneous knockdown of APPL1 and MGP and osteogenic inhibition caused by APPL1 were blocked, resulting in enhanced ARS and ALP staining (Fig. 6, *A* and *C*). Moreover, simultaneously knocking down APPL1 and MGP upregulated the expression of Runx2 and SP7, reversing the downregulation caused by knocking down APPL1 alone. (Fig. 6*E*). At the same time, immunofluorescence staining showed that the knockdown of APPL1 and MGP enhanced the reduction in osteogenesis caused by the knockdown of APPL1 (Fig. 6*G*). In addition, ARS and ALP staining was enhanced after APPL1 overexpression, whereas after additional overexpression of MGP, the effect of APPL1 on promoting osteogenesis was reversed (Fig. 6, *B* and *D*). Western blotting results showed that the protein expression levels of Runx2 and SP7 were decreased after APPL1 and MGP were overexpressed at the same time, reversing the enhancement of protein expression caused by APPL1 overexpression solely (Fig. 6*F*). In addition, immunofluorescence staining showed that overexpression of APPL1 and MGP significantly inhibited the osteogenic enhancement induced by APPL1 overexpression (Fig. 6*H*). These results were consistent with ARS and ALP staining. Besides, we found that overexpression of APPL1 in MSCs resulted in enhanced osteogenic differentiation of MSCs. However, overexpression of APPL1 cannot lead to the enhanced osteogenic differentiation of MSCs in the situation of a knockdown of the expression of BMP2 (Fig. S4*A*). Combined with these results, it indicates that APPL1 promotes the osteogenic differentiation of MSCs through the BMP2 pathway. At the same time, after APPL1 knockdown, the oversupply of BMP2 could significantly increase ARS staining, ALP staining, and activity, thereby overcoming the reduction of APPL1 knockdown during osteogenic differentiation in MSCs (Fig. S4*B*). Taken together, our results indicate that MGP is a key downstream target of APPL1-mediated osteogenic differentiation of MSCs.

### APPL1 inhibited MGP expression by binding to the MGP promoter

**Figure 7:**
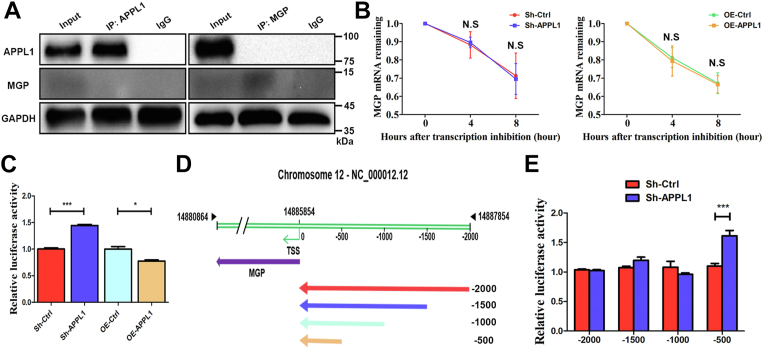
**APPL1 inhibited MGP expression by binding to the MGP promoter.***A*, MSCs lysates were immunoprecipitated with APPL1, MGP or IgG antibodies. The interactions between the APPL1 and MGP proteins in MSCs were detected using Western blot analysis. *B*, the RNA lifetime of MGP in MSCs transfected with knockdown or overexpressing APPL1 lentivirus was determined by monitoring transcript abundance after adding actinomycin D. *C*, the luciferase reporter vector subcloned with a 2.0-kb MGP promoter was transfected into 293T cells that were lentivirally transduced with scrambled or APPL1 shRNA and empty vector or APPL1 expression vector. After 24 h, luciferase activities were measured with Renilla for normalization. *D*, a series of 5′ deletions of the MGP promoter generated by PCR were subcloned into the pGL4.10 luciferase reporter vector. *E*, the different lengths of MGP promoter luciferase vectors were transiently transfected into 293T cells that were lentivirally transduced with scrambled or APPL1 shRNA. Luciferase activity was measured 24 h posttransfection with Renilla for normalization. The data are shown as the mean ± SD (n = 3 per group). ∗*p* < 0.05, ∗∗∗*p* < 0.001, and N.S. indicates no significant difference. APPL1, Adaptor protein, phosphotyrosine interacting with PH domain and leucine zipper 1; MGP, Matrix Gla protein; MSCs, mesenchymal stem cells.

To clarify the relationship between APPL1 and MGP, Co-IP was used to detect whether APPL1 directly or indirectly binds to MGP. The Co-IP results showed that APPL1 had no binding relationship with MGP on protein (Fig. 7*A*), which is consistent with the results of liquid chromatography combined with tandem MS (LC‒MS/MS) in our previous study (10). We confirmed that APPL1 knockdown increased both the mRNA and protein levels of the MGP gene, and APPL1 overexpression decreased the mRNA and protein levels of MGP in MSCs (Figs. 4, *E* and *F* and 6, *E* and *F*). Subsequently, we explored whether APPL1 affected mRNA transcription and/or stability of MGP. The actinomycin D assay showed that APPL1 expression had no significant effect on MGP mRNA stability (Fig. 7*B*). To further explore the effects of APPL1 on MGP transcription, we subcloned the 2.0 kb MGP promoter region into the firefly luciferase reporter gene plasmid. Luciferase assay results showed that APPL1 gene knockdown significantly enhanced luciferase activity, while APPL1 gene overexpression significantly decreased luciferase activity in 293T cells (Fig. 7*C*). Furthermore, to identify the region of the MGP promoter responsible for the regulation of MGP transcription by APPL1, we constructed luciferase reporter plasmids containing a series of truncated mutated MGP promoters (Fig. 7*D*). Luciferase activity analysis showed that silencing APPL1 significantly enhanced the transcriptional activity of the 0.5 kb MGP promoter (Fig. 7*E*). Altogether, these results indicate that the 0.5 kb region upstream of the transcription start site (TSS) of the MGP promoter is responsible for the regulation of MGP transcription by APPL1.

### APPL1 significantly reduced bone loss in a GIOP mouse model

**Figure 8:**
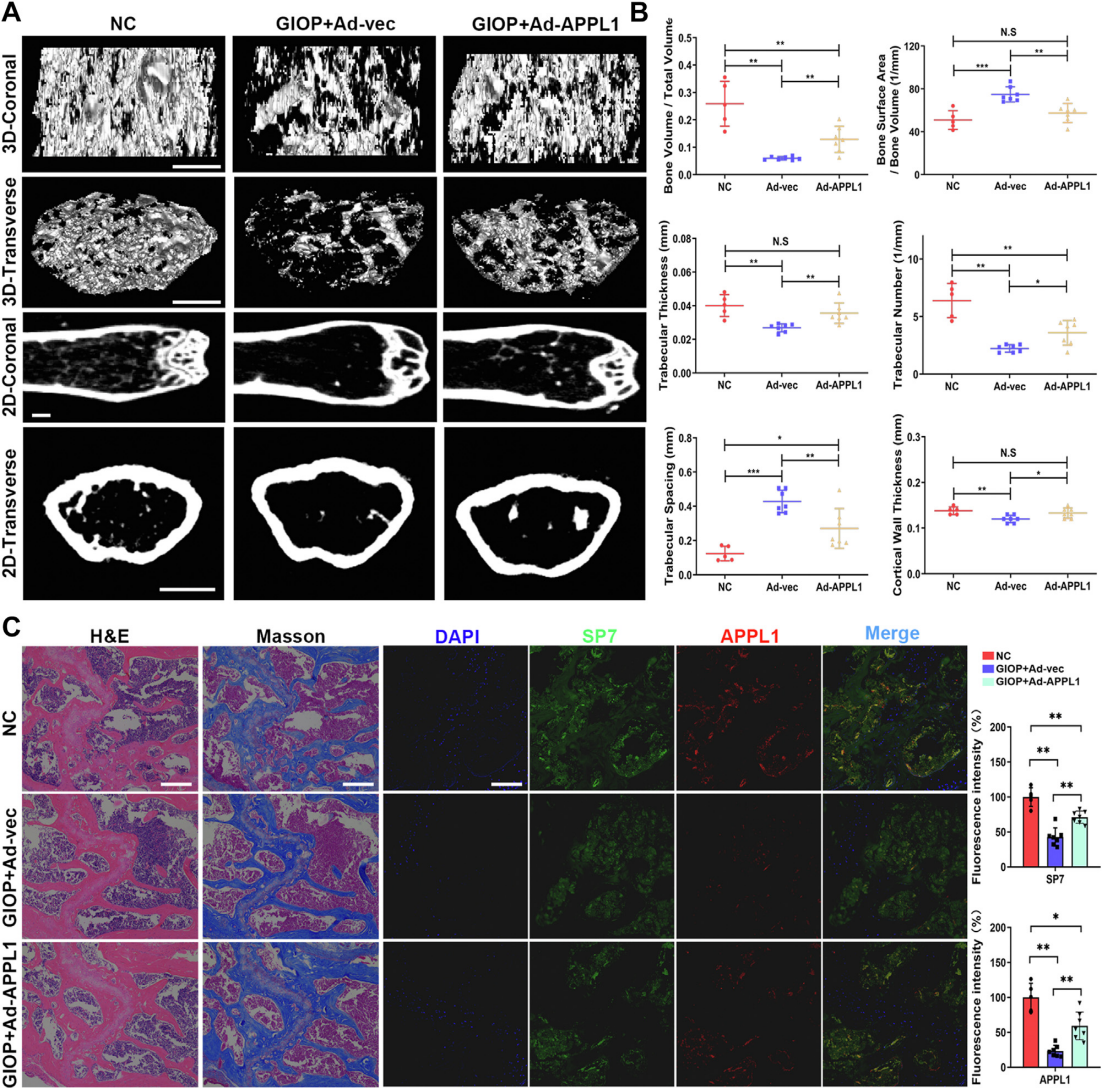
**APPL1 significantly reduced bone loss in a GIOP mouse model.***A*, representative micro-CT 2D and 3D reconstruction of trabecular bone. *B*, measurements of BSA/BV, BV/TV, Tb.Th, Tb.N, and Tb.Sp in proximal femur trabecular bone and Ct.Th in the mid-femur after 8 weeks of intervention. *C*, *left*: H&E staining and Masson staining of bone tissue in NC, GIOP+Ad-vec and GIOP+Ad-APPL1 groups of mice, *right*: immunofluorescence staining and quantitative analyses of SP7 and APPL1 in bone tissue of NC, GIOP+Ad-vec and GIOP+Ad-APPL1 groups of mice. The data are shown as the mean ± SD (NC group, n = 5; GIOP+Ad-vec group, n = 7; GIOP+Ad-APPL1 group, n = 7). ∗*p* < 0.05, ∗∗*p* < 0.01, ∗∗∗*p* < 0.001, and N.S. indicates no significant difference. Scale bar = 100 μm. APPL1, Adaptor protein, phosphotyrosine interacting with PH domain and leucine zipper 1; GIOP, glucocorticoid-induced osteoporosis; micro-CT, micro-computed tomography; NC, normal control.

To evaluate the role of APPL1 in the GIOP mouse model, adenoviruses overexpressing APPL1 were synthesized and injected into GIOP mice to observe the effect of APPL1 on bone mass. We performed micro-CT analysis to evaluate the bone structural features of mice in the normal control (NC), GIOP+Ad-vec, and GIOP+Ad-APPL1 groups. Overall, the results showed that compared with the NC group, the GIOP+Ad-vec group mice showed a significantly increased in bone loss, while the GIOP+Ad-APPL1 group mice had significantly less bone loss than the GIOP+Ad-vec group mice (Fig. 8*A*). APPL1 overexpression significantly increased the trabecular bone number and cortical thickness in GIOP mice (Fig. 8, *A* and *B*). Subsequently, the trabecular bone morphological parameters (BV/TV, BSA/BV, Tb.Th, Tb.N, and Tb.Sp) were analyzed (Fig. 8*B*). In addition, H&E and Masson staining of bone tissue demonstrated that the Tb.N was significantly increased in the APPL1 overexpression group, consistent with the results of micro-CT scanning (Fig. 8*C*). Moreover, the immunofluorescence staining and quantitative analyses of SP7 and APPL1 were significantly enhanced in mice of the GIOP+Ad-APPL1 group (Fig. 8*C*). Taken together, these findings suggest that APPL1 significantly reduced bone mass loss in GIOP mice and that APPL1 may be an important target for the diagnosis and treatment of osteoporosis.

## Discussion

In the current study, we confirmed that APPL1 expression was decreased in osteoporosis and positively regulated the osteogenic differentiation of MSCs. Mechanistically, reduced APPL1 impaired the osteogenic differentiation of MSCs by facilitating MGP expression to disrupt the BMP2 pathway in osteoporosis. In addition, the severity of clinical osteoporosis was negatively correlated with the expression of APPL1 in bone marrow MSCs, and overexpression of APPL1 in osteoporotic mice significantly alleviated bone loss. Therefore, our study suggested that APPL1 may be an important target for the diagnosis and treatment of osteoporosis.

Osteoporosis is the most common bone disease associated with aging, characterized by bone mass reduction and degeneration of bone microstructure, which increases bone fragility and leads to fracture (1). Maintaining bone mass balance depends on the mutual regulation of osteoblasts and osteoclasts, and the imbalance of osteoblasts and osteoclasts leads to osteoporosis (4). MSCs differentiate into osteoblasts and play a critical role in the pathogenesis of osteoporosis (5, 32, 33). The differentiation of MSCs lineage into osteoblasts is strictly regulated, and the dysfunction of MSCs differentiation often leads to ankylosing spondylitis, ossification of the posterior longitudinal ligament, and osteoporosis (34, 35, 36). In patients with osteoporosis, the osteogenic differentiation ability of MSCs was attenuated (1). Therefore, the mechanism of osteogenesis reduction of MSCs in osteoporosis should be elucidated to explore potential therapeutic targets for patients with osteoporosis.

APPL1 is a ligand of AdipoR and plays a central role in APN and insulin signaling (7). Most previous studies have shown that MSCs enhance osteogenic differentiation by promoting the secretion of APN (37, 38, 39). However, the role of APPL1 in osteoporosis has not yet been elucidated. Our previous study has shown that the impairment of APPL1/myoferlin facilitates adipogenic differentiation of MSCs by blocking autophagic flux in osteoporosis (10). In this study, we found that APPL1 expression was downregulated in osteoporosis patients and mice. Besides, the severity of clinical osteoporosis was negatively correlated with APPL1 expression in bone marrow MSCs. Moreover, APPL1 overexpression effectively alleviated osteoporosis and promoted osteogenic differentiation of MSCs, whereas APPL1 knockdown attenuated osteogenic differentiation of MSCs. Combined with our previous study (10), it suggests that APPL1 may be an important molecule leading to the abnormal balance of adipogenic and osteogenic differentiation of MSCs in osteoporosis.

To elucidate the downstream mechanism of APPL1 regulating osteogenic differentiation of MSCs, we performed the RNA-seq analysis and found that APPL1 could significantly inhibit the expression of MGP. MGP is an inhibitor of arterial and cartilage calcification, and MGP deficiency in mice leads to premature bone calcification, calcification of noncalcified cartilage such as the trachea, and severe vascular calcification leading to premature death (14). Interestingly, osteoblasts in nonunion fractures were positive for MGP expression, whereas osteoblasts in normal union fractures were not (40). Studies have shown that osteogenic and chondrogenic differentiation of C3H10T1/2 cells was inhibited and BMP2 activity was decreased in C3H10T1/2 cells overexpressing MGP (41). However, the effect of MGP on the osteogenic differentiation of MSCs is still unclear. In our study, we found that MGP decreased the osteogenic differentiation of MSCs. Besides, we confirmed that MGP can bind to BMP2 protein and disturb the activation of BMP2 downstream pathways, which was consistent with a previous study (42). BMP2 signaling pathways play fundamental roles in embryonic skeletal development and bone homeostasis (20, 21). MSCs positively regulate osteogenesis mainly through BMP/Smad signal transduction (43, 44). In the current study, we illustrated the mechanism of APPL1 regulating osteogenic differentiation of MSCs, confirming that APPL1 inhibited the expression of MGP, thereby reducing the binding of MGP to BMP2 and activating the downstream pathway of BMP2.

To further detect the effect of APPL1 on the osteogenic differentiation of MSCs *in vivo*, we constructed a GIOP mouse model by injecting dexamethasone. The GIOP model reduces cancellous bone mainly by inhibiting bone formation (45). In a previous study, Weinstein *et al.* (46) reported that glucocorticoid treatment of mice for 27 days reduced cancellous bone mass and markers of bone formation, but did not alter markers of bone resorption. In our study, we found that the cortical bone and cancellous bone of mice were significantly reduced after regular dexamethasone injection, and the GIOP model was successfully established. After 8 weeks of injection, markers of bone formation and bone mass were significantly decreased, but the markers of bone resorption were not significantly upregulated. In this study, we demonstrated that APPL1 reduced glucocorticoid-induced bone loss by promoting osteogenic differentiation of MSCs *in vivo*, and we emphasized that our animal model of osteoporosis can be used to investigate osteogenic differentiation of osteoporosis.

A variety of cells in the bone microenvironment, such as osteoclasts, osteoblasts, osteocytes, bone lining cells and vascular endothelial cells, are involved in the regulation of bone homeostasis (47). The repair potential of bone and its surrounding microenvironment is associated with cells such as osteoblasts, osteoclasts, inflammatory cells, and endothelial cells (48). The imbalance between osteoclasts and osteoblasts leads to osteoporosis and other diseases (4). Our previous and current studies have confirmed that APPL1 can inhibit the differentiation of MSCs into adipocytes and promote the differentiation of MSCs into osteoblasts (10). Subsequently, in our study, we found that GIOP model stimulated the formation of osteoclast, while overexpression of APPL1 inhibited the formation of osteoclast. However, the mechanism by which APPL1 regulates osteoclast differentiation remains unclear and needs to be further explored.

At present, bisphosphonates and calcitonin, which work by inhibiting osteoclast function, are the main drugs used to treat osteoporosis. however, there are fewer drugs for osteogenic enhancement (4, 49). In recent years, studies have shown that osteogenic differentiation is a promising target for the treatment of osteoporosis (50, 51). Therefore, there is great potential to investigate anti-osteoporosis drugs that promote the osteoblast differentiation of MSCs. In this study, we found that APPL1 was able to enhance osteogenic differentiation of MSCs *in vitro* and reduced glucocorticoid-induced bone loss *in vivo*, highlighting its potential use may be an alternative drug to treat osteoporosis by modulating osteogenic differentiation.

In conclusion, we identified APPL1 may be an important regulator in osteoporosis, which inhibited the expression of MGP, activated the BMP2 pathway, and promoted the osteogenic differentiation of MSCs. However, this study still has some limitations. For example, maintaining bone mass balance depends on the mutual regulation of osteoblasts and osteoclasts. Therefore, further research is needed to detect the effect of APPL1 in osteoclast differentiation. On the other hand, clinical trials are needed to further explore the application of APPL1 in osteoporosis.

## Experimental procedures

### Ethics statement

This study was approved by the Ethics Committee of the Eighth Affiliated Hospital, Sun Yat-sen University, Shenzhen, China (Approval No. 2021r209). All patients and volunteers were aware of the study procedures and potential risks, and informed consent was obtained. The animal experiments were approved by the Animal Protection and Use Organization Committee of Sun Yat-sen University, Guangzhou, China (Approval No. 2021d184). All the experiments performed in this study were conducted in accordance with the committee’s regulations and guidelines.

### Isolation and culture of human bone marrow MSCs

MSCs were isolated, purified, and cultured from bone marrow by density gradient centrifugation at 15,000*g* for 30 min as previously described (34). Then, the MSCs were resuspended in Dulbecco’s modified Eagle medium (DMEM; GIBCO, C11995500BT) containing 10% fetal bovine serum (FBS; Thermo Fisher, 10099141) and inoculated in 75 cm^2^ cell culture flasks (Corning, 430641) in incubators at 37 °C and 5% CO_2_. After 48 h, the culture medium was replaced to remove nonadherent cells. The medium was replaced every 3 days. When the cells reached 80 to 90% confluence, the MSCs were digested with 0.25% trypsin and 0.02% EDTA (Jingxin Biotech, GX25200) and transferred to new cell culture flasks at passage 1. MSCs in passages 3 to 5 were used for subsequent experiments.

### Flow cytometric analysis

The expression of seven surface markers of MSCs was analyzed by flow cytometry to detect the purity of MSCs. Antibodies against CD14 (Biolegend, 301808), CD34 (Biolegend, 343505), CD45 (Biolegend, 368507), CD73 (BD, 550257), CD90 (BD, 555596), CD105 (BD, 560839), and HLA-DR (BD, 555812) were purchased. Passage 3 MSCs were digested with trypsin and placed in round bottom polystyrene tubes (Falcon, 352054). Cell staining buffer (Biolegend, 420201) was added, and the cells were suspended at a concentration of 1 × 10^7^/ml. Then, an appropriate amount of antibody was added according to the manufacturer's protocol, and the cells were incubated at 4 °C for 30 min in the dark. After centrifugation at 300*g* for 5 min, the cells were resuspended in cell staining buffer and detected and analyzed by flow cytometry (BD FACSCelesta).

### Osteogenic differentiation of MSCs

For osteogenic differentiation, MSCs were seeded in 12-well plates at a density of 1.5 × 10^4^ cells/cm^2^ in DMEM with 10% FBS. After 24 h, the normal medium was replaced with an osteogenic differentiation medium. The osteogenic differentiation medium was prepared by adding 1% penicillin‒streptomycin (Jingxin Biotech, GX15140), 50 μM ascorbic acid (Sigma‒Aldrich, PHR1008), 10 mM β-glycerol phosphate (Sigma‒Aldrich, G5422), and 0.1 μM dexamethasone (Sigma‒Aldrich, D4902). Then, the induction medium was changed every 3 days, and the osteogenesis of MSCs was detected by alkaline phosphatase (ALP) staining, ALP activity, and alizarin red S (ARS) staining assays.

### ALP activity, ALP staining and ARS staining

ALP staining and ALP activity measurements were performed on day 10 after osteogenic differentiation, while ARS staining was performed on day 14. Briefly, cells were lysed with 100 μl of lysis solution per 12-well plate. The lysis solution was composed of RIPA lysis buffer (ComWin Biotech, CW2333S), 1% phenylmethanesulfonyl fluoride (PMSF; Beyotime, ST506) and phosphatase inhibitors (Sigma‒Aldrich, P5726). Then, the lysate was collected and centrifuged at 18,000*g* for 15 min. A total of 30 μl of supernatant was used to detect ALP activity. The alkaline phosphatase assay kit (Jiancheng Biotech, A059-2) was used to detect ALP activity according to the manufacturer's instructions. Then, 50 μl of solution A and 50 μl of solution B were added to the supernatant and standard solution and incubated in a 37 °C incubator for 15 min. After adding 150 μl of stop solution, the ALP absorbance at 520 nm was measured by a Varioskan LUX multimode microplate reader (Thermo Scientific, Varioskan LUX). In addition, the total protein concentration was determined by a BCA protein assay kit (ComWin Biotech, CW0014S). Finally, ALP activity was calculated by ALP absorbance and total protein concentration in units per gram of protein per 15 min (U/g pro/15 min). For ALP staining, the induced MSCs were washed three times with PBS and fixed with 4% paraformaldehyde (Macklin, P804536) for 30 min. Then, the cells were stained with the BCIP/NBT alkaline phosphatase color development kit (Beyotime, C3206) for 15 min at 37 °C in the dark according to the instructions. For ARS staining, the MSCs were fixed with 4% paraformaldehyde for 30 min. The induced MSCs were then stained with ARS (Solarbio, G8550) dye for 15 min at room temperature. Thereafter, the stained cells were observed and photographed under a microscope. For ARS quantification, the cells were extracted with 1 ml 10% cetylpyridinium chloride monohydrate (CPC; Sangon Biotech, A600106) at room temperature for 60 min, and then the absorbance at 562 nm was measured.

### Chondrogenic differentiation of MSCs and Alcian blue staining

MSCs were planted in high-density (5 × 10^5^ cells) micromasses in a chondrogenic medium. The chondrogenic medium was composed of high-glucose DMEM (Cienry, CR-12800), 1% ITS Premix (Corning, 354351), 1% penicillin‒streptomycin, 50 μM ascorbic acid, 10 ng/ml recombinant human transforming growth factor (TGF) beta 3 (rhTGF-β3; R&D, 243-B3-010), 1 mM sodium pyruvate (Sigma‒Aldrich, P5280), and 0.1 μM dexamethasone. The cells were cultured in a 37 °C, 5% CO_2_ incubator, and the medium was exchanged every 2 to 3 days. After 21 days of induction, the chondrogenic cells were processed for Alcian blue staining (Sigma‒Aldrich, 66011) according to the manufacturer’s protocol.

### Adipogenic differentiation of MSCs and oil red O staining

For adipogenic induction, MSCs were seeded at 1.5 × 10^4^ cells/cm^2^ in a 12-well plate and cultured until they reached 90% confluence. The medium was replaced with adipogenic medium and incubated at 37 °C and 5% CO_2_, with the medium changed every 3 days. The adipogenic medium was composed of high-glucose DMEM, 10% FBS, 1% penicillin‒streptomycin, 10 μg/ml human insulin (BI, 41-975-100), 1 μM dexamethasone, 0.5 mM 3-isobutyl-1-methylxanthine (IBMX; Sigma‒Aldrich, I7018), and 0.2 mM indomethacin (Sigma‒Aldrich, I7378). After 2 weeks of adipogenic induction, MSCs were fixed with 4% paraformaldehyde for ORO staining. The ORO staining (Beyotime, C0157S) solution was prepared according to the kit instructions and stained at room temperature for 15 min. Then, the dye was removed, and nonspecific staining was washed away with PBS. Thereafter, the stained cells were observed and photographed under a microscope.

### Immunofluorescence staining

The medium in osteogenic differentiated MSCs was removed and washed three times with PBS. Cells were fixed with 4% paraformaldehyde for 30 min and permeated with 0.5% Triton X-100 (Macklin, T824275) for 30 min, and 5% normal goat serum (Solarbio, SL038) was used to block cells for 30 min at room temperature. Then, an appropriate amount of immunostaining primary antibody dilution buffer (Beyotime, P0103) and primary antibody against APPL (Abcam, ab180140, 1:250) were added and incubated overnight at 4 °C. After washing with PBS three times, goat anti-rabbit IgG H&L (Alexa Fluor 555) (Abcam, ab150078, 1:1000) and/or goat anti-mouse IgG H&L (Alexa Fluor 488) (Abcam, ab150113, 1:1000) was added and incubated for 60 min at room temperature. Thereafter, the nuclei of MSCs were counterstained with 4′,6-diamidino-2-phenylindole dihydrochloride (DAPI, Beyotime, C1006). Finally, the images were observed and collected under a fluorescence microscope (Leica DMI4000 B).

### Western blot

Cell lysis and protein concentration were determined in the same way as for ALP activity. Equal amounts of protein samples were diluted with 5 × SDS‒PAGE Sample Loading Buffer (Beyotime, P0015) and subsequently transferred to polyvinylidene difluoride (PVDF) membranes (Merck Millipore, IPVH00010) after SDS‒PAGE. Then, the membranes were transferred to Tris buffered saline with Tween (TBST) (10 mM Tris-HCl, 15 mM NaCl, 0.05% Tween-20, pH 7.5) solution and washed three times. After that, the membranes were blocked with 5% skim milk (Wako, 190-12865), incubated for 60 min at room temperature, and then incubated with primary antibodies against GAPDH (CST, 5174S, 1:1000), APPL (Abcam, ab180140, 1:2000), osteocalcin (OCN) (Abcam, ab133612, 1:1000), Sp7 transcription factor (SP7) (Abcam, ab209484, 1:1000), and MGP (Proteintech, 60055, 1:1000) overnight in a 4 °C refrigerator. After washing three times with TBST, the corresponding species of horseradish peroxidase (HRP)-conjugated secondary antibodies goat anti-mouse IgG (ComWin Biotech, cw0102s, 1:3000) or goat anti-rabbit IgG (ComWin Biotech, cw0103s, 1:3000) were added and incubated for 60 min at room temperature on a shaker. Finally, the membranes were washed three times with PBS, and the immunoreactive protein bands were detected by immobilon Western chemiluminescent HRP substrate (Merck Millipore, WBKLS0500). Relative quantitative analysis was performed by ImageJ software.

### Real-time qRT‒PCR

Sample RNA was extracted according to the manufacturer's protocol in the RNA quick purification kit (ESscience, RN001). Subsequently, equal amounts of RNA were reverse-transcribed into complementary DNA using the PrimeScript RT reagent Kit (TAKARA, RR037A). Then, qRT‒PCR was performed using TB Green Premix Ex Taq (TAKARA, RR420A) according to the manufacturer's protocol with the 7500 Real-Time PCR System (Applied Biosystems). Each sample was tested in triplicate. GAPDH was used as the normalization control, and the relative mRNA expression of genes was calculated using 2 -ΔΔCt values. The target genes and corresponding forward and reverse primer sequences are listed in Table S1.

### RNA sequencing and data analysis

After APPL1 knockdown, MSCs were induced to undergo osteogenesis for 5 days, and total RNA was extracted by TRIzol reagent (Thermo Fisher, 15596018). Then, the extracted RNA was dissolved in 25 μl of RNase-free water. Total RNA was subsequently identified and quantified using a NanoDrop and Agilent 2100 bioanalyzer (Thermo Fisher Scientific). Afterward, purified mRNA was reverse-transcribed into cDNA. cDNA library construction and sequencing were performed by the Beijing Genomics Institute (BGI-Shenzhen) on the BGISEQ500 platform. For sequencing data analysis, SOAPnuke (v1.5.2) was used to filter the data, and HISAT2 (v2.0.4) was used to map clean reads to the reference genome. Next, gene expression was calculated by RSEM (v1.2.12) after Bowtie2 (v2.2.5) alignment. After that, DESeq2 (v1.4.5) was used for differential expression analysis, and the parameter fold change (FC) was ≥2 and the adjusted *p* value was ≤0.01. Pheatmap (V1.0.8) was used to draw a heatmap according to the gene expression of different samples. Finally, Venn diagram creation, gene ontology (GO, http://www.geneontology.org/) and Kyoto Encyclopedia of Genes and Genomes (KEGG, https://www.kegg.jp/) enrichment analyses were performed using BGI’s Dr Tom Multi-Omics Data Visualization System.

### ELISA

First, the APPL1 antibody was coated in solid-phase support. Then, cell lysates were collected, and equal amounts of protein and standard solution were added to the microplates and incubated for 30 min at room temperature. After thorough washing, the cells were incubated with secondary antibodies for 30 min. Finally, color developing solution was added, and after 15 min of incubation followed by the addition of the stop solution, OD values were measured with a microplate reader, and standard curves were established with standard samples. The exact APPL1 concentration was calculated from the standard curve.

### Lentivirus construction and infection

The method of lentivirus construction and infection was consistent with a previous study (29). Briefly, lentiviruses encoding short hairpin RNA (shRNA) targeting APPL1 (Sh-APPL1, 5′-GCATTGTTAGAACCTCTACTT-3′) and MGP (Sh-MGP, 5′-AGCCTGTCCACGAGCTCAATA-3′) were constructed. The negative control shRNA sequence was 5′-TTCTCCGAACGTGTCACGTTTC-3′ (Sh-Ctrl). APPL1-and MGP-overexpressing lentiviruses and their vector controls (OE-APPL1, OE-MGP and OE-Ctrl) were purchased from OBiO (Shanghai, China). Lentivirus (10^9^ TU/ml) and polystyrene (5 μg/ml, Sigma) were added to the culture medium and incubated with MSCs for 24 h at a multiple of infection (MOI) of 30. After 72 h, the knockdown and overexpression efficiencies were analyzed by qRT‒PCR and Western blotting.

### Coimmunoprecipitation

After the intervention, MSCs samples were rapidly collected and dissolved on ice for 30 min in IP lysis buffer (Beyotime, P0013) containing 1% PMSF and phosphatase inhibitor. Cell lysates were collected after centrifugation at 15,000*g* for 15 min. The cell extracts were then incubated overnight at 4 °C with antibodies against APPL1, MGP, BMP2, or their IgG controls. Magnetic beads were added and incubated for 3 h at 4 °C according to the Dynabeads Protein G Immunoprecipitation Kit (Thermo Fisher, 10007D) manufacturer's protocol. Magnetic beads were collected, washed, and resuspended in 60 μl containing 1% PMSF and phosphatase inhibitor RIPA lysis buffer. Finally, the sample was added to the SDS-PAGE sample loading buffer and boiled for 10 min, and Western blotting was performed.

### RNA decay assay

After transfection with lentivirus, MSCs were added to an osteogenic induction medium and cultured for 3 days. Then, actinomycin D (5 μg/ml) was added. Total RNA was extracted at 0, 4, and 8 h with TRIzol reagent, and the relative expression was measured by qRT‒PCR as described previously (34).

### Dual-luciferase reporter assay

MGP promoter sequences −2 kb relative to the TSS and different truncated fragments were synthesized and cloned into the pGL4.10 vector. 293T cells were transfected with the vectors described above, and lentiviruses were transfected with Lipofectamine 3000 (Invitrogen, L3000015) to silence and overexpress APPL1. All cells were transfected with pRL-TK plasmid as an internal control. Luciferase activity was measured using a dual luciferase reporter assay kit (Vazyme, DL101).

### Construction of glucocorticoid-induced osteoporosis model

Dexamethasone-treated C57BL/6J mice were used to establish a GIOP mouse model. Briefly, 2 mg/kg dexamethasone was injected intramuscularly into the hindlimb three times a week for 8 weeks. A total of 6 × 10^11^ APPL1-overexpressing or control adenoviruses were injected *via* the tail vein. The negative control group was injected with the same volume of normal saline. After 8 weeks, femurs were harvested for micro-CT analysis, H&E staining, Masson’s Trichrome (Masson) staining, immunohistochemistry, and immunofluorescence.

### Micro-computed tomography (micro-CT)

The experimental mice were humanely sacrificed by cervical dislocation after anesthesia, and the femurs were harvested and fixed with 4% paraformaldehyde for 36 h before Micro-computed tomography (micro-CT) analysis. The full-length femur was then scanned by the Siemens Inveon PET/CT Multimodality System. The parameters were set to a tube voltage of 80 kV and a tube current of 500 μA, and the effective high resolution was 9.56 μM. Subsequently, 50 scanning layers of the distal femur 50 mm below the growth plate were selected to measure the morphological parameters and three-dimensional reconstruction. Afterward, trabecular parameters were recorded, including bone volume/total volume (BV/TV), bone surface area/bone volume (BSA/BV), trabecular thickness (Tb.Th), trabecular number (Tb.N), trabecular spacing (Tb.Sp), and cortical wall thickness (Ct.Th).

### H&E and Masson staining

The femurs of mice were fixed for 36 h and decalcified with EDTA-decalcifying fluid (BOSTER, AR1071) for 3 weeks. After that, paraffin embedding was performed, and 5-μm sections were used for H&E and Masson staining. The sections were dewaxed in xylene, rehydrated in gradient ethanol, and then rinsed in distilled water. For H&E staining, sections were stained in hematoxylin for 8 min. The samples were rinsed with running water for 1 h and dehydrated in 70% and 90% alcohol for 10 min each. Subsequently, femur sections were stained with alcohol eosin for 3 min. For Masson staining, bone sections were stained according to the manufacturer's protocol (Solarbio, G1340). Finally, the stained sections were dehydrated with alcohol, cleared with xylene, and sealed with gum.

### Immunohistochemistry and tissue immunofluorescence staining

For immunohistochemistry, sections of bone tissue were treated with trypsin and pepsin to repair antigens, incubated with 3% H2O2 for 20 min to block endogenous peroxidase activity, and then blocked with 5% normal goat serum for 60 min at room temperature. Afterward, the sections were incubated with primary antibody at 4 °C overnight. Secondary antibody incubation and color development were performed using an SP rabbit and mouse HRP kit (DAB, ComWin Biotech, CW2069S) according to the kit protocol. For tissue immunofluorescence staining, after dewaxing and hydration, the sections were permeated with 1% Triton X-100. Then, antigens were repaired in citrate buffer (ComWin Biotech, CW0128S) and blocked in normal goat serum, followed by overnight incubation with primary antibody at 4 °C. After three washes with PBS, fluorescein-conjugated secondary antibody was added and incubated for 60 min at room temperature. Thereafter, the nuclei were counterstained with DAPI. Finally, the stained sections were dehydrated and fixed, and images were taken under a fluorescence microscope (Leica DM6 B).

### Statistical analysis

All results were determined based on at least three independent experiments. Statistical analysis was performed using IBM SPSS Statistics (version 20.0). The Shapiro‒Wilk test was used to test normality, and data with *p* > 0.05 were considered to conform to a normal distribution. Two-group comparisons were performed using a two-tailed Student's *t* test, and multiple comparisons were performed using one-way ANOVA, followed by Bonferroni's comparison. Data are expressed as the mean ± standard deviation (SD). *p* < 0.05 was considered statistically significant. The n values indicate the number of samples in each experiment. The levels of significance in the tables and figures are labeled as follows: ∗*p* < 0.05, ∗∗*p* < 0.01, ∗∗∗*p* < 0.001, and N.S. indicates no significant difference.

## Data availability

The datasets generated during this study are available from the corresponding author on reasonable request.

## Supporting information

This article contains supporting information.

## Conflict of interest

The authors declare that the research was conducted in the absence of any commercial or financial relationships that could be construed as a potential conflict of interest.
