# Supplementary material for: Reduced APPL1 impairs osteogenic differentiation of mesenchymal stem cells by facilitating MGP expression to disrupt the BMP2 pathway in osteoporosis
Source: J Biol Chem. 2023 May 13;299(6):104823. doi: 10.1016/j.jbc.2023.104823 (PMC10318529; doi:10.1016/j.jbc.2023.104823)
Supplement: Supporting Table S1 [file mmc1.doc]

**Table S1. Primers for qRT‒PCR.**

| Gene | Forward primer (5’-3’) | Reverse primer (5’-3’) |
| --- | --- | --- |
| GAPDH | GGAGCGAGATCCCTCCAAAAT | GGCTGTTGTCATACTTCTCATGG |
| APPL1 | GCTTTGTTAGAACCTCTACTGGG | TCAGGCAGATATAAAGGGTCACT |
| MGP | TCCGAGAACGCTCTAAGCCT | GCAAAGTCTGTAGTCATCACAGG |
| LGI4 | TCCTTCTCCGTGATTGAGGAC | TCTGAGGGCATTCTTAGAGATGG |
| EPYC | AGGAGGAGGAATCTACTCCCA | CAGCGGAGGAATAGCATCAAG |
| GPR21 | GTATCAGTTCTGAAGAGCGTCTC | CTCCAGGGTGTAACCAGAGTATT |
| ASB2 | GCCGTTTACTTGGCAACGTG | CTCTCGGGATTTGTTGGAGATG |
| PTH1R | CTGGGCATGATTTACACCGTG | CAGTGCAGCCGCCTAAAGTA |
| TRPC3 | AGAATGACTATCGGAAGCTCTCC | GGCAAGTTTGACACGACTTAATG |
