# Supplementary material for: Reduced APPL1 impairs osteogenic differentiation of mesenchymal stem cells by facilitating MGP expression to disrupt the BMP2 pathway in osteoporosis
Source: J Biol Chem. 2023 May 13;299(6):104823. doi: 10.1016/j.jbc.2023.104823 (PMC10318529; doi:10.1016/j.jbc.2023.104823)
Supplement: Supporting Table S2 [file mmc2.doc]

**Table S2. Characteristics of the study subjects.**

| Gene | Healthy controls | Osteoporosis patients |
| --- | --- | --- |
| Number | 10 | 10 |
| Sex | Female | Female |
| Age (year) | 59.70±7.04 | 60.30±6.77 |
| BMI (kg/m2) | 23.46±1.85 | 21.82±2.25 |
| BMD (T scope) | 1.32±1.16 | -3.57±0.89*** |
| β-crosslaps | 0.32±0.18 | 0.49±0.32 |
| PINP | 22.37±32.48 | 28.08±39.35 |
| N-MID | 31.97±15.50 | 39.27±19.85 |
