# Supplementary material for: Reduced APPL1 impairs osteogenic differentiation of mesenchymal stem cells by facilitating MGP expression to disrupt the BMP2 pathway in osteoporosis
Source: J Biol Chem. 2023 May 13;299(6):104823. doi: 10.1016/j.jbc.2023.104823 (PMC10318529; doi:10.1016/j.jbc.2023.104823)
Supplement: Supporting Table S3 [file mmc3.doc]

**Table S3. DEGs involved in the osteogenic differentiation of MSCs after APPL1 knockdown and control.**

| Gene ID | Gene Symbol | log2 (OB_Sh / NC) | Pvalue(OB_Sh / NC) | Qvalue (OB_Sh /NC) |
| --- | --- | --- | --- | --- |
| 10007 | 'GNPDA1' | 0.534904564 | 1.15E-08 | 9.51E-07 |
| 100130705 | 'ATP6V1FNB' | 1.266714415 | 0.001833859 | 0.021292303 |
| 100131211 | 'NEMP2' | -0.934784626 | 3.04E-05 | 8.15E-04 |
| 100132406 | 'NBPF10' | -0.342962244 | 0.003518082 | 0.035091222 |
| 10016 | 'PDCD6' | 0.499293564 | 1.68E-06 | 7.06E-05 |
| 10019 | 'SH2B3' | 0.588366524 | 2.47E-04 | 0.004484195 |
| 100287171 | 'WASHC1' | 0.428360509 | 1.24E-04 | 0.002597039 |
| 100288332 | 'NPIPA5' | 0.420135166 | 3.41E-04 | 0.005679402 |
| 100289635 | 'ZNF605' | 0.628747255 | 2.87E-08 | 2.21E-06 |
| 100302736 | 'TMED7-TICAM2' | -0.363042462 | 0.003688072 | 0.036363018 |
| 1004 | 'CDH6' | -1.352466756 | 6.93E-04 | 0.009982957 |
| 10046 | 'MAMLD1' | -0.856617269 | 1.55E-04 | 0.003100435 |
| 100505573 | 'INAFM2' | -0.829792119 | 3.76E-08 | 2.82E-06 |
| 100506144 | 'TMEM35B' | -0.492574545 | 1.27E-04 | 0.002654164 |
| 10051 | 'SMC4' | -0.419378236 | 0.002998917 | 0.031139639 |
| 100533467 | 'BIVM-ERCC5' | -1.081484543 | 0.003881169 | 0.037763083 |
| 10079 | 'ATP9A' | 0.547977171 | 1.48E-06 | 6.41E-05 |
| 10085 | 'EDIL3' | -1.222834528 | 9.47E-07 | 4.35E-05 |
| 10087 | 'CERT1' | -0.313924354 | 7.53E-04 | 0.010698538 |
| 10092 | 'ARPC5' | -0.876031152 | 3.17E-32 | 5.38E-29 |
| 10096 | 'ACTR3' | -0.339313182 | 5.34E-04 | 0.008140926 |
| 10098 | 'TSPAN5' | 0.560853899 | 5.91E-04 | 0.008821112 |
| 100996763 | 'NOTCH2NLB' | -0.749167077 | 6.35E-04 | 0.00933207 |
| 10106 | 'CTDSP2' | -0.29702797 | 0.002507544 | 0.027183187 |
| 101060389 | 'TBC1D3D' | 0.866833527 | 0.004598759 | 0.043068711 |
| 10109 | 'ARPC2' | -0.268054879 | 7.39E-04 | 0.010533346 |
| 10111 | 'RAD50' | 0.33256419 | 0.001516809 | 0.018246795 |
| 10123 | 'ARL4C' | -1.209787436 | 2.94E-06 | 1.16E-04 |
| 10129 | 'FRY' | -1.160957688 | 7.14E-05 | 0.001664935 |
| 10130 | 'PDIA6' | 0.795411605 | 5.87E-15 | 1.47E-12 |
| 10150 | 'MBNL2' | -0.466270284 | 5.78E-04 | 0.008683849 |
| 10159 | 'ATP6AP2' | 0.440201716 | 4.03E-05 | 0.001036825 |
| 10163 | 'WASF2' | -0.942408142 | 7.46E-11 | 9.75E-09 |
| 10165 | 'SLC25A13' | 0.37349205 | 0.002925316 | 0.030637417 |
| 10172 | 'ZNF256' | 0.788577032 | 0.004164326 | 0.039856543 |
| 10179 | 'RBM7' | 0.324568307 | 0.005490023 | 0.049611598 |
| 10180 | 'RBM6' | 0.695274881 | 3.51E-17 | 1.22E-14 |
| 1019 | 'CDK4' | -0.481931277 | 1.34E-05 | 4.15E-04 |
| 10193 | 'RNF41' | -0.350323178 | 2.25E-04 | 0.004155153 |
| 10197 | 'PSME3' | -0.466105232 | 5.55E-07 | 2.82E-05 |
| 10216 | 'PRG4' | 1.928612018 | 0.001106011 | 0.014372616 |
| 1026 | 'CDKN1A' | -1.220839305 | 4.37E-33 | 8.26E-30 |
| 10262 | 'SF3B4' | -0.271840544 | 0.003062902 | 0.031610934 |
| 102724594 | 'U2AF1L5' | 1.004200142 | 1.66E-08 | 1.33E-06 |
| 10274 | 'STAG1' | -0.443785969 | 3.03E-06 | 1.18E-04 |
| 10278 | 'EFS' | -1.011400926 | 3.10E-04 | 0.005303318 |
| 1028 | 'CDKN1C' | -1.004894348 | 3.41E-04 | 0.005679402 |
| 10285 | 'SMNDC1' | -0.342899522 | 0.00153388 | 0.018400069 |
| 10290 | 'SPEG' | -0.864294933 | 9.45E-04 | 0.012719632 |
| 10311 | 'VPS26C' | 0.477475591 | 6.99E-09 | 6.25E-07 |
| 10313 | 'RTN3' | -0.318790623 | 6.44E-04 | 0.009439068 |
| 10335 | 'IRAG1' | -1.239872423 | 3.51E-04 | 0.005811829 |
| 10336 | 'PCGF3' | 0.372675782 | 1.58E-04 | 0.00313825 |
| 10346 | 'TRIM22' | -0.567388232 | 3.96E-05 | 0.001021644 |
| 10350 | 'ABCA9' | 1.426456428 | 0.001266822 | 0.015962521 |
| 10360 | 'NPM3' | 0.761715912 | 0.001014902 | 0.013384333 |
| 10379 | 'IRF9' | -0.428956609 | 0.005273983 | 0.048119783 |
| 10389 | 'SCML2' | -1.128960581 | 0.002724967 | 0.028913228 |
| 10398 | 'MYL9' | -0.900406196 | 3.55E-04 | 0.005873005 |
| 10402 | 'ST3GAL6' | 1.055293628 | 0.002175565 | 0.024281186 |
| 10410 | 'IFITM3' | -0.392331535 | 0.001358406 | 0.016817326 |
| 10413 | 'YAP1' | -0.434776892 | 1.41E-05 | 4.35E-04 |
| 10421 | 'CD2BP2' | -0.243035039 | 0.0040556 | 0.039013632 |
| 10423 | 'CDIPT' | 0.399401802 | 2.98E-04 | 0.005171041 |
| 10427 | 'SEC24B' | -0.326383137 | 3.07E-04 | 0.00526906 |
| 10434 | 'LYPLA1' | 0.400824876 | 5.91E-05 | 0.001417817 |
| 10447 | 'FAM3C' | 0.77086301 | 6.71E-07 | 3.29E-05 |
| 10455 | 'ECI2' | 0.408533862 | 1.01E-04 | 0.002207646 |
| 10468 | 'FST' | -1.00129492 | 4.17E-10 | 4.66E-08 |
| 10487 | 'CAP1' | -0.270513986 | 0.001304453 | 0.016338123 |
| 10491 | 'CRTAP' | -0.336683751 | 5.84E-06 | 2.04E-04 |
| 10498 | 'CARM1' | 0.345778471 | 8.64E-04 | 0.011944129 |
| 10512 | 'SEMA3C' | 0.818667018 | 1.10E-06 | 4.93E-05 |
| 10513 | 'APPBP2' | -0.359665795 | 3.30E-04 | 0.005541053 |
| 10520 | 'ZNF211' | 0.496333705 | 6.60E-04 | 0.009619098 |
| 10531 | 'PITRM1' | 0.472473292 | 6.77E-04 | 0.009808467 |
| 10544 | 'PROCR' | -0.692973026 | 1.46E-04 | 0.002965324 |
| 10549 | 'PRDX4' | 0.784690606 | 8.97E-14 | 1.93E-11 |
| 10552 | 'ARPC1A' | 0.584252127 | 7.83E-14 | 1.73E-11 |
| 10554 | 'AGPAT1' | -0.702330459 | 1.83E-13 | 3.71E-11 |
| 10573 | 'MRPL28' | -0.365887803 | 2.76E-04 | 0.004876031 |
| 10600 | 'USP16' | -0.801215325 | 2.41E-17 | 8.73E-15 |
| 10611 | 'PDLIM5' | -0.706016852 | 0.001115761 | 0.014444554 |
| 10613 | 'ERLIN1' | -0.653859881 | 3.66E-10 | 4.21E-08 |
| 10618 | 'TGOLN2' | 0.248751898 | 0.001426172 | 0.01747806 |
| 10627 | 'MYL12A' | -0.421740655 | 4.92E-04 | 0.007589038 |
| 10640 | 'EXOC5' | -0.453931337 | 2.32E-05 | 6.53E-04 |
| 10644 | 'IGF2BP2' | 0.409248454 | 2.12E-05 | 6.05E-04 |
| 10678 | 'B3GNT2' | -0.562907709 | 0.001554962 | 0.018613557 |
| 10681 | 'GNB5' | -0.500082887 | 1.51E-04 | 0.003029088 |
| 1069 | 'CETN2' | 0.301139673 | 0.001091315 | 0.014225588 |
| 10712 | 'FAM189B' | 0.342006441 | 0.001370796 | 0.016921416 |
| 1072 | 'CFL1' | -0.278753827 | 0.002218545 | 0.024696026 |
| 10749 | 'KIF1C' | 0.338723249 | 8.10E-04 | 0.011327475 |
| 1075 | 'CTSC' | 1.723502469 | 1.08E-06 | 4.88E-05 |
| 10780 | 'ZNF234' | 0.404721013 | 0.003938359 | 0.038231997 |
| 10782 | 'ZNF274' | 1.107585999 | 3.53E-18 | 1.43E-15 |
| 10783 | 'NEK6' | -0.434039957 | 0.004327398 | 0.041097717 |
| 10787 | 'NCKAP1' | -0.296271637 | 5.02E-04 | 0.007725129 |
| 107984449 | 'LOC107984449' | 1.656412272 | 2.39E-05 | 6.71E-04 |
| 107984832 | 'LOC107984832' | -1.594213007 | 1.15E-04 | 0.002440878 |
| 107987457 | 'LOC107987457' | 1.032553819 | 4.66E-04 | 0.007240203 |
| 10808 | 'HSPH1' | 0.467187463 | 1.62E-05 | 4.89E-04 |
| 10809 | 'STARD10' | -0.697915956 | 0.00513789 | 0.047233016 |
| 10867 | 'TSPAN9' | -1.577254629 | 2.84E-08 | 2.19E-06 |
| 10905 | 'MAN1A2' | -0.906286321 | 4.94E-19 | 2.25E-16 |
| 10916 | 'MAGED2' | -0.440222602 | 7.72E-05 | 0.001763041 |
| 10920 | 'COPS8' | 0.410806284 | 0.003980505 | 0.038481949 |
| 10923 | 'SUB1' | -0.370107005 | 0.00215061 | 0.024113501 |
| 10929 | 'SRSF8' | 0.383001172 | 0.003258181 | 0.032907047 |
| 10933 | 'MORF4L1' | -0.264636452 | 1.12E-04 | 0.002396115 |
| 10946 | 'SF3A3' | -0.364505558 | 1.35E-04 | 0.002787806 |
| 10948 | 'STARD3' | 0.316649302 | 0.001028304 | 0.013518262 |
| 10953 | 'TOMM34' | 0.488091328 | 2.00E-04 | 0.003803873 |
| 10954 | 'PDIA5' | -1.341296393 | 1.03E-05 | 3.35E-04 |
| 10955 | 'SERINC3' | -0.602177736 | 3.24E-15 | 8.47E-13 |
| 10960 | 'LMAN2' | 0.398941802 | 4.39E-07 | 2.30E-05 |
| 10961 | 'ERP29' | 0.590067683 | 4.82E-15 | 1.24E-12 |
| 10965 | 'ACOT2' | -0.587745671 | 1.07E-05 | 3.44E-04 |
| 10979 | 'FERMT2' | -0.493853532 | 0.002081837 | 0.023497391 |
| 10988 | 'METAP2' | -0.40464313 | 1.99E-05 | 5.76E-04 |
| 10999 | 'SLC27A4' | 0.298914652 | 0.00213013 | 0.023947055 |
| 11015 | 'KDELR3' | -0.703797082 | 1.98E-07 | 1.16E-05 |
| 11018 | 'TMED1' | 0.591000915 | 4.31E-06 | 1.57E-04 |
| 11020 | 'IFT27' | 0.987784974 | 2.49E-05 | 6.93E-04 |
| 11034 | 'DSTN' | -1.159247142 | 1.59E-05 | 4.79E-04 |
| 11054 | 'OGFR' | 0.66297166 | 3.18E-07 | 1.76E-05 |
| 11082 | 'ESM1' | 2.478663914 | 3.14E-07 | 1.75E-05 |
| 11101 | 'ATE1' | 0.33444435 | 0.005255454 | 0.048028071 |
| 11102 | 'RPP14' | 0.678761546 | 1.91E-04 | 0.003686476 |
| 11112 | 'HIBADH' | 0.363601908 | 4.60E-05 | 0.001148127 |
| 11118 | 'BTN3A2' | 0.646121869 | 3.00E-04 | 0.005192334 |
| 11138 | 'TBC1D8' | 1.231003954 | 1.15E-07 | 7.24E-06 |
| 11142 | 'PKIG' | 0.627734158 | 2.09E-04 | 0.003931965 |
| 11155 | 'LDB3' | -2.33725136 | 3.06E-04 | 0.005263401 |
| 11159 | 'RABL2A' | 0.566047827 | 0.005298245 | 0.048263435 |
| 11160 | 'ERLIN2' | -0.538100289 | 7.98E-07 | 3.76E-05 |
| 11164 | 'NUDT5' | 0.389875086 | 8.98E-05 | 0.00200247 |
| 11176 | 'BAZ2A' | 0.23177384 | 0.002747773 | 0.029100718 |
| 11190 | 'CEP250' | 0.342206557 | 0.004001096 | 0.038576652 |
| 112 | 'ADCY6' | -0.621074888 | 3.15E-07 | 1.75E-05 |
| 11218 | 'DDX20' | -0.656423702 | 1.68E-07 | 1.01E-05 |
| 1122 | 'CHML' | -0.89291346 | 3.27E-04 | 0.005503533 |
| 1123 | 'CHN1' | -0.521040415 | 0.002561173 | 0.027553679 |
| 11234 | 'HPS5' | -0.619037661 | 5.69E-05 | 0.001370504 |
| 112464 | 'CAVIN3' | -0.915854174 | 7.87E-12 | 1.21E-09 |
| 112487 | 'DTD2' | -0.54172358 | 0.00312899 | 0.032078754 |
| 11252 | 'PACSIN2' | -0.600384222 | 6.60E-05 | 0.001556018 |
| 112574 | 'SNX18' | -0.556604133 | 1.64E-08 | 1.33E-06 |
| 11260 | 'XPOT' | 0.470140419 | 4.03E-04 | 0.006523413 |
| 112611 | 'RWDD2A' | 0.478881777 | 0.004376848 | 0.041516556 |
| 11267 | 'SNF8' | -0.356562381 | 6.58E-05 | 0.001552848 |
| 11269 | 'DDX19B' | 0.335400911 | 0.004711637 | 0.044028812 |
| 11277 | 'TREX1' | -0.90889372 | 0.004327857 | 0.041097717 |
| 112858 | 'TP53RK' | -0.332090796 | 0.001940695 | 0.022289145 |
| 112942 | 'CFAP36' | 0.415444875 | 2.21E-04 | 0.004107021 |
| 113000 | 'RPUSD1' | 0.424340724 | 0.003573053 | 0.035517396 |
| 113235 | 'SLC46A1' | -0.486160017 | 0.00334925 | 0.033766641 |
| 113277 | 'TMEM106A' | -0.895570023 | 9.55E-05 | 0.002107525 |
| 11337 | 'GABARAP' | -0.237837279 | 0.001720067 | 0.020191786 |
| 114 | 'ADCY8' | 3.030903646 | 1.54E-06 | 6.57E-05 |
| 114795 | 'TMEM132B' | 1.745855473 | 5.79E-04 | 0.008685966 |
| 114818 | 'KLHL29' | -0.679958371 | 7.09E-04 | 0.010171767 |
| 114881 | 'OSBPL7' | -1.058990259 | 0.001657409 | 0.019616074 |
| 114902 | 'C1QTNF5' | -1.104383094 | 1.72E-07 | 1.03E-05 |
| 114907 | 'FBXO32' | 0.572850876 | 3.55E-09 | 3.26E-07 |
| 115207 | 'KCTD12' | -0.762278188 | 0.001246841 | 0.015807744 |
| 115353 | 'LRRC42' | -0.42880598 | 6.58E-04 | 0.009599958 |
| 115557 | 'ARHGEF25' | -0.629483235 | 3.01E-06 | 1.18E-04 |
| 115572 | 'TENT5B' | -1.019092594 | 0.003396616 | 0.03412274 |
| 115701 | 'ALPK2' | -1.089372821 | 0.001473515 | 0.017903371 |
| 115817 | 'DHRS1' | -0.697004351 | 5.22E-04 | 0.007991544 |
| 115825 | 'WDFY2' | 0.317283045 | 0.002024134 | 0.022998812 |
| 116113 | 'FOXP4' | 0.621656535 | 0.001310154 | 0.016374999 |
| 116151 | 'FAM210B' | 0.666385991 | 4.29E-11 | 5.78E-09 |
| 116224 | 'FAM122A' | -0.452512228 | 0.002036255 | 0.023120345 |
| 1173 | 'AP2M1' | -0.253378739 | 1.21E-04 | 0.002544848 |
| 117583 | 'PARD3B' | -0.490814962 | 1.16E-06 | 5.19E-05 |
| 1176 | 'AP3S1' | 0.359147444 | 2.86E-05 | 7.79E-04 |
| 118987 | 'PDZD8' | -0.433017725 | 5.74E-04 | 0.008633398 |
| 119032 | 'BORCS7' | -0.534364311 | 0.002710227 | 0.02879277 |
| 1191 | 'CLU' | 1.30143952 | 6.79E-10 | 7.22E-08 |
| 12 | 'SERPINA3' | 1.639162632 | 0.001977923 | 0.022579408 |
| 1200 | 'TPP1' | -0.591025784 | 1.02E-11 | 1.54E-09 |
| 1203 | 'CLN5' | -0.307331153 | 5.43E-04 | 0.008247888 |
| 1207 | 'CLNS1A' | -0.55892479 | 2.90E-07 | 1.62E-05 |
| 1211 | 'CLTA' | -0.27015987 | 1.00E-04 | 0.002197037 |
| 122786 | 'FRMD6' | -0.68870908 | 0.001442801 | 0.017593065 |
| 122953 | 'JDP2' | -0.657666694 | 2.21E-04 | 0.004107021 |
| 123016 | 'TTC8' | 0.373383519 | 0.002322577 | 0.025699021 |
| 123263 | 'MTFMT' | 0.520906857 | 4.27E-04 | 0.006776458 |
| 123283 | 'TARS3' | 0.79749991 | 3.41E-05 | 9.00E-04 |
| 123811 | 'CEP20' | 0.550546165 | 5.49E-06 | 1.94E-04 |
| 124245 | 'ZC3H18' | 0.358670416 | 2.81E-04 | 0.004958778 |
| 124454 | 'EARS2' | 0.547173867 | 0.001165042 | 0.014945947 |
| 125 | 'ADH1B' | 3.483657574 | 9.74E-12 | 1.48E-09 |
| 125111 | 'GJD3' | 0.577527403 | 0.001485934 | 0.017989965 |
| 126 | 'ADH1C' | 2.421632422 | 0.002495697 | 0.027106622 |
| 126070 | 'ZNF440' | 0.580199916 | 7.25E-05 | 0.001687355 |
| 1264 | 'CNN1' | -1.378105102 | 0.003721663 | 0.036562859 |
| 1265 | 'CNN2' | -0.685771534 | 3.69E-13 | 7.12E-11 |
| 1266 | 'CNN3' | -0.522221518 | 9.62E-04 | 0.012908046 |
| 1267 | 'CNP' | -0.507593689 | 6.20E-06 | 2.14E-04 |
| 126731 | 'CCSAP' | -0.81346814 | 7.87E-07 | 3.72E-05 |
| 126969 | 'SLC44A3' | -1.94641088 | 0.001456637 | 0.017723771 |
| 127018 | 'LYPLAL1' | 0.659250416 | 1.46E-06 | 6.34E-05 |
| 128240 | 'NAXE' | -0.374199608 | 6.85E-04 | 0.009895151 |
| 128272 | 'ARHGEF19' | -0.753885994 | 0.003975533 | 0.038461513 |
| 1284 | 'COL4A2' | -0.497461076 | 8.64E-04 | 0.011944129 |
| 1287 | 'COL4A5' | -1.627312751 | 1.07E-09 | 1.11E-07 |
| 128710 | 'SLX4IP' | 0.743856824 | 0.002951585 | 0.030817596 |
| 128989 | 'TANGO2' | -0.363242703 | 0.003098297 | 0.031840899 |
| 1293 | 'COL6A3' | 0.527803981 | 0.004543658 | 0.042717426 |
| 1295 | 'COL8A1' | -0.397088871 | 0.005353399 | 0.048517682 |
| 1301 | 'COL11A1' | -1.416963866 | 2.54E-05 | 7.03E-04 |
| 1303 | 'COL12A1' | -0.834775258 | 5.13E-05 | 0.001252083 |
| 130497 | 'OSR1' | 1.186221852 | 1.87E-07 | 1.10E-05 |
| 130733 | 'TMEM178A' | -1.325579503 | 3.18E-04 | 0.005390179 |
| 130814 | 'SLC66A3' | -0.917029308 | 6.63E-10 | 7.09E-08 |
| 131544 | 'CRYBG3' | -0.558346976 | 0.001357597 | 0.016817326 |
| 131566 | 'DCBLD2' | 0.718559403 | 0.001768134 | 0.020684615 |
| 131578 | 'LRRC15' | -1.5574138 | 1.81E-06 | 7.57E-05 |
| 1317 | 'SLC31A1' | -0.622732257 | 3.32E-09 | 3.08E-07 |
| 132160 | 'PPM1M' | -0.712112711 | 9.11E-05 | 0.002023714 |
| 132864 | 'CPEB2' | 0.45303479 | 6.17E-04 | 0.009082007 |
| 1329 | 'COX5B' | -0.306026163 | 2.26E-04 | 0.004174548 |
| 133686 | 'NADK2' | -0.938031249 | 2.27E-10 | 2.69E-08 |
| 1340 | 'COX6B1' | -0.300088057 | 8.02E-04 | 0.01129084 |
| 134430 | 'WDR36' | 0.404810263 | 0.004867937 | 0.045240672 |
| 134553 | 'C5orf24' | -0.655519327 | 3.73E-20 | 1.98E-17 |
| 1356 | 'CP' | 2.235821757 | 6.09E-07 | 3.03E-05 |
| 1362 | 'CPD' | -0.401834308 | 0.002980017 | 0.031057219 |
| 136319 | 'MTPN' | -0.526779469 | 3.68E-06 | 1.38E-04 |
| 136647 | 'MPLKIP' | -0.60917036 | 4.15E-05 | 0.001056379 |
| 1370 | 'CPN2' | -1.09917876 | 2.86E-04 | 0.005013536 |
| 137886 | 'UBXN2B' | 0.418675823 | 0.002539326 | 0.027405377 |
| 138050 | 'HGSNAT' | 0.226642372 | 0.001489781 | 0.018023696 |
| 138151 | 'NACC2' | -0.817442657 | 9.64E-08 | 6.32E-06 |
| 138199 | 'CARNMT1' | 0.514627531 | 1.10E-04 | 0.002357494 |
| 1384 | 'CRAT' | 0.373452071 | 1.49E-04 | 0.002999617 |
| 1386 | 'ATF2' | -0.527530501 | 3.78E-09 | 3.46E-07 |
| 1388 | 'ATF6B' | -0.484342946 | 8.11E-05 | 0.00184068 |
| 1389 | 'CREBL2' | 0.42833466 | 6.05E-04 | 0.008959797 |
| 140688 | 'NOL4L' | -0.790490764 | 9.00E-05 | 0.002004736 |
| 140775 | 'SMCR8' | 0.363946473 | 1.27E-04 | 0.002648262 |
| 140886 | 'PABPC5' | -1.327858079 | 4.97E-05 | 0.001223764 |
| 142891 | 'SAMD8' | -0.408560194 | 3.39E-05 | 8.96E-04 |
| 143187 | 'VTI1A' | -0.939253467 | 9.95E-08 | 6.50E-06 |
| 1434 | 'CSE1L' | 0.642219807 | 7.55E-12 | 1.18E-09 |
| 143458 | 'LDLRAD3' | 0.715427198 | 1.07E-04 | 0.002308692 |
| 143903 | 'LAYN' | -0.509034576 | 1.63E-04 | 0.003234724 |
| 144348 | 'ZNF664' | 0.637651117 | 4.88E-07 | 2.52E-05 |
| 144717 | 'PHETA1' | 0.835854759 | 5.76E-06 | 2.02E-04 |
| 1453 | 'CSNK1D' | -0.368708681 | 1.34E-04 | 0.002769886 |
| 145567 | 'TTC7B' | -0.486441413 | 0.00279464 | 0.029523492 |
| 145864 | 'HAPLN3' | -1.436584318 | 2.00E-04 | 0.003810328 |
| 146050 | 'ZSCAN29' | 0.670910209 | 2.40E-07 | 1.37E-05 |
| 146198 | 'ZFP90' | 0.547448903 | 6.94E-04 | 0.009982957 |
| 1462 | 'VCAN' | -0.843825151 | 2.69E-05 | 7.41E-04 |
| 146223 | 'CMTM4' | -0.508542778 | 9.00E-04 | 0.012322825 |
| 1464 | 'CSPG4' | -1.038004266 | 6.81E-05 | 0.001593583 |
| 1465 | 'CSRP1' | -0.838257048 | 1.70E-05 | 5.04E-04 |
| 1466 | 'CSRP2' | -1.5830984 | 7.64E-05 | 0.00175305 |
| 146691 | 'TOM1L2' | -0.557500863 | 2.65E-06 | 1.06E-04 |
| 146705 | 'TEPSIN' | 0.591023609 | 4.56E-04 | 0.007127735 |
| 147 | 'ADRA1B' | 1.024343533 | 0.002506889 | 0.027183187 |
| 147040 | 'KCTD11' | -0.52872273 | 1.94E-05 | 5.63E-04 |
| 147657 | 'ZNF480' | 0.400242623 | 0.002516739 | 0.027230761 |
| 147660 | 'ZNF578' | 0.951381086 | 2.97E-04 | 0.005156355 |
| 147741 | 'ZNF560' | 2.219678379 | 7.06E-05 | 0.001648764 |
| 147906 | 'DACT3' | -1.213888767 | 1.04E-04 | 0.002246469 |
| 148156 | 'ZNF558' | 0.426010178 | 0.004206105 | 0.040205932 |
| 148170 | 'CDC42EP5' | -0.988299907 | 0.001189555 | 0.01519163 |
| 148252 | 'DIRAS1' | -1.095986443 | 2.44E-06 | 9.83E-05 |
| 148268 | 'ZNF570' | 0.603829646 | 0.00201179 | 0.022904493 |
| 148281 | 'SYT6' | -3.092405638 | 0.00113216 | 0.014608752 |
| 148867 | 'SLC30A7' | 0.338706303 | 0.001951244 | 0.022364968 |
| 149041 | 'RC3H1' | 0.560032215 | 4.07E-04 | 0.006558483 |
| 149111 | 'CNIH3' | 1.226161837 | 4.64E-05 | 0.00115334 |
| 1500 | 'CTNND1' | -0.487527143 | 1.92E-04 | 0.003700751 |
| 1503 | 'CTPS1' | -0.553392192 | 9.06E-04 | 0.012368653 |
| 150946 | 'GAREM2' | -0.467587441 | 0.004381356 | 0.041535446 |
| 151011 | 'SEPTIN10' | -0.643024054 | 1.05E-07 | 6.76E-06 |
| 1514 | 'CTSL' | 1.004378331 | 7.52E-07 | 3.59E-05 |
| 151636 | 'DTX3L' | -0.395481492 | 0.003057958 | 0.031595 |
| 1519 | 'CTSO' | 0.430943305 | 1.66E-07 | 1.00E-05 |
| 1522 | 'CTSZ' | 0.328632867 | 0.003766287 | 0.036856272 |
| 152217 | 'NCBP2AS2' | 0.775544848 | 0.001308939 | 0.016371854 |
| 153090 | 'DAB2IP' | -1.141747654 | 1.22E-04 | 0.002567463 |
| 1543 | 'CYP1A1' | 1.980360658 | 9.41E-04 | 0.01267032 |
| 1545 | 'CYP1B1' | 0.647166903 | 6.87E-15 | 1.69E-12 |
| 154743 | 'BMT2' | -0.796915438 | 3.38E-06 | 1.29E-04 |
| 154810 | 'AMOTL1' | 0.359961079 | 5.93E-04 | 0.008828925 |
| 157285 | 'PRAG1' | -1.202013626 | 1.22E-07 | 7.67E-06 |
| 157567 | 'ANKRD46' | 0.625488364 | 7.40E-04 | 0.010544764 |
| 158399 | 'ZNF483' | 1.200443497 | 1.69E-04 | 0.003323738 |
| 158747 | 'MOSPD2' | -0.479947812 | 4.50E-04 | 0.007036712 |
| 159 | 'ADSS2' | 0.595489909 | 1.61E-11 | 2.38E-09 |
| 159013 | 'CXorf38' | -0.464900472 | 3.45E-04 | 0.005727873 |
| 16 | 'AARS1' | 0.567145687 | 1.76E-07 | 1.04E-05 |
| 160418 | 'TMTC3' | -0.67674568 | 6.08E-10 | 6.54E-08 |
| 1620 | 'BRINP1' | 1.436329045 | 2.90E-06 | 1.14E-04 |
| 162073 | 'ITPRIPL2' | 0.294983547 | 1.05E-04 | 0.002264515 |
| 162967 | 'ZNF320' | 0.518613714 | 1.55E-04 | 0.003095498 |
| 163081 | 'ZNF567' | 0.699883743 | 7.97E-04 | 0.011245877 |
| 163175 | 'LGI4' | -2.274273954 | 0.00141237 | 0.017343542 |
| 1645 | 'AKR1C1' | 1.57866204 | 1.98E-05 | 5.74E-04 |
| 1646 | 'AKR1C2' | 1.500622124 | 3.10E-04 | 0.005303318 |
| 1647 | 'GADD45A' | -0.747472381 | 9.73E-05 | 0.00214325 |
| 1650 | 'DDOST' | 0.320352432 | 1.63E-04 | 0.003234724 |
| 1652 | 'DDT' | -0.965458422 | 2.13E-22 | 1.39E-19 |
| 1656 | 'DDX6' | 0.265641856 | 4.42E-04 | 0.006952037 |
| 165918 | 'RNF168' | 0.479076173 | 4.20E-04 | 0.00670457 |
| 167838 | 'TXLNB' | 3.170765627 | 1.35E-07 | 8.39E-06 |
| 169200 | 'TMEM64' | -0.684767638 | 0.001194631 | 0.015244995 |
| 170685 | 'NUDT10' | -1.79376244 | 0.002775251 | 0.029355147 |
| 170689 | 'ADAMTS15' | 1.599469673 | 0.001878304 | 0.02174892 |
| 170691 | 'ADAMTS17' | 2.14568026 | 0.005117304 | 0.047120224 |
| 170960 | 'ZNF721' | 0.650263498 | 4.47E-06 | 1.62E-04 |
| 171023 | 'ASXL1' | 0.297192988 | 0.001430947 | 0.017523941 |
| 171024 | 'SYNPO2' | -2.143834063 | 6.66E-07 | 3.27E-05 |
| 1728 | 'NQO1' | -0.774256721 | 1.11E-05 | 3.53E-04 |
| 1738 | 'DLD' | 0.325601883 | 0.001844572 | 0.021387468 |
| 1741 | 'DLG3' | -0.838066593 | 0.001694955 | 0.019979777 |
| 175 | 'AGA' | 0.486491038 | 0.001337839 | 0.016659775 |
| 176 | 'ACAN' | -1.838435547 | 3.43E-09 | 3.17E-07 |
| 1760 | 'DMPK' | -0.681897318 | 1.05E-04 | 0.002257682 |
| 18 | 'ABAT' | -1.522639686 | 1.98E-08 | 1.56E-06 |
| 1800 | 'DPEP1' | -1.915293814 | 8.99E-04 | 0.012319934 |
| 1803 | 'DPP4' | 1.448146856 | 7.13E-04 | 0.010213834 |
| 1808 | 'DPYSL2' | 0.257610719 | 0.001973618 | 0.022545402 |
| 1833 | 'EPYC' | -2.95242506 | 0.001258493 | 0.015904736 |
| 1836 | 'SLC26A2' | -0.684971644 | 1.14E-07 | 7.20E-06 |
| 1837 | 'DTNA' | 0.743005505 | 0.001498737 | 0.018106283 |
| 1848 | 'DUSP6' | 1.19816893 | 3.85E-06 | 1.43E-04 |
| 1856 | 'DVL2' | -0.534987162 | 3.32E-04 | 0.00556571 |
| 1889 | 'ECE1' | 0.424337893 | 1.13E-04 | 0.002406161 |
| 1891 | 'ECH1' | 0.590376549 | 1.43E-04 | 0.002912471 |
| 1893 | 'ECM1' | -0.877621819 | 5.20E-15 | 1.32E-12 |
| 1903 | 'S1PR3' | 1.180127968 | 5.32E-16 | 1.51E-13 |
| 1910 | 'EDNRB' | -1.877933928 | 3.21E-09 | 3.02E-07 |
| 1911 | 'PHC1' | -0.385736648 | 0.003316409 | 0.03345538 |
| 192670 | 'AGO4' | 0.429656467 | 0.002171251 | 0.024278603 |
| 1956 | 'EGFR' | -0.707103474 | 1.92E-04 | 0.003700751 |
| 196441 | 'ZFC3H1' | -0.296826353 | 0.001564852 | 0.018718753 |
| 196740 | 'VSTM4' | -0.466822985 | 0.001424631 | 0.017471776 |
| 1983 | 'EIF5' | 0.309151609 | 8.27E-05 | 0.001866741 |
| 2000 | 'ELF4' | -0.729477854 | 7.50E-08 | 5.02E-06 |
| 200316 | 'APOBEC3F' | -0.997305545 | 2.43E-04 | 0.004428324 |
| 2004 | 'ELK3' | 0.682559551 | 5.83E-04 | 0.008733946 |
| 2005 | 'ELK4' | 0.530606061 | 6.76E-04 | 0.009802045 |
| 200765 | 'TIGD1' | 1.128326289 | 1.50E-05 | 4.56E-04 |
| 2009 | 'EML1' | 0.511726919 | 2.32E-05 | 6.53E-04 |
| 2012 | 'EMP1' | 1.028605548 | 8.82E-05 | 0.001977492 |
| 201475 | 'RAB12' | -0.959059123 | 2.50E-15 | 6.75E-13 |
| 201562 | 'HACD2' | -1.365724698 | 2.43E-10 | 2.85E-08 |
| 201595 | 'STT3B' | -0.742097965 | 2.55E-12 | 4.33E-10 |
| 201626 | 'PDE12' | 0.291873978 | 0.003032301 | 0.031390407 |
| 2017 | 'CTTN' | 0.30163753 | 1.21E-05 | 3.80E-04 |
| 2022 | 'ENG' | 0.848817233 | 1.20E-05 | 3.77E-04 |
| 2033 | 'EP300' | -0.309924405 | 1.68E-05 | 4.99E-04 |
| 2049 | 'EPHB3' | -1.323279174 | 1.07E-05 | 3.45E-04 |
| 2057 | 'EPOR' | -1.002797737 | 0.004765929 | 0.044438433 |
| 2059 | 'EPS8' | -0.783203795 | 2.18E-13 | 4.36E-11 |
| 206338 | 'LVRN' | 2.672868581 | 1.09E-05 | 3.47E-04 |
| 206358 | 'SLC36A1' | -0.513316013 | 6.10E-06 | 2.11E-04 |
| 2069 | 'EREG' | 2.407990167 | 0.00124213 | 0.015768285 |
| 2115 | 'ETV1' | 0.769032999 | 6.07E-04 | 0.008983648 |
| 2118 | 'ETV4' | 1.092066864 | 0.003527955 | 0.035151338 |
| 2167 | 'FABP4' | 2.28971354 | 0.001601969 | 0.019068812 |
| 2171 | 'FABP5' | 0.905256192 | 1.56E-05 | 4.73E-04 |
| 219 | 'ALDH1B1' | -1.043505002 | 8.30E-06 | 2.79E-04 |
| 2191 | 'FAP' | 0.365300075 | 6.45E-04 | 0.009448594 |
| 219333 | 'USP12' | -0.881487343 | 4.17E-11 | 5.67E-09 |
| 219899 | 'TBCEL' | -0.411208783 | 3.85E-05 | 0.001000413 |
| 219988 | 'PATL1' | -0.492792904 | 1.43E-05 | 4.40E-04 |
| 220108 | 'FAM124A' | -1.217161133 | 1.47E-04 | 0.002972232 |
| 221 | 'ALDH3B1' | 0.36223374 | 0.002927996 | 0.030646603 |
| 221061 | 'FAM171A1' | 0.43836284 | 2.92E-04 | 0.005074064 |
| 221656 | 'KDM1B' | -0.424465123 | 4.27E-04 | 0.006776458 |
| 221710 | 'SMIM13' | -1.20416401 | 3.92E-07 | 2.11E-05 |
| 2218 | 'FKTN' | -0.491661013 | 9.39E-06 | 3.11E-04 |
| 221830 | 'POLR1F' | -0.365535062 | 8.11E-04 | 0.011337969 |
| 221895 | 'JAZF1' | -0.63272887 | 7.23E-04 | 0.010327384 |
| 221955 | 'DAGLB' | 0.397607769 | 0.003435033 | 0.034405275 |
| 222658 | 'KCTD20' | -1.1142561 | 5.87E-18 | 2.27E-15 |
| 222698 | 'NKAPL' | 1.945172241 | 1.23E-07 | 7.70E-06 |
| 2235 | 'FECH' | -0.809553027 | 1.64E-05 | 4.93E-04 |
| 2239 | 'GPC4' | -1.138792462 | 0.002393511 | 0.026265273 |
| 2241 | 'FER' | -0.706796288 | 4.93E-07 | 2.53E-05 |
| 2246 | 'FGF1' | -1.072326412 | 4.57E-04 | 0.007127735 |
| 2252 | 'FGF7' | -3.525449052 | 1.73E-07 | 1.03E-05 |
| 22795 | 'NID2' | -0.656217335 | 0.003714537 | 0.03656034 |
| 22801 | 'ITGA11' | -0.514611926 | 2.60E-04 | 0.004648832 |
| 22826 | 'DNAJC8' | -0.222269882 | 0.0049926 | 0.046222343 |
| 22834 | 'ZNF652' | 0.790413397 | 7.48E-09 | 6.55E-07 |
| 22836 | 'RHOBTB3' | -0.57294603 | 0.002075173 | 0.023468924 |
| 22837 | 'COBLL1' | -0.979929989 | 4.40E-05 | 0.001108319 |
| 22838 | 'RNF44' | 0.671927665 | 9.09E-06 | 3.03E-04 |
| 22841 | 'RAB11FIP2' | -0.886361843 | 6.80E-05 | 0.001593583 |
| 22856 | 'CHSY1' | -0.422837332 | 0.001179361 | 0.015084104 |
| 22858 | 'CILK1' | -0.468091826 | 0.005367171 | 0.04860478 |
| 22864 | 'R3HDM2' | -0.418814332 | 2.93E-05 | 7.92E-04 |
| 22869 | 'ZNF510' | 0.39750196 | 0.0027269 | 0.028915683 |
| 22870 | 'PPP6R1' | 0.500071068 | 1.16E-05 | 3.66E-04 |
| 22872 | 'SEC31A' | -0.220597727 | 0.005317787 | 0.048311996 |
| 22877 | 'MLXIP' | 0.521281184 | 2.22E-05 | 6.30E-04 |
| 2288 | 'FKBP4' | 0.705300256 | 1.36E-04 | 0.002793194 |
| 22880 | 'MORC2' | -0.386153983 | 5.26E-05 | 0.001279334 |
| 22881 | 'ANKRD6' | -0.972010759 | 1.78E-05 | 5.24E-04 |
| 22883 | 'CLSTN1' | -0.55268004 | 9.61E-06 | 3.17E-04 |
| 22884 | 'WDR37' | 0.990771879 | 6.09E-17 | 1.99E-14 |
| 22885 | 'ABLIM3' | 1.460408465 | 6.95E-06 | 2.38E-04 |
| 22903 | 'BTBD3' | -0.844638003 | 2.63E-09 | 2.53E-07 |
| 22916 | 'NCBP2' | -0.764224111 | 5.03E-19 | 2.25E-16 |
| 22919 | 'MAPRE1' | -0.412632719 | 4.78E-04 | 0.007407717 |
| 22927 | 'HABP4' | 0.812436031 | 8.15E-08 | 5.42E-06 |
| 22938 | 'SNW1' | -1.767982404 | 2.19E-39 | 7.43E-36 |
| 22943 | 'DKK1' | 0.657230836 | 0.00251849 | 0.02723238 |
| 22984 | 'PDCD11' | 0.311942063 | 0.005133976 | 0.047233016 |
| 22992 | 'KDM2A' | 0.543134684 | 4.87E-11 | 6.42E-09 |
| 22998 | 'LIMCH1' | -1.691206704 | 4.53E-12 | 7.56E-10 |
| 23001 | 'WDFY3' | -0.345688985 | 1.02E-04 | 0.002213147 |
| 23005 | 'MAPKBP1' | -0.377719323 | 0.00285079 | 0.030060005 |
| 23012 | 'STK38L' | -0.553978507 | 8.92E-05 | 0.001993356 |
| 23022 | 'PALLD' | -1.235690941 | 1.28E-37 | 2.72E-34 |
| 23023 | 'TMCC1' | -0.455896104 | 3.34E-04 | 0.005586 |
| 23057 | 'NMNAT2' | 0.846135143 | 2.15E-07 | 1.24E-05 |
| 23061 | 'TBC1D9B' | 0.322222763 | 4.79E-06 | 1.72E-04 |
| 23066 | 'CAND2' | -1.444996606 | 2.96E-04 | 0.005134646 |
| 23089 | 'PEG10' | -1.488124582 | 3.30E-06 | 1.26E-04 |
| 2309 | 'FOXO3' | -0.408248213 | 0.004320964 | 0.04108683 |
| 23102 | 'TBC1D2B' | -0.363873297 | 0.002676294 | 0.028483214 |
| 23111 | 'SPART' | -0.319119568 | 9.24E-04 | 0.012559786 |
| 23113 | 'CUL9' | 0.437730696 | 5.58E-04 | 0.008422341 |
| 23114 | 'NFASC' | -0.670255604 | 0.001360677 | 0.016833177 |
| 23118 | 'TAB2' | -0.62181124 | 2.53E-09 | 2.44E-07 |
| 23130 | 'ATG2A' | 0.646544795 | 1.10E-05 | 3.50E-04 |
| 23132 | 'RAD54L2' | 0.5790839 | 0.00230564 | 0.025548413 |
| 2314 | 'FLII' | 0.31418992 | 1.97E-04 | 0.003765197 |
| 23154 | 'NCDN' | -0.353786667 | 0.002492911 | 0.027093676 |
| 2316 | 'FLNA' | -0.627201457 | 2.63E-10 | 3.06E-08 |
| 23176 | 'SEPTIN8' | -0.473362061 | 0.001363734 | 0.016858724 |
| 23184 | 'MESD' | 1.314692597 | 2.94E-38 | 7.14E-35 |
| 23193 | 'GANAB' | 0.706802706 | 3.02E-15 | 8.03E-13 |
| 23204 | 'ARL6IP1' | 0.451349137 | 5.37E-08 | 3.79E-06 |
| 23208 | 'SYT11' | -0.793194189 | 1.92E-05 | 5.59E-04 |
| 23221 | 'RHOBTB2' | -0.502260531 | 2.93E-05 | 7.92E-04 |
| 23223 | 'RRP12' | 0.559333763 | 3.16E-04 | 0.005379497 |
| 23232 | 'TBC1D12' | -1.032034105 | 6.11E-07 | 3.03E-05 |
| 23236 | 'PLCB1' | -0.844773839 | 0.001793194 | 0.020905843 |
| 23252 | 'OTUD3' | 0.544503754 | 0.001781432 | 0.020782966 |
| 23268 | 'DNMBP' | 0.347411173 | 0.002551691 | 0.027486467 |
| 23271 | 'CAMSAP2' | -0.553783898 | 6.11E-08 | 4.20E-06 |
| 23288 | 'IQCE' | -0.509637453 | 1.33E-04 | 0.002759658 |
| 23291 | 'FBXW11' | -1.191038134 | 1.56E-27 | 1.76E-24 |
| 23299 | 'BICD2' | 0.642562598 | 4.63E-11 | 6.15E-09 |
| 23300 | 'ATMIN' | -0.409637782 | 3.08E-05 | 8.21E-04 |
| 23301 | 'EHBP1' | -0.751550631 | 0.002390993 | 0.026254581 |
| 23325 | 'WASHC4' | -0.489273822 | 2.88E-07 | 1.62E-05 |
| 23327 | 'NEDD4L' | 0.902921451 | 2.23E-05 | 6.31E-04 |
| 23328 | 'SASH1' | 0.616059137 | 2.47E-04 | 0.004484195 |
| 23333 | 'DPY19L1' | 0.393199129 | 4.20E-04 | 0.00670457 |
| 23336 | 'SYNM' | -0.735023723 | 9.07E-04 | 0.012368749 |
| 23361 | 'ZNF629' | 0.37594314 | 9.16E-04 | 0.012463339 |
| 23363 | 'OBSL1' | -0.594328519 | 4.06E-05 | 0.001041916 |
| 23381 | 'SMG5' | -0.253217492 | 0.003656884 | 0.036097399 |
| 23386 | 'NUDCD3' | 0.449182495 | 4.94E-06 | 1.76E-04 |
| 23400 | 'ATP13A2' | 0.497219796 | 4.14E-04 | 0.006639063 |
| 23401 | 'FRAT2' | 1.588608035 | 7.59E-12 | 1.18E-09 |
| 23405 | 'DICER1' | -0.353959231 | 2.90E-04 | 0.005069194 |
| 23423 | 'TMED3' | 0.52857307 | 8.25E-04 | 0.011469567 |
| 23432 | 'GPR161' | -0.625392191 | 5.67E-04 | 0.008546316 |
| 23467 | 'NPTXR' | -1.232054175 | 3.52E-11 | 4.86E-09 |
| 23471 | 'TRAM1' | -0.511716574 | 6.02E-04 | 0.008943505 |
| 23478 | 'SEC11A' | 0.28136068 | 1.37E-04 | 0.002820629 |
| 23492 | 'CBX7' | 0.665769581 | 1.01E-04 | 0.002202295 |
| 23523 | 'CABIN1' | 0.455532366 | 7.75E-05 | 0.001767903 |
| 23531 | 'MMD' | 0.607831176 | 3.06E-06 | 1.18E-04 |
| 23536 | 'ADAT1' | -0.67768877 | 1.68E-08 | 1.35E-06 |
| 2355 | 'FOSL2' | -0.611437009 | 5.13E-06 | 1.82E-04 |
| 23568 | 'ARL2BP' | 0.377636923 | 2.10E-06 | 8.54E-05 |
| 23576 | 'DDAH1' | -0.597992571 | 2.24E-04 | 0.004154912 |
| 23582 | 'CCNDBP1' | -0.976128062 | 6.75E-19 | 2.94E-16 |
| 23583 | 'SMUG1' | -0.683307981 | 3.36E-04 | 0.005618515 |
| 23604 | 'DAPK2' | 1.525418195 | 3.44E-06 | 1.31E-04 |
| 23670 | 'CEMIP2' | -0.803161487 | 1.11E-08 | 9.29E-07 |
| 23673 | 'STX12' | 0.440283222 | 3.93E-05 | 0.001017089 |
| 23683 | 'PRKD3' | -0.266977942 | 0.00215794 | 0.024179733 |
| 23704 | 'KCNE4' | -1.154363917 | 1.06E-07 | 6.77E-06 |
| 24138 | 'IFIT5' | -0.857657692 | 5.86E-06 | 2.04E-04 |
| 246330 | 'PELI3' | -0.938733577 | 1.47E-07 | 9.02E-06 |
| 2519 | 'FUCA2' | 0.522918265 | 2.63E-08 | 2.04E-06 |
| 253143 | 'PRR14L' | 0.480317445 | 3.54E-06 | 1.34E-04 |
| 253512 | 'SLC25A30' | -0.475033971 | 8.92E-04 | 0.012245815 |
| 2537 | 'IFI6' | -1.525312603 | 1.18E-04 | 0.002499611 |
| 253832 | 'ZDHHC20' | -0.971505974 | 1.76E-16 | 5.34E-14 |
| 254048 | 'UBN2' | 0.478993504 | 9.08E-04 | 0.012369632 |
| 254102 | 'EHBP1L1' | -0.382413404 | 0.003770645 | 0.036877687 |
| 254170 | 'FBXO33' | 0.444807683 | 2.21E-04 | 0.004110818 |
| 254228 | 'CALHM5' | -1.528442779 | 1.58E-40 | 6.71E-37 |
| 254295 | 'PHYHD1' | 0.898693021 | 0.002018946 | 0.022955211 |
| 254863 | 'TMEM256' | -0.60892589 | 0.002545721 | 0.027456955 |
| 256302 | 'NATD1' | -0.732910053 | 2.88E-07 | 1.62E-05 |
| 256691 | 'MAMDC2' | 1.710140829 | 0.001107113 | 0.014372616 |
| 256987 | 'SERINC5' | -0.622877644 | 0.004985094 | 0.046177998 |
| 25758 | 'KIAA1549L' | 0.467890757 | 0.001925035 | 0.022124239 |
| 25771 | 'TBC1D22A' | -0.604819122 | 2.26E-06 | 9.16E-05 |
| 25789 | 'TMEM59L' | -1.457860732 | 7.79E-05 | 0.001775847 |
| 25794 | 'FSCN2' | -2.09618804 | 2.73E-04 | 0.004840615 |
| 2580 | 'GAK' | 0.453995218 | 6.61E-05 | 0.001556145 |
| 25802 | 'LMOD1' | -1.608084983 | 8.78E-09 | 7.54E-07 |
| 25806 | 'VAX2' | -1.085657985 | 2.49E-04 | 0.004494787 |
| 25817 | 'TAFA5' | -1.258106538 | 7.30E-05 | 0.001690663 |
| 25822 | 'DNAJB5' | -0.67771386 | 1.05E-05 | 3.41E-04 |
| 25840 | 'METTL7A' | 0.537240474 | 9.33E-04 | 0.012605026 |
| 25843 | 'MOB4' | -0.359319229 | 7.06E-04 | 0.010142318 |
| 2588 | 'GALNS' | 0.672354281 | 3.62E-07 | 1.96E-05 |
| 25891 | 'PAMR1' | 0.921584581 | 9.33E-04 | 0.012605026 |
| 25896 | 'INTS7' | -0.36388867 | 0.003849722 | 0.037521548 |
| 2590 | 'GALNT2' | 0.414497842 | 0.001292871 | 0.016218618 |
| 25901 | 'CCDC28A' | 0.578203105 | 0.002199061 | 0.024496423 |
| 25906 | 'ANAPC15' | -0.781638316 | 2.31E-05 | 6.52E-04 |
| 25907 | 'TMEM158' | 0.455270746 | 5.37E-04 | 0.008183959 |
| 25912 | 'C1orf43' | -0.366688514 | 3.96E-04 | 0.006426604 |
| 25915 | 'NDUFAF3' | -0.505455815 | 9.79E-05 | 0.002152785 |
| 2592 | 'GALT' | -0.689929312 | 3.68E-04 | 0.006047872 |
| 25920 | 'NELFB' | 0.423500172 | 9.93E-06 | 3.25E-04 |
| 259230 | 'SGMS1' | -0.607174136 | 0.003384041 | 0.034016514 |
| 2593 | 'GAMT' | -0.828339009 | 4.64E-09 | 4.19E-07 |
| 25934 | 'NIPSNAP3A' | -0.501656918 | 0.001948309 | 0.022346389 |
| 25937 | 'WWTR1' | -0.682230936 | 1.48E-05 | 4.53E-04 |
| 25938 | 'HEATR5A' | -0.409747997 | 2.91E-04 | 0.005074064 |
| 25962 | 'VIRMA' | -0.446916788 | 5.12E-05 | 0.001252083 |
| 25966 | 'C2CD2' | 1.261069905 | 1.64E-10 | 1.97E-08 |
| 2597 | 'GAPDH' | -0.461801923 | 7.58E-06 | 2.56E-04 |
| 25975 | 'EGFL6' | -2.322832649 | 0.00443799 | 0.041956035 |
| 25979 | 'DHRS7B' | -0.454372684 | 0.002993117 | 0.031098413 |
| 25989 | 'ULK3' | 0.338022368 | 0.003016823 | 0.031249208 |
| 25992 | 'SNED1' | 1.650952355 | 5.77E-06 | 2.02E-04 |
| 26002 | 'MOXD1' | 1.246297586 | 0.003157975 | 0.032278564 |
| 26012 | 'NSMF' | -0.454274457 | 0.00144136 | 0.017593065 |
| 26015 | 'RPAP1' | -0.394351771 | 0.003790415 | 0.037007168 |
| 26017 | 'FAM32A' | 0.492194118 | 1.92E-09 | 1.92E-07 |
| 26036 | 'ZNF451' | 0.643051671 | 1.32E-05 | 4.10E-04 |
| 26039 | 'SS18L1' | 0.514586478 | 6.96E-04 | 0.010009605 |
| 26060 | 'APPL1' | -2.173329073 | 3.29E-48 | 1.86E-44 |
| 26064 | 'RAI14' | -0.420646793 | 4.94E-05 | 0.001219572 |
| 26065 | 'LSM14A' | -0.371848888 | 3.04E-04 | 0.005226921 |
| 26099 | 'SZRD1' | -0.265954185 | 0.003986746 | 0.038481949 |
| 26100 | 'WIPI2' | 0.39576808 | 0.001411594 | 0.017343542 |
| 26130 | 'GAPVD1' | 0.32673991 | 8.89E-04 | 0.012226008 |
| 26207 | 'PITPNC1' | 0.555161907 | 0.001897405 | 0.021880662 |
| 2621 | 'GAS6' | 0.638809231 | 1.22E-04 | 0.002564545 |
| 2622 | 'GAS8' | 0.580657754 | 2.90E-04 | 0.005069194 |
| 26225 | 'ARL5A' | 0.459173649 | 3.04E-05 | 8.15E-04 |
| 26258 | 'BLOC1S6' | -0.413346617 | 4.32E-06 | 1.57E-04 |
| 26277 | 'TINF2' | -0.607030733 | 7.59E-07 | 3.61E-05 |
| 2629 | 'GBA' | -0.271642877 | 1.49E-04 | 0.002999617 |
| 26292 | 'MYCBP' | 0.706227505 | 3.97E-04 | 0.006426604 |
| 26301 | 'GBGT1' | -1.055301354 | 0.003254651 | 0.032907047 |
| 2633 | 'GBP1' | -0.977213448 | 2.64E-07 | 1.50E-05 |
| 2644 | 'GCHFR' | -1.10902235 | 0.004087697 | 0.039277934 |
| 26472 | 'PPP1R14B' | 0.721043908 | 5.13E-12 | 8.47E-10 |
| 2648 | 'KAT2A' | 0.364306241 | 0.003411148 | 0.034228274 |
| 26503 | 'SLC17A5' | -1.770921116 | 1.01E-13 | 2.13E-11 |
| 26509 | 'MYOF' | -1.754572151 | 4.52E-20 | 2.33E-17 |
| 2653 | 'GCSH' | 0.374589962 | 0.00217441 | 0.024281186 |
| 26577 | 'PCOLCE2' | 0.92078066 | 7.29E-05 | 0.001689375 |
| 266727 | 'MDGA1' | -1.078915163 | 7.87E-05 | 0.001788291 |
| 2668 | 'GDNF' | 0.553014537 | 0.001290604 | 0.016209665 |
| 2673 | 'GFPT1' | 0.408562868 | 1.96E-04 | 0.003752662 |
| 26873 | 'OPLAH' | -0.532507894 | 0.002375129 | 0.026131028 |
| 26953 | 'RANBP6' | 0.461891753 | 0.003149501 | 0.032250132 |
| 26999 | 'CYFIP2' | -1.138277221 | 6.21E-05 | 0.001474303 |
| 27030 | 'MLH3' | 0.674201586 | 2.63E-04 | 0.004693466 |
| 27031 | 'NPHP3' | 0.399758123 | 0.005267217 | 0.048083864 |
| 27032 | 'ATP2C1' | -0.393072646 | 0.001147595 | 0.014733251 |
| 2706 | 'GJB2' | 2.128546826 | 0.003209628 | 0.032629941 |
| 27148 | 'STK36' | 0.526898881 | 1.65E-05 | 4.93E-04 |
| 27163 | 'NAAA' | 0.616774271 | 3.88E-04 | 0.006323494 |
| 27235 | 'COQ2' | 0.51393177 | 8.07E-04 | 0.011327475 |
| 27244 | 'SESN1' | -0.528608129 | 0.002988466 | 0.031088093 |
| 27246 | 'RNF115' | -0.39465206 | 2.04E-04 | 0.00385497 |
| 27286 | 'SRPX2' | 0.468904892 | 8.39E-04 | 0.011642243 |
| 27300 | 'ZNF544' | 0.350221182 | 0.005154962 | 0.047300512 |
| 27315 | 'PGAP2' | -0.642786363 | 0.005042238 | 0.046596403 |
| 27327 | 'TNRC6A' | 0.287083067 | 3.34E-04 | 0.005586 |
| 2733 | 'GLE1' | 0.429725964 | 0.002080107 | 0.023493458 |
| 27350 | 'APOBEC3C' | -0.771640359 | 2.10E-06 | 8.54E-05 |
| 2736 | 'GLI2' | -1.47260717 | 3.92E-06 | 1.45E-04 |
| 2744 | 'GLS' | -0.997053476 | 4.43E-05 | 0.001113971 |
| 2745 | 'GLRX' | 0.717394981 | 6.32E-05 | 0.001499311 |
| 2746 | 'GLUD1' | -0.310446215 | 0.004574869 | 0.042913365 |
| 2768 | 'GNA12' | 0.420915814 | 0.001582463 | 0.018876283 |
| 2770 | 'GNAI1' | -0.511075834 | 6.64E-05 | 0.001560546 |
| 2783 | 'GNB2' | -0.302794997 | 0.005084022 | 0.046915425 |
| 2801 | 'GOLGA2' | 0.297446597 | 5.59E-05 | 0.001350369 |
| 2802 | 'GOLGA3' | 0.436478654 | 1.53E-04 | 0.003057307 |
| 2812 | 'GP1BB' | -1.893305094 | 3.17E-04 | 0.005383589 |
| 2817 | 'GPC1' | -2.110278467 | 6.12E-39 | 1.73E-35 |
| 28232 | 'SLCO3A1' | -0.712724192 | 0.003234042 | 0.032811654 |
| 283149 | 'BCL9L' | -0.305203095 | 0.003010362 | 0.031210951 |
| 283310 | 'OTOGL' | -1.620924708 | 2.28E-06 | 9.19E-05 |
| 283373 | 'ANKRD52' | -0.475182086 | 1.56E-05 | 4.73E-04 |
| 283459 | 'GATC' | -0.555784758 | 8.51E-07 | 3.99E-05 |
| 283464 | 'GXYLT1' | 0.797978026 | 4.36E-06 | 1.58E-04 |
| 283742 | 'FAM98B' | -0.400257287 | 0.001283525 | 0.016137103 |
| 283991 | 'UBALD2' | 0.665632206 | 3.76E-05 | 9.79E-04 |
| 284001 | 'CCDC57' | 0.863897225 | 0.001281581 | 0.016136305 |
| 284217 | 'LAMA1' | 0.507317985 | 0.001004985 | 0.013293955 |
| 284370 | 'ZNF615' | 0.551925615 | 8.35E-04 | 0.011602167 |
| 284371 | 'ZNF841' | 0.767964444 | 3.48E-07 | 1.90E-05 |
| 2844 | 'GPR21' | -4.048732735 | 7.23E-04 | 0.010327384 |
| 284459 | 'ZNF875' | 0.65886282 | 2.09E-05 | 5.98E-04 |
| 284565 | 'NBPF15' | 0.295416534 | 0.003118611 | 0.031991643 |
| 285 | 'ANGPT2' | 1.692211005 | 4.15E-10 | 4.66E-08 |
| 285203 | 'EOGT' | -0.463437847 | 8.44E-04 | 0.011698696 |
| 285331 | 'CCDC66' | 0.556848493 | 5.30E-04 | 0.008097008 |
| 285381 | 'DPH3' | 0.458042448 | 1.32E-05 | 4.10E-04 |
| 285598 | 'ARL10' | 0.484335258 | 5.51E-04 | 0.008351833 |
| 285636 | 'C5orf51' | -0.967487121 | 1.37E-16 | 4.33E-14 |
| 285672 | 'SREK1IP1' | -0.756504719 | 5.83E-06 | 2.03E-04 |
| 2863 | 'GPR39' | 1.678165605 | 0.0028525 | 0.030060005 |
| 286410 | 'ATP11C' | -0.537161929 | 2.02E-05 | 5.82E-04 |
| 286827 | 'TRIM59' | -0.767235884 | 0.002475801 | 0.02694217 |
| 2872 | 'MKNK2' | 1.001411061 | 4.53E-10 | 5.00E-08 |
| 2878 | 'GPX3' | 1.700029344 | 7.17E-08 | 4.84E-06 |
| 2882 | 'GPX7' | -1.150038828 | 6.43E-04 | 0.009436103 |
| 2887 | 'GRB10' | 0.604547292 | 3.14E-04 | 0.005354657 |
| 28951 | 'TRIB2' | 0.595295748 | 1.52E-04 | 0.003041728 |
| 28962 | 'OSTM1' | -0.730721159 | 4.15E-04 | 0.006645783 |
| 28988 | 'DBNL' | -0.435220416 | 3.65E-06 | 1.37E-04 |
| 28992 | 'MACROD1' | 0.697592665 | 0.001354123 | 0.016817326 |
| 290 | 'ANPEP' | 0.618086108 | 2.34E-04 | 0.004273708 |
| 29058 | 'TMEM230' | -0.299809777 | 3.90E-05 | 0.001011937 |
| 29066 | 'ZC3H7A' | -0.425191476 | 2.33E-04 | 0.00427349 |
| 29068 | 'ZBTB44' | 0.387600773 | 0.0014469 | 0.017630404 |
| 29071 | 'C1GALT1C1' | 0.770467848 | 1.27E-05 | 3.99E-04 |
| 29083 | 'GTPBP8' | -0.558939089 | 0.003091289 | 0.031788102 |
| 2909 | 'ARHGAP35' | 0.248282917 | 0.004428013 | 0.041908337 |
| 29095 | 'ORMDL2' | -0.456261279 | 6.12E-04 | 0.009032616 |
| 29097 | 'CNIH4' | 0.316392938 | 6.47E-04 | 0.009469716 |
| 29105 | 'CFAP20' | -0.81231448 | 1.73E-11 | 2.52E-09 |
| 2935 | 'GSPT1' | 0.198353293 | 0.003723397 | 0.036562859 |
| 2946 | 'GSTM2' | -0.607175484 | 0.00378409 | 0.036966641 |
| 2971 | 'GTF3A' | 0.407510103 | 0.001774012 | 0.020729911 |
| 2979 | 'GUCA1B' | 1.722783244 | 0.004833635 | 0.044971063 |
| 29843 | 'SENP1' | -0.407634252 | 0.002279216 | 0.025288582 |
| 2990 | 'GUSB' | 0.508469295 | 4.20E-05 | 0.001066541 |
| 29904 | 'EEF2K' | 0.574949536 | 7.61E-04 | 0.010786071 |
| 29916 | 'SNX11' | -0.400601184 | 0.001107869 | 0.014372616 |
| 29924 | 'EPN1' | 0.497240707 | 7.28E-07 | 3.50E-05 |
| 29937 | 'NENF' | 0.473704466 | 2.24E-04 | 0.004154912 |
| 29942 | 'PURG' | -1.980936139 | 0.003436884 | 0.034405275 |
| 29957 | 'SLC25A24' | -0.919631346 | 2.67E-20 | 1.46E-17 |
| 29959 | 'NRBP1' | 0.329346875 | 0.003271039 | 0.033017291 |
| 29965 | 'CDIP1' | -0.398309116 | 1.90E-05 | 5.55E-04 |
| 29966 | 'STRN3' | -0.449128867 | 4.08E-04 | 0.006567999 |
| 29968 | 'PSAT1' | 1.007381728 | 2.56E-04 | 0.004587402 |
| 29969 | 'MDFIC' | -0.497727315 | 0.004503117 | 0.042383155 |
| 29994 | 'BAZ2B' | -0.491098514 | 0.001247099 | 0.015807744 |
| 29995 | 'LMCD1' | -1.360524062 | 4.08E-05 | 0.001044449 |
| 30001 | 'ERO1A' | 0.979292546 | 8.04E-19 | 3.42E-16 |
| 3005 | 'H1-0' | 0.429310667 | 3.32E-05 | 8.80E-04 |
| 30061 | 'SLC40A1' | -1.238596161 | 0.00216305 | 0.024213227 |
| 301 | 'ANXA1' | 0.361029607 | 1.04E-04 | 0.002244768 |
| 3017 | 'H2BC5' | 1.049904334 | 0.00170187 | 0.020033509 |
| 302 | 'ANXA2' | -0.343501583 | 0.002254082 | 0.025026053 |
| 3032 | 'HADHB' | 0.281521431 | 3.34E-04 | 0.005586 |
| 3037 | 'HAS2' | -1.105712872 | 2.03E-08 | 1.60E-06 |
| 3038 | 'HAS3' | -2.708345903 | 9.39E-06 | 3.11E-04 |
| 3059 | 'HCLS1' | 0.947972113 | 0.003519857 | 0.035091222 |
| 3074 | 'HEXB' | 0.322039768 | 0.004668414 | 0.043648904 |
| 3075 | 'CFH' | 0.326783846 | 8.20E-04 | 0.011421494 |
| 308 | 'ANXA5' | -0.226099508 | 8.10E-04 | 0.011327475 |
| 30832 | 'ZNF354C' | 0.552217308 | 2.24E-04 | 0.004154912 |
| 30844 | 'EHD4' | -0.589347356 | 8.80E-04 | 0.01212104 |
| 3094 | 'HINT1' | -0.195851618 | 0.003975578 | 0.038461513 |
| 310 | 'ANXA7' | 0.471586627 | 7.81E-09 | 6.78E-07 |
| 3106 | 'HLA-B' | 0.511156656 | 0.00258131 | 0.027682715 |
| 3107 | 'HLA-C' | 0.393823618 | 0.005529085 | 0.049911521 |
| 3115 | 'HLA-DPB1' | -0.610562349 | 0.002323767 | 0.025699021 |
| 3133 | 'HLA-E' | -0.496954609 | 5.03E-06 | 1.79E-04 |
| 3159 | 'HMGA1' | 1.127543477 | 1.71E-05 | 5.05E-04 |
| 316 | 'AOX1' | 1.47808687 | 5.93E-05 | 0.001420009 |
| 317 | 'APAF1' | -0.606056885 | 4.20E-05 | 0.001066541 |
| 3176 | 'HNMT' | -0.420590883 | 2.95E-04 | 0.005123408 |
| 3182 | 'HNRNPAB' | 0.370503202 | 0.00353431 | 0.035194021 |
| 3187 | 'HNRNPH1' | 0.312596281 | 2.83E-05 | 7.73E-04 |
| 320 | 'APBA1' | -0.754085506 | 1.25E-07 | 7.83E-06 |
| 3204 | 'HOXA7' | -0.925720409 | 1.24E-04 | 0.002597264 |
| 3212 | 'HOXB2' | -0.467824545 | 0.002856997 | 0.030088749 |
| 323 | 'APBB2' | -0.373809383 | 8.19E-04 | 0.011404299 |
| 3235 | 'HOXD9' | -2.093257604 | 0.001009056 | 0.013327066 |
| 3265 | 'HRAS' | -0.644115649 | 9.06E-07 | 4.19E-05 |
| 328 | 'APEX1' | -0.374034234 | 1.47E-04 | 0.002972232 |
| 3303 | 'HSPA1A' | -0.41857068 | 0.003598008 | 0.035712833 |
| 3306 | 'HSPA2' | -1.239973964 | 0.002538926 | 0.027405377 |
| 3308 | 'HSPA4' | 0.30815415 | 0.001237285 | 0.015718511 |
| 3309 | 'HSPA5' | 0.912800573 | 9.59E-06 | 3.16E-04 |
| 3315 | 'HSPB1' | -0.790692951 | 2.82E-05 | 7.72E-04 |
| 3320 | 'HSP90AA1' | -0.239779153 | 0.001497533 | 0.018104602 |
| 334 | 'APLP2' | -1.366179922 | 1.25E-27 | 1.63E-24 |
| 3340 | 'NDST1' | -0.461960156 | 1.01E-07 | 6.51E-06 |
| 337876 | 'CHSY3' | -1.039394429 | 1.44E-08 | 1.18E-06 |
| 338692 | 'ANKRD13D' | 0.429037163 | 0.001172868 | 0.0150235 |
| 339123 | 'JMJD8' | 0.418031945 | 1.83E-04 | 0.003566045 |
| 340061 | 'STING1' | -0.713050855 | 8.62E-06 | 2.88E-04 |
| 340371 | 'NRBP2' | -0.836962185 | 4.57E-06 | 1.64E-04 |
| 340485 | 'ACER2' | -1.255889968 | 0.003993026 | 0.038520685 |
| 340719 | 'NANOS1' | 1.31125822 | 0.001291204 | 0.016209665 |
| 3418 | 'IDH2' | -0.653478848 | 9.79E-04 | 0.013066128 |
| 3428 | 'IFI16' | 0.331404513 | 0.001897185 | 0.021880662 |
| 342909 | 'ZNF284' | 0.672930331 | 0.001355828 | 0.016817326 |
| 3430 | 'IFI35' | -0.674269011 | 0.001441909 | 0.017593065 |
| 344148 | 'NCKAP5' | 0.964527037 | 9.35E-04 | 0.012613942 |
| 345557 | 'PLCXD3' | 6.290124894 | 1.17E-05 | 3.70E-04 |
| 347 | 'APOD' | 1.610326892 | 2.15E-04 | 0.004015408 |
| 347734 | 'SLC35B2' | -0.37051367 | 0.003708145 | 0.036539739 |
| 347902 | 'AMIGO2' | -1.195306437 | 0.001134309 | 0.014608752 |
| 3484 | 'IGFBP1' | 4.005127239 | 7.41E-07 | 3.56E-05 |
| 3487 | 'IGFBP4' | 0.548792393 | 0.004743174 | 0.044274831 |
| 353088 | 'ZNF429' | 0.77731799 | 0.00241135 | 0.026409874 |
| 355 | 'FAS' | -0.887844101 | 4.26E-08 | 3.12E-06 |
| 3554 | 'IL1R1' | 0.608596 | 4.57E-05 | 0.001142978 |
| 3589 | 'IL11' | -2.066196251 | 2.25E-04 | 0.004163761 |
| 3590 | 'IL11RA' | -0.971997761 | 6.88E-04 | 0.009930613 |
| 3597 | 'IL13RA1' | -0.42814946 | 3.93E-04 | 0.006392444 |
| 3624 | 'INHBA' | -1.020155862 | 3.62E-04 | 0.005956788 |
| 3633 | 'INPP5B' | 0.626275983 | 9.53E-05 | 0.002106548 |
| 3652 | 'IPP' | -0.694324341 | 0.005342758 | 0.048466252 |
| 366 | 'AQP9' | 3.757322839 | 5.69E-05 | 0.001370504 |
| 3672 | 'ITGA1' | -0.742054587 | 4.64E-07 | 2.42E-05 |
| 3685 | 'ITGAV' | -0.428834782 | 2.09E-04 | 0.003934509 |
| 3699 | 'ITIH3' | -2.292758945 | 0.001126175 | 0.014557207 |
| 3707 | 'ITPKB' | -0.536303681 | 0.003562766 | 0.035435863 |
| 3720 | 'JARID2' | 0.707777257 | 4.12E-05 | 0.001051317 |
| 3727 | 'JUND' | 0.455852923 | 0.002968166 | 0.030959202 |
| 3735 | 'KARS1' | 0.320149391 | 7.54E-05 | 0.001735237 |
| 374395 | 'TMEM179B' | -0.529709262 | 3.98E-07 | 2.13E-05 |
| 374887 | 'YJEFN3' | 1.372802945 | 0.001594241 | 0.018990124 |
| 3749 | 'KCNC4' | -0.948260388 | 3.57E-06 | 1.35E-04 |
| 3750 | 'KCND1' | -1.29973756 | 0.00165833 | 0.019616074 |
| 375061 | 'FAM89A' | -0.98316237 | 7.27E-05 | 0.001687485 |
| 375346 | 'STIMATE' | -0.554689581 | 1.06E-06 | 4.79E-05 |
| 375484 | 'SIMC1' | -0.533340212 | 0.00176246 | 0.020646652 |
| 3756 | 'KCNH1' | -1.154942036 | 0.004053448 | 0.039013632 |
| 3757 | 'KCNH2' | -5.521262761 | 0.001138344 | 0.014636585 |
| 3759 | 'KCNJ2' | 1.176457483 | 9.94E-04 | 0.013179931 |
| 376267 | 'RAB15' | -0.648985118 | 3.04E-05 | 8.15E-04 |
| 3764 | 'KCNJ8' | -0.829701679 | 0.004635335 | 0.043363472 |
| 377007 | 'KLHL30' | -2.448265572 | 6.50E-04 | 0.009506244 |
| 378 | 'ARF4' | -0.804419009 | 9.02E-25 | 7.67E-22 |
| 3788 | 'KCNS2' | 2.016499632 | 2.19E-05 | 6.24E-04 |
| 3790 | 'KCNS3' | 0.577747572 | 0.001060929 | 0.013882737 |
| 3799 | 'KIF5B' | -0.338002861 | 4.21E-04 | 0.006704614 |
| 38 | 'ACAT1' | 0.572117404 | 5.69E-08 | 3.97E-06 |
| 3836 | 'KPNA1' | 0.264878339 | 3.01E-04 | 0.005193706 |
| 3842 | 'TNPO1' | -0.367136586 | 0.002322073 | 0.025699021 |
| 3872 | 'KRT17' | -1.329405052 | 1.04E-04 | 0.002246141 |
| 387357 | 'THEMIS' | -2.232697392 | 0.00148521 | 0.017989965 |
| 387893 | 'KMT5A' | -0.400089465 | 2.71E-04 | 0.004814243 |
| 388121 | 'TNFAIP8L3' | 0.780594599 | 4.51E-09 | 4.10E-07 |
| 388125 | 'C2CD4B' | 4.395481365 | 0.004779068 | 0.044536509 |
| 388341 | 'LRRC75A' | -0.524044906 | 6.39E-07 | 3.15E-05 |
| 388561 | 'ZNF761' | 0.605904386 | 1.65E-05 | 4.93E-04 |
| 388610 | 'TRNP1' | -0.955211118 | 2.58E-04 | 0.004625762 |
| 388630 | 'TRABD2B' | 1.033664064 | 6.03E-04 | 0.008943946 |
| 388677 | 'NOTCH2NLA' | -0.953695667 | 0.002742891 | 0.029067124 |
| 388969 | 'C2orf68' | -0.514510037 | 1.10E-04 | 0.002353841 |
| 389072 | 'PLEKHM3' | -0.574871384 | 9.04E-05 | 0.002012148 |
| 389136 | 'VGLL3' | -0.388456468 | 1.32E-04 | 0.002735976 |
| 389336 | 'C5orf46' | -2.075227904 | 0.001282513 | 0.016136305 |
| 389541 | 'LAMTOR4' | -0.638564177 | 6.77E-06 | 2.32E-04 |
| 3903 | 'LAIR1' | -1.350751236 | 0.00110821 | 0.014372616 |
| 390595 | 'UBAP1L' | 0.963862507 | 9.53E-04 | 0.012792694 |
| 3911 | 'LAMA5' | -1.889084417 | 1.80E-06 | 7.56E-05 |
| 3913 | 'LAMB2' | -0.506468948 | 2.75E-04 | 0.004867757 |
| 3925 | 'STMN1' | 0.493927764 | 0.001052899 | 0.013788272 |
| 3927 | 'LASP1' | -0.716327664 | 7.70E-07 | 3.65E-05 |
| 3955 | 'LFNG' | -1.060215901 | 4.16E-05 | 0.001059206 |
| 3984 | 'LIMK1' | 0.733938592 | 4.46E-04 | 0.007003422 |
| 3988 | 'LIPA' | 0.557423227 | 1.36E-06 | 5.94E-05 |
| 3992 | 'FADS1' | -0.576359107 | 2.74E-05 | 7.51E-04 |
| 399512 | 'SLC25A35' | -0.831882169 | 0.001806715 | 0.021049034 |
| 399909 | 'PCNX3' | 0.697237103 | 7.17E-09 | 6.35E-07 |
| 400451 | 'FAM174B' | -1.48398132 | 0.003011293 | 0.031210951 |
| 4008 | 'LMO7' | -0.670747525 | 8.07E-04 | 0.011327475 |
| 4012 | 'LNPEP' | -0.365980114 | 4.94E-04 | 0.007616475 |
| 4023 | 'LPL' | 3.027697633 | 5.54E-13 | 1.05E-10 |
| 402317 | 'OR2A42' | -5.929320206 | 7.69E-05 | 0.001759264 |
| 4038 | 'LRP4' | -1.263085306 | 2.31E-04 | 0.004242302 |
| 4041 | 'LRP5' | -0.473250574 | 6.75E-04 | 0.009796338 |
| 404217 | 'CTXN1' | 1.464513986 | 4.03E-08 | 2.98E-06 |
| 4043 | 'LRPAP1' | 0.336926905 | 3.91E-06 | 1.45E-04 |
| 406 | 'ARNTL' | 0.640862701 | 0.001029492 | 0.013523426 |
| 4067 | 'LYN' | -0.504537128 | 0.005312544 | 0.048290175 |
| 4070 | 'TACSTD2' | 1.283279001 | 3.94E-05 | 0.001017089 |
| 4071 | 'TM4SF1' | 1.131591631 | 2.97E-05 | 8.01E-04 |
| 4076 | 'CAPRIN1' | -0.697497458 | 4.82E-16 | 1.41E-13 |
| 41 | 'ASIC1' | -1.477829839 | 5.46E-05 | 0.001323048 |
| 410 | 'ARSA' | -0.589671897 | 0.00438617 | 0.041535446 |
| 411 | 'ARSB' | -0.378645528 | 1.93E-07 | 1.13E-05 |
| 4124 | 'MAN2A1' | -1.379573157 | 2.90E-04 | 0.005069194 |
| 4133 | 'MAP2' | 1.114057098 | 0.001581056 | 0.018872749 |
| 414189 | 'AGAP6' | 0.852200029 | 3.81E-08 | 2.84E-06 |
| 4147 | 'MATN2' | 1.489673523 | 8.16E-06 | 2.75E-04 |
| 4150 | 'MAZ' | 0.405050908 | 0.004096262 | 0.039325069 |
| 4152 | 'MBD1' | 0.311952176 | 3.72E-04 | 0.006097146 |
| 4162 | 'MCAM' | -1.631399906 | 4.70E-25 | 4.21E-22 |
| 4163 | 'MCC' | -0.948900679 | 9.43E-05 | 0.002086843 |
| 4179 | 'CD46' | 0.482710585 | 3.40E-05 | 8.97E-04 |
| 4204 | 'MECP2' | -0.370713173 | 2.83E-06 | 1.12E-04 |
| 4209 | 'MEF2D' | -0.322155718 | 0.004097229 | 0.039325069 |
| 4216 | 'MAP3K4' | -0.659244162 | 2.32E-04 | 0.004267581 |
| 4233 | 'MET' | 0.435028877 | 5.15E-05 | 0.001256162 |
| 4240 | 'MFGE8' | -0.650367666 | 0.001316323 | 0.016440008 |
| 4241 | 'MELTF' | -1.765459963 | 0.001108513 | 0.014372616 |
| 4256 | 'MGP' | 2.048519958 | 9.11E-08 | 6.02E-06 |
| 4258 | 'MGST2' | -0.709350728 | 4.95E-04 | 0.007627942 |
| 4281 | 'MID1' | -0.815310472 | 1.14E-13 | 2.37E-11 |
| 4285 | 'MIPEP' | 0.323384014 | 0.003444682 | 0.034463043 |
| 4301 | 'AFDN' | 0.515764679 | 3.12E-04 | 0.005326915 |
| 4320 | 'MMP11' | -0.957340091 | 6.79E-05 | 0.001593583 |
| 4324 | 'MMP15' | 0.499092162 | 9.30E-07 | 4.30E-05 |
| 4335 | 'MNT' | 0.412401395 | 4.06E-04 | 0.006554031 |
| 4345 | 'CD200' | -1.673596958 | 0.001530511 | 0.018372621 |
| 440026 | 'TMEM41B' | 0.603072626 | 0.002695284 | 0.028651931 |
| 440345 | 'NPIPB4' | 0.586572931 | 5.19E-06 | 1.84E-04 |
| 440515 | 'ZNF506' | 0.537872012 | 0.001947207 | 0.022346389 |
| 440804 | 'RIMBP3B' | -4.874378782 | 9.92E-04 | 0.013174879 |
| 441024 | 'MTHFD2L' | 0.982636579 | 8.39E-04 | 0.011642243 |
| 441631 | 'TSPAN11' | 1.113870425 | 7.80E-04 | 0.011024871 |
| 4493 | 'MT1E' | 1.262542837 | 3.36E-04 | 0.005618515 |
| 4499 | 'MT1M' | 1.732129381 | 0.0036122 | 0.035780985 |
| 4501 | 'MT1X' | 1.4174854 | 4.32E-05 | 0.001089843 |
| 4502 | 'MT2A' | 1.01968073 | 3.62E-04 | 0.005956788 |
| 4520 | 'MTF1' | 0.462251485 | 0.002340037 | 0.025811777 |
| 4534 | 'MTM1' | 0.770581683 | 3.73E-05 | 9.74E-04 |
| 4599 | 'MX1' | -1.467058368 | 0.003600011 | 0.035712833 |
| 4600 | 'MX2' | -1.472142957 | 2.19E-04 | 0.004082729 |
| 4609 | 'MYC' | 0.847309715 | 7.11E-06 | 2.42E-04 |
| 4627 | 'MYH9' | -0.694739694 | 2.83E-07 | 1.60E-05 |
| 4637 | 'MYL6' | -0.55244554 | 2.98E-06 | 1.17E-04 |
| 4638 | 'MYLK' | -1.57565324 | 5.61E-60 | 4.77E-56 |
| 4641 | 'MYO1C' | -0.43808102 | 5.51E-05 | 0.00133329 |
| 4659 | 'PPP1R12A' | -1.214319984 | 5.22E-17 | 1.74E-14 |
| 4660 | 'PPP1R12B' | -0.601627379 | 9.53E-04 | 0.012792694 |
| 4675 | 'NAP1L3' | -1.284165318 | 1.84E-12 | 3.16E-10 |
| 4696 | 'NDUFA3' | -0.430107805 | 3.96E-04 | 0.006424569 |
| 4707 | 'NDUFB1' | -0.437606589 | 7.45E-04 | 0.010599442 |
| 4718 | 'NDUFC2' | -0.916356825 | 5.25E-26 | 4.95E-23 |
| 4720 | 'NDUFS2' | 0.350661911 | 1.45E-04 | 0.002953105 |
| 4726 | 'NDUFS6' | -0.325480996 | 0.004174641 | 0.039932783 |
| 4735 | 'SEPTIN2' | -0.205488966 | 0.004590137 | 0.043011659 |
| 4739 | 'NEDD9' | -0.487528399 | 9.38E-08 | 6.18E-06 |
| 4744 | 'NEFH' | 1.330437911 | 2.28E-04 | 0.004203776 |
| 4776 | 'NFATC4' | -0.455291775 | 1.93E-04 | 0.003711835 |
| 481 | 'ATP1B1' | -0.642182273 | 2.44E-05 | 6.83E-04 |
| 4817 | 'NIT1' | -0.474320333 | 0.002345114 | 0.025851001 |
| 483 | 'ATP1B3' | 0.344493483 | 0.002045977 | 0.023200478 |
| 4833 | 'NME4' | -0.234061232 | 0.005335363 | 0.048445776 |
| 4837 | 'NNMT' | -0.480675329 | 1.09E-05 | 3.47E-04 |
| 4853 | 'NOTCH2' | -0.313027622 | 6.85E-04 | 0.009895151 |
| 4854 | 'NOTCH3' | -0.852946602 | 0.001006352 | 0.013301688 |
| 4862 | 'NPAS2' | -0.536325333 | 0.001353487 | 0.016817326 |
| 4867 | 'NPHP1' | 0.885504042 | 1.39E-04 | 0.00284474 |
| 4904 | 'YBX1' | 0.319472311 | 3.23E-06 | 1.23E-04 |
| 4907 | 'NT5E' | 0.646968907 | 8.16E-08 | 5.42E-06 |
| 4915 | 'NTRK2' | -1.340904056 | 0.004915594 | 0.045633683 |
| 4925 | 'NUCB2' | 0.401795348 | 5.76E-04 | 0.008651075 |
| 4929 | 'NR4A2' | 1.636955751 | 5.04E-05 | 0.001237169 |
| 4931 | 'NVL' | 0.362130785 | 0.00314755 | 0.032249576 |
| 493753 | 'COA5' | 0.525232103 | 5.83E-04 | 0.008733946 |
| 4938 | 'OAS1' | -2.307574887 | 4.09E-04 | 0.006577381 |
| 4952 | 'OCRL' | -0.485315859 | 1.49E-06 | 6.41E-05 |
| 4968 | 'OGG1' | -0.662148525 | 1.89E-04 | 0.003672576 |
| 4983 | 'OPHN1' | -0.499015325 | 0.002017562 | 0.022954832 |
| 50 | 'ACO2' | 0.428028836 | 2.98E-08 | 2.28E-06 |
| 5007 | 'OSBP' | 0.366771553 | 2.71E-05 | 7.44E-04 |
| 501 | 'ALDH7A1' | -0.493296194 | 0.002781074 | 0.029398447 |
| 5016 | 'OVGP1' | 1.917700387 | 2.01E-04 | 0.003823726 |
| 5025 | 'P2RX4' | 0.395487195 | 0.0054189 | 0.049047104 |
| 5031 | 'P2RY6' | 1.376132887 | 4.32E-04 | 0.006831404 |
| 5034 | 'P4HB' | 0.423835557 | 1.67E-05 | 4.99E-04 |
| 5037 | 'PEBP1' | 0.270917434 | 0.002437654 | 0.026626196 |
| 5045 | 'FURIN' | 0.416371133 | 2.07E-07 | 1.20E-05 |
| 50484 | 'RRM2B' | 0.51621905 | 0.003712988 | 0.03656034 |
| 5049 | 'PAFAH1B2' | 0.380539179 | 2.91E-04 | 0.005074064 |
| 50509 | 'COL5A3' | -1.022697122 | 2.48E-05 | 6.91E-04 |
| 50512 | 'PODXL2' | 1.144084946 | 1.66E-04 | 0.003276591 |
| 50650 | 'ARHGEF3' | 0.623607553 | 4.34E-07 | 2.29E-05 |
| 5069 | 'PAPPA' | -1.071527825 | 0.005224964 | 0.047817599 |
| 5074 | 'PAWR' | -0.363655956 | 9.26E-04 | 0.012567284 |
| 50807 | 'ASAP1' | -0.506651994 | 0.001355614 | 0.016817326 |
| 50862 | 'RNF141' | -0.383758298 | 2.12E-04 | 0.003971023 |
| 50999 | 'TMED5' | 0.336844833 | 4.92E-06 | 1.76E-04 |
| 5101 | 'PCDH9' | -0.652875155 | 0.002111673 | 0.023786755 |
| 51013 | 'EXOSC1' | -0.379476574 | 0.001544352 | 0.018512615 |
| 51060 | 'TXNDC12' | 0.485993011 | 4.40E-08 | 3.19E-06 |
| 51062 | 'ATL1' | -0.559705087 | 0.002911233 | 0.030581997 |
| 51091 | 'SEPSECS' | 0.674576639 | 1.32E-04 | 0.002735976 |
| 51092 | 'SIDT2' | -0.261398342 | 0.003244 | 0.032881041 |
| 5110 | 'PCMT1' | 0.338117214 | 3.68E-05 | 9.63E-04 |
| 51103 | 'NDUFAF1' | 0.488881975 | 8.56E-04 | 0.011854326 |
| 51106 | 'TFB1M' | 0.628175167 | 2.77E-04 | 0.004886946 |
| 51108 | 'METTL9' | 0.609252415 | 4.29E-14 | 1.00E-11 |
| 51114 | 'ZDHHC9' | 0.440531741 | 4.40E-06 | 1.60E-04 |
| 51117 | 'COQ4' | 0.570598545 | 2.50E-04 | 0.00450504 |
| 51119 | 'SBDS' | 0.347344254 | 6.25E-06 | 2.16E-04 |
| 51129 | 'ANGPTL4' | 1.090144629 | 6.65E-06 | 2.28E-04 |
| 51150 | 'SDF4' | 0.469545827 | 7.48E-07 | 3.58E-05 |
| 51175 | 'TUBE1' | 0.644783599 | 0.00155298 | 0.018602929 |
| 51182 | 'HSPA14' | 0.426184856 | 0.003235224 | 0.032811654 |
| 51227 | 'PIGP' | -0.539635458 | 0.00259719 | 0.027817915 |
| 51228 | 'GLTP' | 0.291850181 | 0.004970597 | 0.046068813 |
| 51232 | 'CRIM1' | -0.619732785 | 1.06E-08 | 8.93E-07 |
| 51234 | 'EMC4' | -0.325874846 | 0.003946677 | 0.038268636 |
| 51247 | 'PAIP2' | 0.297983167 | 5.51E-04 | 0.008351833 |
| 51248 | 'PDZD11' | -0.396913774 | 0.004552987 | 0.042778919 |
| 51253 | 'MRPL37' | -0.330871605 | 4.15E-04 | 0.006645783 |
| 51257 | 'MARCHF2' | 0.453831401 | 0.001261421 | 0.015918422 |
| 51259 | 'TMEM216' | 0.650482758 | 0.004136885 | 0.039616207 |
| 51263 | 'MRPL30' | -0.654134974 | 1.44E-05 | 4.43E-04 |
| 51272 | 'BET1L' | -0.281500522 | 0.001914842 | 0.022045044 |
| 51277 | 'DNAJC27' | -0.660219567 | 0.002512357 | 0.027200663 |
| 51280 | 'GOLM1' | 0.623033796 | 1.47E-10 | 1.78E-08 |
| 51290 | 'ERGIC2' | 0.429050175 | 3.23E-04 | 0.00545082 |
| 51293 | 'CD320' | 0.636289921 | 9.28E-04 | 0.012574211 |
| 51299 | 'NRN1' | -0.55866642 | 0.004384401 | 0.041535446 |
| 51301 | 'GCNT4' | -1.8952091 | 1.06E-08 | 8.93E-07 |
| 51313 | 'GASK1B' | -0.703358044 | 0.005259105 | 0.048035606 |
| 51332 | 'SPTBN5' | -1.174964205 | 0.002349592 | 0.025883583 |
| 51339 | 'DACT1' | -0.830439873 | 1.01E-07 | 6.51E-06 |
| 51351 | 'ZNF117' | 1.298779274 | 3.21E-05 | 8.54E-04 |
| 51366 | 'UBR5' | -0.417053393 | 0.001034563 | 0.013579536 |
| 51379 | 'CRLF3' | -0.504653127 | 0.002811374 | 0.029681828 |
| 51385 | 'ZNF589' | 0.63261343 | 0.002662786 | 0.028377454 |
| 5139 | 'PDE3A' | -1.370471174 | 6.40E-05 | 0.001514154 |
| 51397 | 'COMMD10' | -0.512898005 | 5.43E-04 | 0.008247888 |
| 5141 | 'PDE4A' | -0.650544176 | 1.13E-05 | 3.60E-04 |
| 51421 | 'AMOTL2' | -0.571141092 | 8.05E-13 | 1.49E-10 |
| 51435 | 'SCARA3' | 0.670916595 | 1.68E-05 | 4.99E-04 |
| 51449 | 'PCYOX1' | 0.447360126 | 4.78E-07 | 2.49E-05 |
| 51465 | 'UBE2J1' | -0.645386863 | 9.69E-13 | 1.77E-10 |
| 5147 | 'PDE6D' | -0.489882633 | 0.001905552 | 0.021959711 |
| 51479 | 'ANKFY1' | -0.226722823 | 0.005050641 | 0.046632698 |
| 51495 | 'HACD3' | -0.310743607 | 0.001004419 | 0.013293955 |
| 51497 | 'NELFCD' | 0.552040662 | 6.29E-06 | 2.16E-04 |
| 51499 | 'TRIAP1' | -0.396904689 | 0.004207928 | 0.040205932 |
| 51506 | 'UFC1' | -0.482452372 | 1.48E-04 | 0.002983778 |
| 51513 | 'ETV7' | -1.504003771 | 3.27E-04 | 0.005503533 |
| 51528 | 'JKAMP' | -0.468297302 | 4.13E-06 | 1.52E-04 |
| 5154 | 'PDGFA' | -0.744851709 | 0.00213656 | 0.023987613 |
| 51542 | 'VPS54' | -0.599715959 | 4.32E-06 | 1.57E-04 |
| 5156 | 'PDGFRA' | 0.437665363 | 1.18E-04 | 0.002499864 |
| 5157 | 'PDGFRL' | 2.482913463 | 7.44E-05 | 0.001715464 |
| 51596 | 'CUTA' | -0.431422147 | 1.71E-05 | 5.06E-04 |
| 516 | 'ATP5MC1' | -0.509222628 | 0.00203755 | 0.023120345 |
| 51619 | 'UBE2D4' | 0.444423153 | 9.05E-04 | 0.012368542 |
| 51655 | 'RASD1' | 0.978793637 | 0.00113446 | 0.014608752 |
| 5166 | 'PDK4' | 1.704569788 | 1.56E-05 | 4.74E-04 |
| 51661 | 'FKBP7' | 0.666544854 | 7.44E-05 | 0.001715464 |
| 51669 | 'SARAF' | 0.309881606 | 1.92E-04 | 0.003694098 |
| 51676 | 'ASB2' | -2.596261996 | 0.001500881 | 0.018113451 |
| 5168 | 'ENPP2' | -0.58639082 | 4.20E-04 | 0.00670457 |
| 517 | 'ATP5MC2' | -0.32704121 | 9.80E-04 | 0.013070946 |
| 51701 | 'NLK' | 0.48858443 | 0.005226797 | 0.047817599 |
| 51715 | 'RAB23' | -0.544809948 | 9.69E-04 | 0.012962977 |
| 51734 | 'MSRB1' | -0.552829309 | 1.86E-04 | 0.003618553 |
| 51759 | 'C9orf78' | 0.320459894 | 0.001965202 | 0.02246436 |
| 51765 | 'STK26' | -0.633100357 | 1.02E-04 | 0.002213147 |
| 5178 | 'PEG3' | 0.909763753 | 3.72E-04 | 0.006097146 |
| 5211 | 'PFKL' | 0.289254272 | 0.001705929 | 0.020067387 |
| 5216 | 'PFN1' | -0.284048739 | 3.93E-05 | 0.001017089 |
| 5217 | 'PFN2' | -1.156190043 | 1.60E-16 | 4.94E-14 |
| 5250 | 'SLC25A3' | -0.217823145 | 8.07E-04 | 0.011327475 |
| 5251 | 'PHEX' | 1.902153745 | 4.29E-04 | 0.006795926 |
| 5270 | 'SERPINE2' | 0.961606276 | 0.003200725 | 0.0325589 |
| 5286 | 'PIK3C2A' | -0.622689247 | 3.24E-12 | 5.46E-10 |
| 5289 | 'PIK3C3' | 0.348102891 | 5.52E-04 | 0.008355585 |
| 529 | 'ATP6V1E1' | -0.315700119 | 2.48E-04 | 0.004484195 |
| 5304 | 'PIP' | 2.576640993 | 5.03E-04 | 0.007729128 |
| 5308 | 'PITX2' | -0.832535775 | 2.35E-04 | 0.004295252 |
| 5321 | 'PLA2G4A' | 0.652309744 | 5.92E-04 | 0.008821112 |
| 5328 | 'PLAU' | 0.944599332 | 3.16E-06 | 1.21E-04 |
| 534 | 'ATP6V1G2' | -2.551569486 | 2.08E-04 | 0.003930532 |
| 5354 | 'PLP1' | -2.473505277 | 1.19E-04 | 0.002499864 |
| 5376 | 'PMP22' | -0.812346466 | 1.52E-19 | 7.38E-17 |
| 53838 | 'C11orf24' | -0.478767938 | 5.98E-07 | 3.00E-05 |
| 5393 | 'EXOSC9' | -0.550410479 | 1.46E-05 | 4.48E-04 |
| 54112 | 'GPR88' | 2.818809201 | 0.004829902 | 0.044971063 |
| 5413 | 'SEPTIN5' | -1.33120284 | 3.02E-09 | 2.85E-07 |
| 5430 | 'POLR2A' | -0.275685213 | 0.002352179 | 0.0258953 |
| 5433 | 'POLR2D' | 0.506189485 | 1.03E-05 | 3.35E-04 |
| 5440 | 'POLR2K' | -0.438723529 | 5.93E-06 | 2.06E-04 |
| 54414 | 'SIAE' | 0.465853821 | 4.72E-05 | 0.001166668 |
| 54431 | 'DNAJC10' | 0.568756696 | 2.03E-06 | 8.35E-05 |
| 5445 | 'PON2' | -0.314325275 | 7.99E-04 | 0.011267936 |
| 54453 | 'RIN2' | 0.776459787 | 9.59E-05 | 0.002114296 |
| 54458 | 'PRR13' | -0.495666902 | 4.57E-10 | 5.01E-08 |
| 54462 | 'CCSER2' | -0.377662867 | 6.66E-04 | 0.009690644 |
| 54520 | 'CCDC93' | 0.423089666 | 2.08E-05 | 5.97E-04 |
| 54534 | 'MRPL50' | -0.502379404 | 0.003754245 | 0.0367596 |
| 54537 | 'SHLD2' | 0.767054735 | 4.43E-08 | 3.20E-06 |
| 54540 | 'FAM193B' | 0.57446397 | 7.58E-05 | 0.001742236 |
| 54541 | 'DDIT4' | 0.995170528 | 1.04E-04 | 0.002256708 |
| 54542 | 'RC3H2' | -0.694672508 | 1.86E-09 | 1.87E-07 |
| 54585 | 'LZTFL1' | 0.370749656 | 0.003506622 | 0.03500033 |
| 54602 | 'NDFIP2' | -0.458398459 | 5.13E-04 | 0.007864351 |
| 54627 | 'MAP10' | 0.864170184 | 0.001132992 | 0.014608752 |
| 54629 | 'MINDY2' | -0.997047144 | 8.63E-05 | 0.001936892 |
| 5465 | 'PPARA' | 0.535859037 | 1.39E-04 | 0.00284789 |
| 54681 | 'P4HTM' | 0.734289406 | 4.40E-11 | 5.89E-09 |
| 54704 | 'PDP1' | 0.73562145 | 3.31E-11 | 4.65E-09 |
| 54741 | 'LEPROT' | -0.295204352 | 3.18E-04 | 0.005390179 |
| 54751 | 'FBLIM1' | -0.960957759 | 1.63E-15 | 4.47E-13 |
| 5479 | 'PPIB' | 0.422107909 | 3.13E-06 | 1.20E-04 |
| 54790 | 'TET2' | -0.643206949 | 1.28E-05 | 3.99E-04 |
| 54800 | 'KLHL24' | -0.347406098 | 0.003256272 | 0.032907047 |
| 5481 | 'PPID' | 0.421956035 | 6.07E-05 | 0.001444827 |
| 54811 | 'ZNF562' | 0.353324393 | 0.002477921 | 0.026947983 |
| 54843 | 'SYTL2' | -2.489028266 | 3.15E-07 | 1.75E-05 |
| 54876 | 'DCAF16' | -0.556919709 | 1.91E-04 | 0.003683923 |
| 54877 | 'ZCCHC2' | 1.216433972 | 8.50E-05 | 0.001910769 |
| 54878 | 'DPP8' | -0.384446525 | 4.49E-04 | 0.007036712 |
| 54880 | 'BCOR' | -0.529991246 | 1.03E-04 | 0.002232724 |
| 54883 | 'CWC25' | 0.793237358 | 8.11E-10 | 8.56E-08 |
| 54893 | 'MTMR10' | -0.473440826 | 0.002241924 | 0.024907338 |
| 54915 | 'YTHDF1' | 0.346327313 | 2.02E-04 | 0.003823726 |
| 5493 | 'PPL' | 1.5647167 | 0.004617081 | 0.043216489 |
| 54934 | 'KANSL2' | 0.559868656 | 3.76E-04 | 0.00614054 |
| 54935 | 'DUSP23' | 0.84075815 | 5.56E-04 | 0.008406246 |
| 54940 | 'OCIAD1' | -0.392234805 | 1.64E-06 | 6.92E-05 |
| 55 | 'ACP3' | 2.584015758 | 0.003946673 | 0.038268636 |
| 55002 | 'TMCO3' | 0.286819443 | 0.004027944 | 0.038813483 |
| 55008 | 'HERC6' | -1.046308714 | 4.23E-04 | 0.00673894 |
| 55012 | 'PPP2R3C' | -0.378505642 | 0.003718322 | 0.036562859 |
| 55026 | 'TMEM255A' | -2.322182193 | 7.38E-06 | 2.51E-04 |
| 55041 | 'PLEKHB2' | -0.689874783 | 1.20E-10 | 1.49E-08 |
| 55055 | 'ZWILCH' | 0.612667884 | 4.70E-04 | 0.007306061 |
| 55075 | 'UACA' | 0.361717508 | 0.004577123 | 0.042913365 |
| 55081 | 'IFT57' | 0.443699889 | 9.26E-04 | 0.012567284 |
| 55083 | 'KIF26B' | -1.722621898 | 2.79E-07 | 1.58E-05 |
| 55084 | 'SOBP' | 0.666899583 | 0.002575431 | 0.02765456 |
| 55093 | 'NTAQ1' | -0.876286583 | 0.001072128 | 0.014007717 |
| 55102 | 'ATG2B' | 0.30444977 | 0.002065863 | 0.02339165 |
| 55108 | 'BSDC1' | -0.36400657 | 9.65E-04 | 0.012928338 |
| 55117 | 'SLC6A15' | 1.534589453 | 1.15E-08 | 9.51E-07 |
| 5514 | 'PPP1R10' | 0.307105589 | 0.001678016 | 0.01981717 |
| 55147 | 'RBM23' | -0.704459787 | 3.30E-09 | 3.08E-07 |
| 55153 | 'SDAD1' | 0.344641439 | 1.81E-04 | 0.003524568 |
| 55161 | 'TMEM33' | 0.709307016 | 7.82E-09 | 6.78E-07 |
| 55174 | 'INTS10' | 0.482056405 | 4.64E-05 | 0.00115334 |
| 55177 | 'RMDN3' | 0.356753639 | 0.00113554 | 0.014611591 |
| 55198 | 'APPL2' | -0.643902894 | 2.34E-05 | 6.57E-04 |
| 55226 | 'NAT10' | 0.413642107 | 9.28E-04 | 0.012574324 |
| 55228 | 'PNMA8A' | 1.329438478 | 0.004060701 | 0.039040611 |
| 5524 | 'PTPA' | -0.505167056 | 7.73E-06 | 2.61E-04 |
| 55243 | 'KIRREL1' | -0.671687265 | 6.05E-22 | 3.81E-19 |
| 55246 | 'CCDC25' | 0.397233819 | 4.37E-04 | 0.006894239 |
| 55252 | 'ASXL2' | -0.321742683 | 0.002009127 | 0.022889503 |
| 55257 | 'MRGBP' | 0.496697981 | 0.001678828 | 0.01981717 |
| 55266 | 'TMEM19' | -0.39786323 | 3.28E-04 | 0.00551238 |
| 55275 | 'VPS53' | 0.30109746 | 0.002445181 | 0.026677267 |
| 55288 | 'RHOT1' | -0.58562272 | 3.67E-07 | 1.98E-05 |
| 552891 | 'DNAJC25-GNG10' | -4.881697311 | 0.005487062 | 0.049611216 |
| 55299 | 'BRIX1' | -1.122242704 | 1.36E-05 | 4.19E-04 |
| 55301 | 'OLAH' | 1.413762488 | 0.002918226 | 0.030581997 |
| 55323 | 'LARP6' | 0.377734849 | 2.15E-04 | 0.004015408 |
| 55324 | 'ABCF3' | 0.372400632 | 0.003841069 | 0.037458687 |
| 55328 | 'RNLS' | -0.473993464 | 0.003468219 | 0.034678109 |
| 5537 | 'PPP6C' | -0.516945566 | 2.25E-07 | 1.29E-05 |
| 55450 | 'CAMK2N1' | 0.815274413 | 1.28E-05 | 3.99E-04 |
| 55471 | 'NDUFAF7' | 0.407975571 | 0.002390561 | 0.026254581 |
| 55500 | 'ETNK1' | -0.417054535 | 0.001413155 | 0.017343542 |
| 55502 | 'HES6' | -1.539866046 | 0.004469512 | 0.042160249 |
| 55556 | 'ENOSF1' | 0.770190681 | 2.96E-06 | 1.16E-04 |
| 55585 | 'UBE2Q1' | -0.642402929 | 5.23E-16 | 1.51E-13 |
| 55588 | 'MED29' | -0.383738286 | 0.001774445 | 0.020729911 |
| 55614 | 'KIF16B' | -0.793967629 | 1.00E-07 | 6.51E-06 |
| 5562 | 'PRKAA1' | -0.281790538 | 8.74E-04 | 0.01205476 |
| 55621 | 'TRMT1' | 0.429588193 | 0.002529545 | 0.027334527 |
| 55632 | 'G2E3' | -0.515028351 | 0.001124403 | 0.014545354 |
| 5565 | 'PRKAB2' | -0.830488582 | 6.75E-08 | 4.61E-06 |
| 55654 | 'TMEM127' | 0.372061581 | 1.58E-04 | 0.003144712 |
| 55669 | 'MFN1' | 0.411218678 | 7.37E-05 | 0.001704393 |
| 5567 | 'PRKACB' | -0.303126442 | 0.002548135 | 0.027465562 |
| 55670 | 'PEX26' | -0.603561499 | 3.57E-08 | 2.69E-06 |
| 55679 | 'LIMS2' | -1.105967615 | 0.001671778 | 0.019761387 |
| 55692 | 'LUC7L' | 0.639029609 | 2.93E-09 | 2.80E-07 |
| 55729 | 'ATF7IP' | 0.306764581 | 5.29E-04 | 0.008086775 |
| 55742 | 'PARVA' | -0.346075693 | 7.53E-05 | 0.001734738 |
| 55751 | 'TMEM184C' | -0.292861271 | 0.002328619 | 0.025735936 |
| 55752 | 'SEPTIN11' | -0.740951168 | 1.32E-04 | 0.002735976 |
| 55755 | 'CDK5RAP2' | 0.545959822 | 6.08E-04 | 0.008996656 |
| 5576 | 'PRKAR2A' | 0.754275804 | 8.88E-16 | 2.47E-13 |
| 55760 | 'DHX32' | 0.378621118 | 3.04E-04 | 0.005226921 |
| 55764 | 'IFT122' | 0.668932867 | 1.02E-07 | 6.56E-06 |
| 55770 | 'EXOC2' | 0.528274336 | 1.35E-06 | 5.93E-05 |
| 55775 | 'TDP1' | 0.429983624 | 0.004453177 | 0.042029483 |
| 55777 | 'MBD5' | -0.356563974 | 0.005152547 | 0.047300512 |
| 55790 | 'CSGALNACT1' | 1.07871073 | 0.002144245 | 0.024058003 |
| 558 | 'AXL' | 0.609565697 | 2.09E-20 | 1.18E-17 |
| 55827 | 'DCAF6' | 0.542795472 | 2.24E-09 | 2.22E-07 |
| 55862 | 'ECHDC1' | -0.330256353 | 0.003985509 | 0.038481949 |
| 55898 | 'UNC45A' | 0.372056883 | 3.08E-06 | 1.19E-04 |
| 55900 | 'ZNF302' | 0.366211457 | 0.004350753 | 0.041292071 |
| 55915 | 'LANCL2' | -0.456745807 | 0.002845624 | 0.030024773 |
| 55920 | 'RCC2' | 0.861139503 | 1.37E-10 | 1.68E-08 |
| 55959 | 'SULF2' | -0.75237288 | 4.52E-05 | 0.001131954 |
| 56005 | 'MYDGF' | 0.357400315 | 5.88E-04 | 0.008785639 |
| 5604 | 'MAP2K1' | 0.282945764 | 0.001325595 | 0.016531524 |
| 5606 | 'MAP2K3' | -0.618639144 | 2.28E-04 | 0.004203776 |
| 5609 | 'MAP2K7' | 0.571788832 | 7.35E-08 | 4.94E-06 |
| 56101 | 'PCDHGB5' | 1.104926029 | 1.93E-23 | 1.43E-20 |
| 56104 | 'PCDHGB1' | 0.817149573 | 0.001230409 | 0.015642853 |
| 56106 | 'PCDHGA10' | -0.906121434 | 0.002885861 | 0.030373912 |
| 56111 | 'PCDHGA4' | 1.567845723 | 2.73E-06 | 1.08E-04 |
| 56112 | 'PCDHGA3' | 0.867440695 | 0.002397976 | 0.026280331 |
| 56113 | 'PCDHGA2' | 0.816728446 | 2.79E-06 | 1.10E-04 |
| 56241 | 'SUSD2' | -1.373051805 | 0.001827627 | 0.021234453 |
| 56253 | 'CRTAM' | 4.585226837 | 8.92E-05 | 0.001993356 |
| 56255 | 'TMX4' | 0.450670852 | 2.06E-05 | 5.92E-04 |
| 56262 | 'LRRC8A' | -0.446661442 | 2.79E-05 | 7.65E-04 |
| 5636 | 'PRPSAP2' | -0.6726897 | 0.003418792 | 0.034275724 |
| 5638 | 'PRRG1' | -0.656826279 | 0.001593641 | 0.018990124 |
| 56548 | 'CHST7' | 0.625521374 | 0.002048183 | 0.023210005 |
| 56606 | 'SLC2A9' | -0.956913486 | 1.08E-05 | 3.45E-04 |
| 56616 | 'DIABLO' | -0.504522913 | 1.08E-04 | 0.002320338 |
| 56654 | 'NPDC1' | 0.751684261 | 7.08E-09 | 6.30E-07 |
| 56674 | 'TMEM9B' | 0.446748276 | 8.66E-07 | 4.03E-05 |
| 56851 | 'EMC7' | 0.55458942 | 8.58E-10 | 9.00E-08 |
| 56897 | 'WRNIP1' | -0.503309068 | 4.90E-04 | 0.007568624 |
| 56898 | 'BDH2' | 0.703434614 | 0.001221977 | 0.015558362 |
| 56901 | 'NDUFA4L2' | -1.678710741 | 0.00534332 | 0.048466252 |
| 56907 | 'SPIRE1' | 0.352871898 | 7.78E-04 | 0.011017471 |
| 56916 | 'SMARCAD1' | 0.322563403 | 3.13E-04 | 0.005347502 |
| 5693 | 'PSMB5' | -0.287782887 | 9.88E-04 | 0.013147882 |
| 56935 | 'SMCO4' | -0.731842212 | 0.002594277 | 0.027804236 |
| 56944 | 'OLFML3' | -0.995552405 | 0.003079137 | 0.031733541 |
| 56954 | 'NIT2' | 0.370852676 | 0.001305281 | 0.016338123 |
| 5696 | 'PSMB8' | 0.387620872 | 0.002990586 | 0.03109112 |
| 56975 | 'FAM20C' | 1.50881479 | 2.06E-06 | 8.45E-05 |
| 56987 | 'BBX' | -0.475069725 | 2.58E-05 | 7.12E-04 |
| 56999 | 'ADAMTS9' | -1.493923375 | 1.03E-10 | 1.30E-08 |
| 57016 | 'AKR1B10' | 2.266730401 | 0.005234328 | 0.047860739 |
| 57026 | 'PDXP' | 0.745848825 | 0.004248028 | 0.04052075 |
| 57035 | 'RSRP1' | 0.51448776 | 1.27E-06 | 5.61E-05 |
| 57048 | 'PLSCR3' | -0.362091361 | 4.18E-04 | 0.006680436 |
| 57095 | 'PITHD1' | 0.521791475 | 2.16E-05 | 6.17E-04 |
| 57097 | 'PARP11' | -0.560563164 | 0.002337178 | 0.025796977 |
| 571 | 'BACH1' | 0.486587252 | 9.78E-04 | 0.013066128 |
| 57107 | 'PDSS2' | 0.538292587 | 0.001020146 | 0.013431788 |
| 57140 | 'RNPEPL1' | 0.545652015 | 2.53E-05 | 7.02E-04 |
| 57153 | 'SLC44A2' | -0.433508649 | 0.002948587 | 0.030808631 |
| 57158 | 'JPH2' | -1.770410161 | 6.36E-07 | 3.14E-05 |
| 5716 | 'PSMD10' | -0.879810088 | 6.86E-08 | 4.66E-06 |
| 57161 | 'PELI2' | 0.985211392 | 4.40E-04 | 0.006932224 |
| 57169 | 'ZNFX1' | -0.236190922 | 0.002948914 | 0.030808631 |
| 57179 | 'KIAA1191' | -1.135771108 | 1.03E-22 | 7.30E-20 |
| 57184 | 'FAM219B' | 0.399762706 | 5.11E-05 | 0.001251056 |
| 57192 | 'MCOLN1' | -0.526166339 | 4.68E-08 | 3.37E-06 |
| 57194 | 'ATP10A' | -0.903408637 | 0.004118613 | 0.039485718 |
| 57198 | 'ATP8B2' | -0.771078515 | 1.71E-08 | 1.36E-06 |
| 57205 | 'ATP10D' | -0.46891322 | 0.004755493 | 0.044365458 |
| 57214 | 'CEMIP' | -0.930361299 | 1.57E-04 | 0.003126711 |
| 57217 | 'TTC7A' | 0.416416724 | 1.66E-04 | 0.003280633 |
| 57222 | 'ERGIC1' | 0.462245085 | 1.71E-04 | 0.003351327 |
| 57326 | 'PBXIP1' | 0.714344809 | 2.91E-05 | 7.90E-04 |
| 57335 | 'ZNF286A' | 0.388879945 | 0.001817063 | 0.021126154 |
| 57381 | 'RHOJ' | -0.94921732 | 0.001355917 | 0.016817326 |
| 5742 | 'PTGS1' | 1.789808055 | 1.41E-06 | 6.11E-05 |
| 57446 | 'NDRG3' | -0.402649002 | 0.001810786 | 0.021067585 |
| 5745 | 'PTH1R' | -3.199466892 | 0.002438933 | 0.026626196 |
| 57472 | 'CNOT6' | 0.408110082 | 0.001514821 | 0.018238787 |
| 57480 | 'PLEKHG1' | -0.973573133 | 0.003779736 | 0.03694534 |
| 57484 | 'RNF150' | -0.982327027 | 1.85E-04 | 0.003596987 |
| 57522 | 'SRGAP1' | 0.530613935 | 1.22E-05 | 3.83E-04 |
| 57531 | 'HACE1' | -0.394298301 | 0.005197311 | 0.047616896 |
| 57538 | 'ALPK3' | 1.194816427 | 0.002647319 | 0.028287568 |
| 5754 | 'PTK7' | -0.714911026 | 1.78E-05 | 5.24E-04 |
| 57542 | 'KLHL42' | -0.433630452 | 3.56E-05 | 9.35E-04 |
| 57551 | 'TAOK1' | -0.328229463 | 8.13E-04 | 0.011361058 |
| 57555 | 'NLGN2' | -0.401449079 | 7.90E-04 | 0.011151888 |
| 5756 | 'TWF1' | -0.863098833 | 5.37E-24 | 4.34E-21 |
| 57561 | 'ARRDC3' | 1.365133022 | 1.53E-23 | 1.18E-20 |
| 57563 | 'KLHL8' | -0.793465331 | 1.96E-06 | 8.10E-05 |
| 57590 | 'WDFY1' | -0.505165938 | 0.002005488 | 0.022863369 |
| 576 | 'ADGRB2' | -0.94814326 | 3.95E-08 | 2.93E-06 |
| 57619 | 'SHROOM3' | -1.383658975 | 7.86E-11 | 1.02E-08 |
| 57647 | 'DHX37' | 0.381800691 | 0.005032606 | 0.046542024 |
| 57659 | 'ZBTB4' | -0.467325249 | 2.67E-06 | 1.07E-04 |
| 57664 | 'PLEKHA4' | 0.541973933 | 0.003853342 | 0.037535301 |
| 57693 | 'ZNF317' | 0.358142367 | 0.001808454 | 0.021054859 |
| 57698 | 'SHTN1' | -0.779437111 | 1.47E-04 | 0.002972232 |
| 57717 | 'PCDHB16' | 0.793622924 | 0.001957987 | 0.022425256 |
| 57732 | 'ZFYVE28' | 0.581237543 | 3.56E-05 | 9.35E-04 |
| 5775 | 'PTPN4' | 0.419932277 | 0.005199256 | 0.047616896 |
| 57794 | 'SUGP1' | 0.342926221 | 0.005097663 | 0.046990277 |
| 5780 | 'PTPN9' | -0.409939885 | 8.18E-04 | 0.011400898 |
| 57804 | 'POLD4' | -0.486336495 | 8.48E-06 | 2.84E-04 |
| 5784 | 'PTPN14' | -0.522796622 | 8.00E-04 | 0.011268586 |
| 5796 | 'PTPRK' | -0.335980629 | 0.003603556 | 0.03571618 |
| 5810 | 'RAD1' | -0.497962367 | 1.65E-04 | 0.003258341 |
| 58189 | 'WFDC1' | -2.294386722 | 4.89E-06 | 1.75E-04 |
| 5819 | 'NECTIN2' | -0.459098068 | 0.004860801 | 0.045199066 |
| 58190 | 'CTDSP1' | -0.344347497 | 0.002912431 | 0.030581997 |
| 58191 | 'CXCL16' | -1.217941373 | 0.003080383 | 0.031733541 |
| 5824 | 'PEX19' | -0.337256226 | 0.004832517 | 0.044971063 |
| 5829 | 'PXN' | 0.343920029 | 1.69E-04 | 0.003332369 |
| 58487 | 'CREBZF' | 0.444094553 | 9.92E-06 | 3.25E-04 |
| 58490 | 'RPRD1B' | -0.325912348 | 0.004215993 | 0.040260368 |
| 58499 | 'ZNF462' | -0.911687462 | 2.45E-08 | 1.92E-06 |
| 58504 | 'ARHGAP22' | 0.644211355 | 2.64E-04 | 0.004702367 |
| 58527 | 'ABRACL' | 0.839079465 | 1.06E-06 | 4.80E-05 |
| 5886 | 'RAD23A' | 0.490244119 | 5.71E-05 | 0.001373027 |
| 5892 | 'RAD51D' | -0.504139907 | 0.002172476 | 0.024278603 |
| 5894 | 'RAF1' | -0.243306801 | 0.001254109 | 0.015872927 |
| 59 | 'ACTA2' | -1.934621592 | 1.62E-06 | 6.87E-05 |
| 5908 | 'RAP1B' | -0.697956104 | 3.61E-04 | 0.005956788 |
| 5918 | 'RARRES1' | 0.963249812 | 0.002427923 | 0.02656919 |
| 5919 | 'RARRES2' | -1.082772886 | 2.03E-06 | 8.35E-05 |
| 5920 | 'PLAAT4' | -0.681101491 | 9.30E-04 | 0.012591606 |
| 5922 | 'RASA2' | 0.524121362 | 6.31E-07 | 3.13E-05 |
| 5924 | 'RASGRF2' | -1.278510776 | 9.84E-04 | 0.013103278 |
| 5925 | 'RB1' | -0.333605066 | 0.001173739 | 0.0150235 |
| 5932 | 'RBBP8' | -0.448212275 | 8.58E-04 | 0.011874718 |
| 59338 | 'PLEKHA1' | -0.41973588 | 0.001524433 | 0.018312583 |
| 59339 | 'PLEKHA2' | 0.618457054 | 9.34E-04 | 0.012611172 |
| 59341 | 'TRPV4' | -1.299364376 | 1.56E-09 | 1.60E-07 |
| 59350 | 'RXFP1' | 1.257771949 | 0.003089306 | 0.031786938 |
| 5950 | 'RBP4' | 0.924685724 | 0.00212159 | 0.023866831 |
| 5965 | 'RECQL' | 0.364377551 | 3.61E-04 | 0.005956788 |
| 5980 | 'REV3L' | -0.675112715 | 1.10E-10 | 1.38E-08 |
| 599 | 'BCL2L2' | -0.480219818 | 6.90E-04 | 0.009953088 |
| 5993 | 'RFX5' | -0.36927168 | 1.78E-04 | 0.003469096 |
| 5997 | 'RGS2' | 0.739622068 | 0.005092873 | 0.046971594 |
| 5999 | 'RGS4' | 0.966815135 | 4.11E-06 | 1.51E-04 |
| 60 | 'ACTB' | -0.536465258 | 8.42E-05 | 0.001898373 |
| 6004 | 'RGS16' | -2.755657157 | 0.003176304 | 0.032375685 |
| 6015 | 'RING1' | -0.428546822 | 2.52E-05 | 7.00E-04 |
| 6016 | 'RIT1' | 0.388257817 | 0.001895156 | 0.021880662 |
| 6038 | 'RNASE4' | 0.763213369 | 5.13E-07 | 2.63E-05 |
| 6041 | 'RNASEL' | -0.45798551 | 0.003623058 | 0.035825906 |
| 60412 | 'EXOC4' | -0.298865514 | 0.001357971 | 0.016817326 |
| 6046 | 'BRD2' | -0.427111185 | 2.55E-06 | 1.02E-04 |
| 60481 | 'ELOVL5' | -0.657934938 | 1.65E-21 | 1.00E-18 |
| 60489 | 'APOBEC3G' | -0.810665073 | 0.003156403 | 0.032278564 |
| 60684 | 'TRAPPC11' | -0.538265424 | 1.09E-07 | 6.96E-06 |
| 60685 | 'ZFAND3' | -0.288527063 | 1.00E-04 | 0.002197037 |
| 6134 | 'RPL10' | -0.295965258 | 9.08E-04 | 0.012369632 |
| 6136 | 'RPL12' | -0.337423951 | 1.08E-04 | 0.002320338 |
| 6141 | 'RPL18' | -0.280872998 | 9.03E-04 | 0.012351643 |
| 6143 | 'RPL19' | -0.28412958 | 2.11E-04 | 0.003965242 |
| 6144 | 'RPL21' | -0.311610939 | 1.71E-05 | 5.05E-04 |
| 6160 | 'RPL31' | -0.265567618 | 4.26E-05 | 0.001075421 |
| 6165 | 'RPL35A' | -0.219119677 | 0.002430105 | 0.02656919 |
| 6166 | 'RPL36AL' | -0.235841658 | 0.005354699 | 0.048517682 |
| 6169 | 'RPL38' | -0.317880482 | 0.001476728 | 0.017916793 |
| 6171 | 'RPL41' | -0.224279078 | 3.31E-04 | 0.005554097 |
| 6185 | 'RPN2' | -0.717006398 | 8.04E-14 | 1.75E-11 |
| 6204 | 'RPS10' | -0.516514127 | 1.64E-09 | 1.65E-07 |
| 6205 | 'RPS11' | -0.306145 | 4.60E-05 | 0.001148127 |
| 6232 | 'RPS27' | -0.46453611 | 1.72E-07 | 1.03E-05 |
| 6251 | 'RSU1' | -0.330610523 | 2.43E-04 | 0.004428324 |
| 6259 | 'RYK' | -0.328585983 | 3.10E-04 | 0.005303318 |
| 6272 | 'SORT1' | -0.52525168 | 1.71E-08 | 1.36E-06 |
| 6277 | 'S100A6' | 0.297266164 | 0.002189637 | 0.024422213 |
| 6303 | 'SAT1' | 1.034929004 | 4.82E-07 | 2.50E-05 |
| 6305 | 'SBF1' | 0.289321659 | 0.002914683 | 0.030581997 |
| 6310 | 'ATXN1' | -0.553836226 | 7.26E-05 | 0.001687485 |
| 6319 | 'SCD' | -0.89014969 | 4.01E-11 | 5.50E-09 |
| 633 | 'BGN' | -0.439341259 | 0.003059499 | 0.031595 |
| 635 | 'BHMT' | 2.81610804 | 0.001508397 | 0.018184209 |
| 6373 | 'CXCL11' | -6.087132533 | 9.63E-04 | 0.012908046 |
| 6382 | 'SDC1' | -1.023451412 | 0.004955123 | 0.045950452 |
| 6383 | 'SDC2' | -0.873910784 | 5.22E-04 | 0.00799143 |
| 6386 | 'SDCBP' | -0.443681241 | 1.06E-05 | 3.42E-04 |
| 63874 | 'ABHD4' | -0.891741732 | 9.26E-18 | 3.50E-15 |
| 6388 | 'SDF2' | 0.330433683 | 0.002241787 | 0.024907338 |
| 63893 | 'UBE2O' | 0.681326102 | 1.49E-06 | 6.41E-05 |
| 63895 | 'PIEZO2' | -1.39323654 | 0.00151507 | 0.018238787 |
| 63908 | 'NAPB' | 0.978715288 | 3.98E-06 | 1.47E-04 |
| 63926 | 'ANKEF1' | -0.841170798 | 7.27E-04 | 0.010373382 |
| 63932 | 'CXorf56' | -0.902224325 | 1.16E-08 | 9.54E-07 |
| 63933 | 'MCUR1' | -0.36436059 | 9.89E-04 | 0.013159427 |
| 63934 | 'ZNF667' | 0.802734772 | 4.38E-04 | 0.006910727 |
| 63939 | 'FAM217B' | 0.530126472 | 0.005002294 | 0.046286879 |
| 64061 | 'TSPYL2' | 0.385033042 | 8.93E-04 | 0.012246852 |
| 64081 | 'PBLD' | -0.707673083 | 0.001638421 | 0.019448237 |
| 64115 | 'VSIR' | -0.713685434 | 4.72E-08 | 3.38E-06 |
| 64118 | 'DUS1L' | 0.63249518 | 1.21E-06 | 5.37E-05 |
| 64123 | 'ADGRL4' | 0.694583809 | 0.002647695 | 0.028287568 |
| 64132 | 'XYLT2' | 0.471009854 | 4.46E-05 | 0.001118956 |
| 641339 | 'ZNF674' | 0.943720652 | 0.001050236 | 0.013764 |
| 6418 | 'SET' | 0.2393603 | 4.33E-04 | 0.006833485 |
| 64210 | 'MMS19' | 0.459802509 | 1.89E-06 | 7.85E-05 |
| 64222 | 'TOR3A' | 0.394556178 | 0.004295967 | 0.04093209 |
| 6423 | 'SFRP2' | 1.375959668 | 6.57E-04 | 0.009594642 |
| 64285 | 'RHBDF1' | 0.44441129 | 4.12E-06 | 1.52E-04 |
| 64359 | 'NXN' | -0.87540445 | 6.12E-04 | 0.009034501 |
| 643699 | 'GOLGA8N' | 0.680645368 | 0.003559927 | 0.035428363 |
| 643707 | 'GOLGA6L4' | 0.732076496 | 0.002199173 | 0.024496423 |
| 64374 | 'SIL1' | -0.504453099 | 5.73E-07 | 2.88E-05 |
| 64395 | 'GMCL1' | 0.679342229 | 1.43E-07 | 8.83E-06 |
| 643988 | 'FNDC10' | 1.311319826 | 3.35E-07 | 1.84E-05 |
| 64418 | 'TMEM168' | -0.820498534 | 5.70E-08 | 3.97E-06 |
| 64426 | 'SUDS3' | 0.307599712 | 0.002574142 | 0.02765456 |
| 6443 | 'SGCB' | -0.501669223 | 4.29E-07 | 2.27E-05 |
| 6453 | 'ITSN1' | -0.501070278 | 4.79E-08 | 3.42E-06 |
| 6455 | 'SH3GL1' | -0.326900009 | 5.22E-04 | 0.00799143 |
| 64598 | 'MOSPD3' | -0.555859533 | 7.69E-05 | 0.001759264 |
| 64599 | 'GIGYF1' | 0.605357774 | 5.96E-08 | 4.13E-06 |
| 646851 | 'FAM227A' | 1.039530964 | 2.73E-04 | 0.004840615 |
| 64689 | 'GORASP1' | -0.257123566 | 0.005537274 | 0.049927115 |
| 64753 | 'CCDC136' | -1.127941165 | 9.80E-04 | 0.013070946 |
| 64760 | 'FAM160B2' | 0.273631871 | 0.003614685 | 0.035784749 |
| 64762 | 'GAREM1' | 0.572263478 | 3.14E-04 | 0.005354657 |
| 64766 | 'S100PBP' | -0.402230812 | 0.00156921 | 0.018757687 |
| 64771 | 'ILRUN' | 0.266010709 | 0.002163778 | 0.024213227 |
| 64773 | 'PCED1A' | -0.593370449 | 4.03E-04 | 0.006523413 |
| 64780 | 'MICAL1' | -0.64148477 | 0.0018419 | 0.021371073 |
| 648 | 'BMI1' | -0.505542293 | 0.003049705 | 0.031532172 |
| 64838 | 'FNDC4' | 0.361774466 | 0.001860142 | 0.021553298 |
| 64849 | 'SLC13A3' | 1.430295654 | 1.96E-04 | 0.003752662 |
| 64854 | 'USP46' | -0.918914434 | 6.40E-12 | 1.04E-09 |
| 64855 | 'NIBAN2' | 0.488077749 | 1.15E-11 | 1.72E-09 |
| 64856 | 'VWA1' | -1.616792724 | 6.02E-04 | 0.008942168 |
| 64859 | 'NABP1' | -0.683429649 | 2.50E-04 | 0.004504539 |
| 64866 | 'CDCP1' | 1.267004938 | 4.00E-10 | 4.56E-08 |
| 6489 | 'ST8SIA1' | 1.787721882 | 7.61E-04 | 0.010786071 |
| 6499 | 'SKIV2L' | 0.311917361 | 0.001257153 | 0.015899619 |
| 65010 | 'SLC26A6' | 0.545515638 | 1.01E-05 | 3.30E-04 |
| 65018 | 'PINK1' | 0.356851946 | 4.23E-05 | 0.001071732 |
| 6502 | 'SKP2' | -1.006424888 | 3.67E-04 | 0.006033553 |
| 65082 | 'VPS33A' | 0.419460952 | 5.83E-05 | 0.001399105 |
| 65083 | 'NOL6' | 0.446612919 | 9.75E-06 | 3.21E-04 |
| 65124 | 'SOWAHC' | -0.774349505 | 2.11E-04 | 0.00396261 |
| 6515 | 'SLC2A3' | -0.609383958 | 1.58E-06 | 6.72E-05 |
| 651746 | 'ANKRD33B' | -1.577654526 | 4.33E-08 | 3.16E-06 |
| 65220 | 'NADK' | 0.279481323 | 0.003373353 | 0.033929148 |
| 653784 | 'MZT2A' | -0.773418726 | 2.19E-06 | 8.87E-05 |
| 6548 | 'SLC9A1' | -0.293425902 | 0.004510318 | 0.042427438 |
| 6575 | 'SLC20A2' | -0.787983453 | 2.90E-05 | 7.88E-04 |
| 6590 | 'SLPI' | 1.993218548 | 0.004566253 | 0.042858734 |
| 65981 | 'CAPRIN2' | 0.414530321 | 1.53E-04 | 0.0030675 |
| 65983 | 'GRAMD2B' | -1.015824901 | 6.92E-07 | 3.38E-05 |
| 65985 | 'AACS' | -0.520814595 | 4.77E-05 | 0.001178466 |
| 6599 | 'SMARCC1' | -0.454529772 | 2.87E-04 | 0.005035923 |
| 65997 | 'RASL11B' | -1.800992452 | 8.71E-04 | 0.01202914 |
| 66004 | 'LYNX1' | -0.522732137 | 0.005539577 | 0.049927115 |
| 66005 | 'CHID1' | 0.284926442 | 0.005149603 | 0.047300512 |
| 66008 | 'TRAK2' | -0.431964729 | 3.76E-04 | 0.006140881 |
| 6604 | 'SMARCD3' | -0.683663121 | 8.84E-11 | 1.13E-08 |
| 6608 | 'SMO' | -0.607975082 | 2.59E-04 | 0.004625762 |
| 661 | 'POLR3D' | -0.490274588 | 7.45E-06 | 2.52E-04 |
| 6612 | 'SUMO3' | 0.349360379 | 1.58E-05 | 4.78E-04 |
| 6619 | 'SNAPC3' | -0.304425325 | 0.002066964 | 0.02339165 |
| 6621 | 'SNAPC4' | 0.583055468 | 1.96E-04 | 0.003744247 |
| 6624 | 'FSCN1' | -0.633806191 | 4.06E-05 | 0.001041916 |
| 663 | 'BNIP2' | -0.337700745 | 1.19E-04 | 0.002499864 |
| 6631 | 'SNRPC' | -0.711526124 | 4.86E-13 | 9.27E-11 |
| 6640 | 'SNTA1' | -0.631545371 | 0.003175777 | 0.032375685 |
| 6645 | 'SNTB2' | -0.90050001 | 3.37E-08 | 2.55E-06 |
| 6660 | 'SOX5' | 1.219298498 | 0.001574607 | 0.018808976 |
| 6666 | 'SOX12' | 0.466061815 | 3.03E-04 | 0.005218593 |
| 6687 | 'SPG7' | 0.353242864 | 3.32E-05 | 8.80E-04 |
| 6695 | 'SPOCK1' | 0.793476499 | 2.41E-09 | 2.34E-07 |
| 6696 | 'SPP1' | 1.175066616 | 1.85E-05 | 5.41E-04 |
| 6711 | 'SPTBN1' | -0.343803775 | 0.003891133 | 0.037838376 |
| 6714 | 'SRC' | -0.442870205 | 2.22E-05 | 6.30E-04 |
| 6722 | 'SRF' | -0.495932321 | 3.04E-06 | 1.18E-04 |
| 6734 | 'SRPRA' | -0.274838332 | 6.82E-05 | 0.001593583 |
| 6741 | 'SSB' | -1.388154826 | 1.41E-26 | 1.50E-23 |
| 6773 | 'STAT2' | -0.581554771 | 3.95E-07 | 2.12E-05 |
| 6777 | 'STAT5B' | -0.272627161 | 0.004035116 | 0.038860566 |
| 6780 | 'STAU1' | -0.455747743 | 2.07E-07 | 1.20E-05 |
| 6792 | 'CDKL5' | -0.812512713 | 2.08E-04 | 0.003930532 |
| 6804 | 'STX1A' | 1.189156227 | 8.61E-07 | 4.02E-05 |
| 6815 | 'STYX' | 0.378600912 | 6.65E-04 | 0.009687474 |
| 682 | 'BSG' | 0.527466927 | 2.63E-16 | 7.84E-14 |
| 6836 | 'SURF4' | 0.403837432 | 7.57E-04 | 0.010745463 |
| 684 | 'BST2' | -1.566166712 | 0.001655269 | 0.019607154 |
| 6844 | 'VAMP2' | -0.349995118 | 0.003077495 | 0.031733541 |
| 6845 | 'VAMP7' | -0.424777282 | 1.40E-04 | 0.002864736 |
| 6863 | 'TAC1' | 2.740437635 | 4.38E-07 | 2.30E-05 |
| 6869 | 'TACR1' | -1.627720543 | 3.09E-06 | 1.19E-04 |
| 6873 | 'TAF2' | -0.295628047 | 0.001128493 | 0.014576083 |
| 6876 | 'TAGLN' | -0.864637604 | 6.94E-08 | 4.70E-06 |
| 6879 | 'TAF7' | 0.482834661 | 3.43E-05 | 9.03E-04 |
| 6880 | 'TAF9' | -0.377986818 | 0.001893204 | 0.021876742 |
| 689 | 'BTF3' | -0.211724857 | 0.00389554 | 0.037859567 |
| 6894 | 'TARBP1' | 0.317983946 | 0.002629012 | 0.028141021 |
| 6917 | 'TCEA1' | -0.320084062 | 2.39E-04 | 0.004365212 |
| 6921 | 'ELOC' | -0.445608981 | 0.00552509 | 0.049901958 |
| 692312 | 'PPAN-P2RY11' | 0.827077051 | 3.17E-04 | 0.005383589 |
| 6929 | 'TCF3' | 0.485447298 | 9.97E-05 | 0.002186431 |
| 6940 | 'ZNF354A' | 0.568033143 | 0.001111371 | 0.014398691 |
| 6949 | 'TCOF1' | 0.533648555 | 3.57E-04 | 0.005902432 |
| 6988 | 'TCTA' | -0.600177431 | 1.01E-06 | 4.59E-05 |
| 7003 | 'TEAD1' | -0.627702818 | 8.90E-07 | 4.13E-05 |
| 7005 | 'TEAD3' | -0.665386459 | 5.56E-06 | 1.96E-04 |
| 7009 | 'TMBIM6' | -0.299658064 | 9.15E-06 | 3.04E-04 |
| 7013 | 'TERF1' | -0.907949034 | 1.47E-13 | 3.00E-11 |
| 7027 | 'TFDP1' | 0.448877826 | 5.48E-08 | 3.85E-06 |
| 7035 | 'TFPI' | 0.953736654 | 7.18E-07 | 3.48E-05 |
| 7041 | 'TGFB1I1' | -1.085454133 | 7.27E-07 | 3.50E-05 |
| 7043 | 'TGFB3' | -0.934497496 | 2.47E-04 | 0.004484195 |
| 7045 | 'TGFBI' | 0.502747071 | 0.005156362 | 0.047300512 |
| 7048 | 'TGFBR2' | 0.712376153 | 1.59E-06 | 6.76E-05 |
| 7049 | 'TGFBR3' | 0.776557914 | 2.06E-05 | 5.92E-04 |
| 7056 | 'THBD' | 3.447458643 | 1.53E-06 | 6.54E-05 |
| 7059 | 'THBS3' | -0.86510519 | 2.13E-09 | 2.12E-07 |
| 7070 | 'THY1' | -0.445211157 | 0.001520939 | 0.018283541 |
| 7076 | 'TIMP1' | 0.626129203 | 9.88E-09 | 8.40E-07 |
| 7079 | 'TIMP4' | 1.234737145 | 1.02E-04 | 0.002226849 |
| 7086 | 'TKT' | 0.310727357 | 0.003751634 | 0.0367596 |
| 7090 | 'TLE3' | 0.456390346 | 0.001752883 | 0.020562803 |
| 7094 | 'TLN1' | -0.22976388 | 0.002397358 | 0.026280331 |
| 7098 | 'TLR3' | -0.997256045 | 7.77E-07 | 3.68E-05 |
| 71 | 'ACTG1' | -0.538935287 | 1.14E-04 | 0.002416575 |
| 7123 | 'CLEC3B' | 0.581408008 | 0.002430585 | 0.02656919 |
| 7150 | 'TOP1' | 0.624736757 | 3.35E-11 | 4.67E-09 |
| 7155 | 'TOP2B' | 0.407681449 | 1.45E-04 | 0.002948308 |
| 7168 | 'TPM1' | -1.062075052 | 4.13E-10 | 4.66E-08 |
| 7169 | 'TPM2' | -0.998491207 | 3.81E-06 | 1.42E-04 |
| 7181 | 'NR2C1' | -0.364428279 | 0.003110104 | 0.031923635 |
| 7184 | 'HSP90B1' | 0.474168109 | 6.41E-04 | 0.009422085 |
| 7188 | 'TRAF5' | -0.625690042 | 0.003831454 | 0.037386373 |
| 72 | 'ACTG2' | -1.604202565 | 1.16E-04 | 0.002463471 |
| 7205 | 'TRIP6' | -0.30011347 | 1.59E-04 | 0.003165438 |
| 7222 | 'TRPC3' | -2.893321829 | 3.01E-04 | 0.005193706 |
| 7227 | 'TRPS1' | 0.470776566 | 0.004260272 | 0.040614748 |
| 723790 | 'H2AC19' | 21.74765312 | 2.62E-08 | 2.04E-06 |
| 726 | 'CAPN5' | -0.848780717 | 6.98E-07 | 3.40E-05 |
| 7275 | 'TUB' | -0.897081087 | 2.73E-06 | 1.08E-04 |
| 728741 | 'NPIPB6' | 0.644870792 | 0.001716975 | 0.020169413 |
| 728841 | 'NBPF8' | 0.452562444 | 0.00275291 | 0.029136966 |
| 729092 | 'AGAP5' | 0.652847416 | 1.84E-04 | 0.00358172 |
| 729852 | 'UMAD1' | -0.771169813 | 4.21E-07 | 2.24E-05 |
| 729920 | 'CRPPA' | -1.214290606 | 4.04E-04 | 0.006524288 |
| 730 | 'C7' | 3.024071872 | 1.14E-12 | 2.06E-10 |
| 730094 | 'MOSMO' | 0.432803984 | 0.003362818 | 0.033863262 |
| 7307 | 'U2AF1' | -0.716987274 | 5.96E-04 | 0.008877001 |
| 731220 | 'RFX8' | 1.400699586 | 7.11E-06 | 2.42E-04 |
| 7318 | 'UBA7' | -0.51297821 | 4.49E-04 | 0.00703571 |
| 7321 | 'UBE2D1' | 0.436238893 | 5.46E-05 | 0.001322772 |
| 7332 | 'UBE2L3' | -0.302579341 | 6.17E-04 | 0.009083383 |
| 7336 | 'UBE2V2' | -0.711964449 | 1.04E-09 | 1.08E-07 |
| 7337 | 'UBE3A' | 0.295933305 | 0.003622911 | 0.035825906 |
| 7345 | 'UCHL1' | 0.531680626 | 1.25E-10 | 1.54E-08 |
| 7360 | 'UGP2' | -0.302963074 | 0.004942306 | 0.045856616 |
| 7376 | 'NR1H2' | 0.319533338 | 0.001960462 | 0.022425256 |
| 7384 | 'UQCRC1' | -0.26224647 | 0.001915551 | 0.022045044 |
| 7385 | 'UQCRC2' | -0.729391909 | 3.30E-13 | 6.44E-11 |
| 7392 | 'USF2' | 0.344612771 | 2.55E-05 | 7.05E-04 |
| 7405 | 'UVRAG' | -0.345608454 | 0.002574692 | 0.02765456 |
| 7407 | 'VARS1' | 0.633615624 | 3.35E-07 | 1.84E-05 |
| 7412 | 'VCAM1' | 0.831092963 | 9.36E-05 | 0.00207533 |
| 7414 | 'VCL' | -1.073376129 | 3.02E-05 | 8.13E-04 |
| 7422 | 'VEGFA' | 0.89127577 | 8.10E-04 | 0.011327475 |
| 7423 | 'VEGFB' | 0.396601315 | 0.005109753 | 0.047076192 |
| 7436 | 'VLDLR' | -1.361502197 | 0.003170704 | 0.032369742 |
| 744 | 'MPPED2' | -1.779221891 | 7.15E-04 | 0.010243558 |
| 7448 | 'VTN' | -3.105697765 | 1.19E-06 | 5.30E-05 |
| 7458 | 'EIF4H' | -0.219434734 | 0.003934631 | 0.038217629 |
| 7525 | 'YES1' | 0.425759932 | 2.47E-04 | 0.004484195 |
| 753 | 'LDLRAD4' | -1.623042323 | 0.003972324 | 0.038461513 |
| 7556 | 'ZNF10' | 0.483264727 | 0.004730959 | 0.044185076 |
| 7569 | 'ZNF182' | 0.679744691 | 8.79E-04 | 0.012115543 |
| 7574 | 'ZNF26' | 0.566341752 | 0.003486424 | 0.03481917 |
| 7581 | 'ZNF33A' | 0.39555698 | 0.001318602 | 0.016456383 |
| 7586 | 'ZKSCAN1' | 0.395046605 | 2.83E-04 | 0.004972235 |
| 7592 | 'ZNF41' | 0.406801093 | 0.003212076 | 0.032635302 |
| 7627 | 'ZNF75A' | 0.748635552 | 2.98E-09 | 2.83E-07 |
| 7637 | 'ZNF84' | 0.863066933 | 3.28E-17 | 1.16E-14 |
| 7675 | 'ZNF121' | 1.136742758 | 1.67E-11 | 2.44E-09 |
| 767558 | 'LUZP6' | -0.526779469 | 3.68E-06 | 1.38E-04 |
| 7692 | 'ZNF133' | 0.780161448 | 1.37E-06 | 5.99E-05 |
| 7694 | 'ZNF135' | 0.722294514 | 0.001501462 | 0.018113451 |
| 7699 | 'ZNF140' | 0.725210991 | 4.96E-08 | 3.51E-06 |
| 773 | 'CACNA1A' | -0.954506309 | 1.12E-04 | 0.002381801 |
| 775 | 'CACNA1C' | -0.5056216 | 0.001299983 | 0.016295808 |
| 7753 | 'ZNF202' | 1.063618053 | 1.40E-12 | 2.48E-10 |
| 7767 | 'ZNF224' | 0.423327524 | 8.17E-04 | 0.011400898 |
| 7770 | 'ZNF227' | 0.570624117 | 7.81E-04 | 0.011030577 |
| 7772 | 'ZNF229' | 0.93491249 | 3.56E-07 | 1.94E-05 |
| 7775 | 'ZNF232' | 0.684775441 | 0.001217815 | 0.01551756 |
| 780 | 'DDR1' | -0.882884965 | 0.002458182 | 0.026784731 |
| 7844 | 'RNF103' | -0.383160698 | 1.95E-04 | 0.003733303 |
| 7867 | 'MAPKAPK3' | 0.447392548 | 5.68E-04 | 0.008556724 |
| 7871 | 'SLMAP' | -0.743090305 | 8.70E-09 | 7.51E-07 |
| 7873 | 'MANF' | 0.402877085 | 0.005470887 | 0.049491292 |
| 79020 | 'C7orf25' | -0.571735646 | 7.41E-06 | 2.51E-04 |
| 79047 | 'KCTD15' | -0.732181682 | 0.003948887 | 0.038268636 |
| 79083 | 'MLPH' | 0.502867688 | 7.58E-04 | 0.010748956 |
| 79109 | 'MAPKAP1' | 0.297661903 | 0.001017052 | 0.01340144 |
| 7913 | 'DEK' | -0.42606325 | 1.20E-06 | 5.33E-05 |
| 79134 | 'TMEM185B' | -0.291402588 | 0.001367663 | 0.016895007 |
| 7916 | 'PRRC2A' | -0.464981249 | 3.17E-11 | 4.53E-09 |
| 79178 | 'THTPA' | -0.522852563 | 6.72E-04 | 0.009772498 |
| 7919 | 'DDX39B' | 0.199275463 | 0.002553559 | 0.027489163 |
| 79567 | 'RIPOR1' | -0.345071697 | 4.99E-04 | 0.007684623 |
| 79577 | 'CDC73' | -0.923631859 | 8.89E-17 | 2.85E-14 |
| 79583 | 'TMEM231' | 0.470690193 | 0.002105157 | 0.023729082 |
| 79596 | 'OBI1' | 0.494803631 | 0.003648565 | 0.036036204 |
| 79600 | 'TCTN1' | 0.488504559 | 3.31E-08 | 2.52E-06 |
| 79602 | 'ADIPOR2' | -0.741738409 | 8.05E-15 | 1.96E-12 |
| 79607 | 'FAM118B' | 0.469780294 | 8.18E-05 | 0.001851774 |
| 79618 | 'HMBOX1' | 0.326203112 | 9.71E-04 | 0.012981227 |
| 79630 | 'C1orf54' | -1.432785491 | 2.36E-09 | 2.32E-07 |
| 79633 | 'FAT4' | -0.95438962 | 9.82E-05 | 0.002157195 |
| 79654 | 'HECTD3' | 0.539599503 | 1.23E-06 | 5.42E-05 |
| 79680 | 'RTL10' | -0.440277901 | 0.002117309 | 0.023834444 |
| 79694 | 'MANEA' | -0.627666136 | 6.52E-04 | 0.009525992 |
| 79709 | 'COLGALT1' | 0.554971953 | 0.003153749 | 0.032274184 |
| 79719 | 'AAGAB' | -0.649791324 | 2.37E-09 | 2.32E-07 |
| 79750 | 'ZNF385D' | 1.133387152 | 0.003043247 | 0.031484546 |
| 79763 | 'ISOC2' | 0.504871332 | 0.003247833 | 0.032900281 |
| 79791 | 'FBXO31' | 0.394700846 | 4.82E-04 | 0.007458913 |
| 7980 | 'TFPI2' | 1.73599905 | 2.01E-04 | 0.003815716 |
| 79832 | 'QSER1' | -0.392931434 | 5.05E-05 | 0.00123851 |
| 79837 | 'PIP4K2C' | -0.599156819 | 0.001102868 | 0.014354178 |
| 79850 | 'TLCD3A' | -0.344370628 | 0.002497323 | 0.027106956 |
| 79853 | 'TM4SF20' | -1.93292404 | 5.27E-07 | 2.68E-05 |
| 79873 | 'NUDT18' | -0.639117903 | 0.003166345 | 0.032344671 |
| 79883 | 'PODNL1' | -1.231741539 | 1.90E-04 | 0.003683923 |
| 79888 | 'LPCAT1' | 0.502231982 | 5.53E-04 | 0.008368472 |
| 79892 | 'MCMBP' | -0.388607821 | 4.10E-05 | 0.00104824 |
| 79895 | 'ATP8B4' | -1.498110244 | 3.69E-05 | 9.64E-04 |
| 79896 | 'THNSL1' | 0.705772667 | 0.001959606 | 0.022425256 |
| 79921 | 'TCEAL4' | -0.66510298 | 0.001373882 | 0.016947204 |
| 79956 | 'ERMP1' | 1.105003585 | 3.72E-06 | 1.39E-04 |
| 79982 | 'DNAJB14' | -0.536499752 | 1.79E-05 | 5.25E-04 |
| 800 | 'CALD1' | -1.28849829 | 4.91E-08 | 3.49E-06 |
| 80000 | 'GREB1L' | 1.140031314 | 0.002333506 | 0.025773191 |
| 80014 | 'WWC2' | -0.636471209 | 4.91E-07 | 2.53E-05 |
| 80023 | 'NRSN2' | -0.304226413 | 0.003601118 | 0.035712833 |
| 80028 | 'FBXL18' | -0.727173765 | 5.24E-12 | 8.57E-10 |
| 80031 | 'SEMA6D' | -0.944647956 | 8.90E-04 | 0.012235143 |
| 80095 | 'ZNF606' | 0.828626269 | 3.04E-06 | 1.18E-04 |
| 80114 | 'BICC1' | 0.489011674 | 1.48E-04 | 0.0029899 |
| 80142 | 'PTGES2' | -0.440762327 | 0.002447768 | 0.026688366 |
| 80152 | 'CENPT' | 0.324667141 | 0.001068036 | 0.013964984 |
| 80162 | 'PGGHG' | -1.646193114 | 3.62E-14 | 8.54E-12 |
| 8021 | 'NUP214' | -0.449055914 | 4.06E-05 | 0.001041916 |
| 80212 | 'CCDC92' | 0.573504574 | 2.53E-06 | 1.02E-04 |
| 80213 | 'TM2D3' | 0.341697797 | 0.005296908 | 0.048263435 |
| 80221 | 'ACSF2' | -0.675960912 | 1.63E-05 | 4.91E-04 |
| 80222 | 'TARS2' | 0.509936566 | 2.48E-04 | 0.004486444 |
| 80223 | 'RAB11FIP1' | -1.068420001 | 6.66E-08 | 4.57E-06 |
| 80243 | 'PREX2' | -2.294898143 | 1.73E-04 | 0.003398892 |
| 80254 | 'CEP63' | -0.524617615 | 5.60E-04 | 0.008453745 |
| 80279 | 'CDK5RAP3' | 0.357238097 | 8.81E-04 | 0.01212104 |
| 80303 | 'EFHD1' | -1.7813118 | 0.001206834 | 0.015389166 |
| 80304 | 'WDCP' | -0.546054293 | 0.003177 | 0.032375685 |
| 80328 | 'ULBP2' | -1.25337393 | 5.18E-05 | 0.001262494 |
| 80347 | 'COASY' | 0.430505729 | 8.40E-06 | 2.81E-04 |
| 80351 | 'TNKS2' | -0.364045018 | 2.90E-05 | 7.88E-04 |
| 80381 | 'CD276' | -0.45245994 | 0.004320782 | 0.04108683 |
| 80758 | 'PRR7' | 0.659473381 | 0.00296879 | 0.030959202 |
| 8078 | 'USP5' | 0.327054419 | 8.88E-05 | 0.001988179 |
| 80821 | 'DDHD1' | 0.465518003 | 2.61E-04 | 0.004658213 |
| 80854 | 'SETD7' | 0.94192931 | 7.78E-12 | 1.20E-09 |
| 8091 | 'HMGA2' | 1.270756838 | 1.82E-06 | 7.59E-05 |
| 8099 | 'CDK2AP1' | 0.262509841 | 0.001185281 | 0.015148431 |
| 81 | 'ACTN4' | -0.610232009 | 1.82E-10 | 2.18E-08 |
| 8100 | 'IFT88' | 0.495355513 | 0.002135588 | 0.023987613 |
| 81035 | 'COLEC12' | 1.631032251 | 0.003474624 | 0.034721731 |
| 81037 | 'CLPTM1L' | 0.290440068 | 4.61E-04 | 0.007187379 |
| 811 | 'CALR' | 0.306818951 | 0.00258102 | 0.027682715 |
| 8125 | 'ANP32A' | -0.81474757 | 1.34E-17 | 4.93E-15 |
| 8148 | 'TAF15' | 0.307842584 | 7.13E-04 | 0.010213834 |
| 81533 | 'ITFG1' | 0.454276784 | 7.32E-09 | 6.44E-07 |
| 81537 | 'SGPP1' | -0.976479125 | 3.08E-13 | 6.08E-11 |
| 81552 | 'VOPP1' | 0.360518238 | 1.28E-04 | 0.002671039 |
| 81554 | 'RCC1L' | 0.341762766 | 0.002916659 | 0.030581997 |
| 81558 | 'FAM117A' | -0.779501807 | 0.005302898 | 0.048279943 |
| 81567 | 'TXNDC5' | -0.461535192 | 9.98E-06 | 3.26E-04 |
| 81573 | 'ANKRD13C' | -0.309329973 | 0.001222848 | 0.015558362 |
| 81577 | 'GFOD2' | 0.383510989 | 0.002677737 | 0.028483214 |
| 81605 | 'URM1' | -0.422992383 | 2.52E-04 | 0.004528782 |
| 81622 | 'UNC93B1' | -0.435366605 | 9.92E-04 | 0.013174879 |
| 81624 | 'DIAPH3' | -0.402092236 | 0.004222832 | 0.040303034 |
| 81631 | 'MAP1LC3B' | -0.40119537 | 3.14E-05 | 8.37E-04 |
| 81689 | 'ISCA1' | 0.588211052 | 1.59E-09 | 1.62E-07 |
| 81790 | 'RNF170' | -0.428491647 | 4.11E-04 | 0.006595941 |
| 81855 | 'SFXN3' | -0.715559686 | 3.38E-08 | 2.55E-06 |
| 8189 | 'SYMPK' | 0.453265585 | 9.38E-07 | 4.32E-05 |
| 81929 | 'SEH1L' | 0.596977592 | 4.20E-08 | 3.09E-06 |
| 8218 | 'CLTCL1' | -0.673287 | 0.004491524 | 0.042297461 |
| 825 | 'CAPN3' | 1.000089513 | 0.003185666 | 0.032444548 |
| 829 | 'CAPZA1' | -0.222173769 | 0.004488714 | 0.042294436 |
| 8301 | 'PICALM' | -0.414987245 | 0.001476725 | 0.017916793 |
| 8323 | 'FZD6' | -1.445215006 | 4.07E-62 | 6.92E-58 |
| 833 | 'CARS1' | 0.378364298 | 4.78E-04 | 0.007407717 |
| 83440 | 'ADPGK' | 0.501464906 | 0.00148375 | 0.017989151 |
| 83464 | 'APH1B' | 0.74984049 | 1.13E-08 | 9.43E-07 |
| 83604 | 'TMEM47' | -0.58032121 | 0.001261449 | 0.015918422 |
| 83606 | 'GUCD1' | 0.496832954 | 1.02E-05 | 3.32E-04 |
| 83716 | 'CRISPLD2' | -1.127977526 | 1.29E-04 | 0.002690043 |
| 83742 | 'MARVELD1' | -0.402158558 | 1.91E-05 | 5.58E-04 |
| 83892 | 'KCTD10' | -0.523705713 | 4.95E-05 | 0.001220224 |
| 83895 | 'KRTAP1-5' | -0.535075433 | 3.88E-06 | 1.44E-04 |
| 83930 | 'STARD3NL' | -1.197244764 | 2.34E-21 | 1.37E-18 |
| 8394 | 'PIP5K1A' | -0.482713244 | 1.71E-07 | 1.03E-05 |
| 8396 | 'PIP4K2B' | -0.424459303 | 0.001640631 | 0.019460887 |
| 83986 | 'FAM234A' | 0.296322364 | 0.005043969 | 0.046596403 |
| 83999 | 'KREMEN1' | -0.47599303 | 9.40E-04 | 0.01267032 |
| 840 | 'CASP7' | 0.842893522 | 0.001014967 | 0.013384333 |
| 84064 | 'HDHD2' | -0.950844051 | 6.15E-13 | 1.15E-10 |
| 84129 | 'ACAD11' | 0.448208829 | 2.18E-04 | 0.004074457 |
| 84131 | 'CEP78' | -0.764479239 | 9.52E-06 | 3.15E-04 |
| 84133 | 'ZNRF3' | -0.81095865 | 1.72E-04 | 0.00337286 |
| 84166 | 'NLRC5' | 0.474199966 | 1.39E-07 | 8.57E-06 |
| 8417 | 'STX7' | -0.357988707 | 3.92E-04 | 0.006378265 |
| 84172 | 'POLR1B' | 0.414178903 | 4.40E-04 | 0.006932224 |
| 84216 | 'TMEM117' | -0.957007066 | 0.002986053 | 0.031082014 |
| 84230 | 'LRRC8C' | -0.642624738 | 0.002916258 | 0.030581997 |
| 84232 | 'MAF1' | 0.378662232 | 1.50E-04 | 0.003011623 |
| 84233 | 'TMEM126A' | -0.510088889 | 5.43E-04 | 0.008247888 |
| 84236 | 'RHBDD1' | -0.51482147 | 4.61E-05 | 0.001150075 |
| 84243 | 'ZDHHC18' | 0.748126125 | 8.18E-07 | 3.84E-05 |
| 84251 | 'SGIP1' | 0.726580036 | 0.00341991 | 0.034275724 |
| 84255 | 'SLC37A3' | -0.636944053 | 1.30E-06 | 5.73E-05 |
| 84263 | 'HSDL2' | 0.484134968 | 0.005135293 | 0.047233016 |
| 84268 | 'RPAIN' | 0.485878696 | 5.51E-04 | 0.008351833 |
| 84287 | 'ZDHHC16' | 0.525636085 | 2.54E-04 | 0.004561839 |
| 84300 | 'UQCC2' | -0.440295763 | 9.94E-04 | 0.013179931 |
| 84301 | 'DDI2' | 0.70632991 | 1.53E-12 | 2.66E-10 |
| 84312 | 'BRMS1L' | -0.512773706 | 9.25E-04 | 0.012567284 |
| 84313 | 'VPS25' | 0.296651729 | 0.00251164 | 0.027200663 |
| 84343 | 'HPS3' | 0.473284535 | 9.31E-05 | 0.002066304 |
| 84439 | 'HHIPL1' | -0.446411708 | 0.001101789 | 0.014351114 |
| 84444 | 'DOT1L' | 0.358227633 | 0.004433987 | 0.041941523 |
| 84461 | 'NEURL4' | 0.473974469 | 2.87E-04 | 0.005026978 |
| 8450 | 'CUL4B' | 0.661320418 | 1.42E-04 | 0.002895017 |
| 84503 | 'ZNF527' | 0.660766178 | 0.002078027 | 0.023485578 |
| 84524 | 'ZC3H8' | 0.798553547 | 2.48E-04 | 0.004484195 |
| 84572 | 'GNPTG' | 0.377523869 | 5.69E-06 | 2.00E-04 |
| 8460 | 'TPST1' | -0.36730239 | 1.28E-04 | 0.002671039 |
| 84622 | 'ZNF594' | 0.553326809 | 0.004106758 | 0.039394284 |
| 84627 | 'ZNF469' | -1.115011046 | 1.55E-06 | 6.59E-05 |
| 84656 | 'GLYR1' | 0.376501778 | 3.04E-05 | 8.15E-04 |
| 84665 | 'MYPN' | 1.008892964 | 0.001432412 | 0.017529253 |
| 84669 | 'USP32' | 0.201650195 | 0.004475476 | 0.042193086 |
| 84674 | 'CARD6' | 0.428392848 | 6.73E-04 | 0.009776616 |
| 84679 | 'SLC9A7' | 0.594043805 | 0.00413562 | 0.039616207 |
| 84687 | 'PPP1R9B' | 0.383161266 | 2.48E-05 | 6.91E-04 |
| 84695 | 'LOXL3' | -0.86869032 | 1.83E-06 | 7.62E-05 |
| 8470 | 'SORBS2' | -0.575943275 | 9.97E-04 | 0.013206652 |
| 84709 | 'MGARP' | -1.088508087 | 0.001249892 | 0.015831347 |
| 84725 | 'PLEKHA8' | 0.498594432 | 1.07E-05 | 3.45E-04 |
| 84747 | 'UNC119B' | 0.283382177 | 0.005539625 | 0.049927115 |
| 84749 | 'USP30' | -0.695653032 | 1.08E-05 | 3.45E-04 |
| 84750 | 'FUT10' | -0.77121664 | 0.002668778 | 0.028423492 |
| 84804 | 'MFSD9' | -0.790930165 | 4.30E-04 | 0.006806694 |
| 84886 | 'C1orf198' | -1.072949523 | 1.42E-27 | 1.73E-24 |
| 84908 | 'FAM136A' | 0.34004717 | 0.001778508 | 0.020763107 |
| 8491 | 'MAP4K3' | -0.55415127 | 4.81E-10 | 5.24E-08 |
| 84912 | 'SLC35B4' | 0.386705605 | 0.001756074 | 0.020586036 |
| 84918 | 'LRP11' | -0.391881799 | 6.79E-04 | 0.009823253 |
| 84935 | 'MEDAG' | 1.169961252 | 5.97E-05 | 0.001427407 |
| 84936 | 'ZFYVE19' | -0.482743655 | 0.004304641 | 0.04099176 |
| 84947 | 'SERAC1' | -0.813063961 | 8.20E-05 | 0.001853652 |
| 8496 | 'PPFIBP1' | 0.805890605 | 7.37E-12 | 1.17E-09 |
| 84962 | 'AJUBA' | -0.442421953 | 1.48E-04 | 0.002983778 |
| 85012 | 'TCEAL3' | -0.627359878 | 6.57E-12 | 1.05E-09 |
| 8503 | 'PIK3R3' | -1.054513742 | 8.21E-11 | 1.06E-08 |
| 8516 | 'ITGA8' | -1.171772159 | 0.002475471 | 0.02694217 |
| 8518 | 'ELP1' | 0.38832707 | 5.16E-06 | 1.83E-04 |
| 8531 | 'YBX3' | 0.486675255 | 2.83E-05 | 7.73E-04 |
| 85364 | 'ZCCHC3' | 0.567515226 | 4.54E-04 | 0.007098591 |
| 85445 | 'CNTNAP4' | 3.691411471 | 1.49E-06 | 6.41E-05 |
| 8545 | 'CGGBP1' | -0.409918567 | 9.51E-07 | 4.36E-05 |
| 85453 | 'TSPYL5' | 0.624848612 | 3.42E-04 | 0.005691722 |
| 85460 | 'ZNF518B' | 0.475819714 | 0.001688402 | 0.019916345 |
| 85464 | 'SSH2' | 0.985696677 | 1.29E-07 | 8.03E-06 |
| 8560 | 'DEGS1' | -0.631603255 | 2.26E-14 | 5.41E-12 |
| 8565 | 'YARS1' | 0.446370888 | 0.003195562 | 0.032525848 |
| 8575 | 'PRKRA' | -0.396769404 | 2.08E-04 | 0.003930532 |
| 8611 | 'PLPP1' | -0.550192544 | 6.15E-04 | 0.009067817 |
| 8625 | 'RFXANK' | -0.391303815 | 6.77E-04 | 0.009804063 |
| 8630 | 'HSD17B6' | -2.85832244 | 3.31E-11 | 4.65E-09 |
| 8634 | 'RTCA' | -0.524258577 | 5.60E-07 | 2.83E-05 |
| 8641 | 'PCDHGB4' | 1.199179332 | 3.92E-17 | 1.33E-14 |
| 8667 | 'EIF3H' | -0.316935806 | 4.70E-05 | 0.001165585 |
| 8682 | 'PEA15' | -0.300145615 | 4.15E-04 | 0.006645783 |
| 87 | 'ACTN1' | -0.558532495 | 1.94E-06 | 8.05E-05 |
| 8703 | 'B4GALT3' | -0.525123414 | 0.003720056 | 0.036562859 |
| 8704 | 'B4GALT2' | 0.363622238 | 0.001441254 | 0.017593065 |
| 8714 | 'ABCC3' | 1.026426663 | 1.51E-22 | 1.03E-19 |
| 8720 | 'MBTPS1' | 0.442018771 | 5.58E-07 | 2.82E-05 |
| 8721 | 'EDF1' | -0.271824698 | 0.001886798 | 0.021817544 |
| 8723 | 'SNX4' | 0.440506433 | 2.76E-04 | 0.004884091 |
| 8724 | 'SNX3' | 0.209635846 | 0.001628877 | 0.019361995 |
| 8733 | 'GPAA1' | 0.35504453 | 4.77E-04 | 0.007400567 |
| 8751 | 'ADAM15' | 0.761627419 | 6.02E-14 | 1.35E-11 |
| 8760 | 'CDS2' | 0.670349601 | 1.17E-08 | 9.60E-07 |
| 8763 | 'CD164' | 0.342845873 | 0.002300457 | 0.025507608 |
| 8771 | 'TNFRSF6B' | 1.107815676 | 0.002983743 | 0.031076996 |
| 8775 | 'NAPA' | -0.338554865 | 1.30E-04 | 0.002709989 |
| 8793 | 'TNFRSF10D' | 0.530222788 | 0.002661438 | 0.028377454 |
| 8795 | 'TNFRSF10B' | 0.449340601 | 0.001885271 | 0.021814725 |
| 8801 | 'SUCLG2' | -0.627548861 | 1.66E-07 | 1.00E-05 |
| 8803 | 'SUCLA2' | -0.354771252 | 5.07E-04 | 0.007791717 |
| 8804 | 'CREG1' | 0.377638198 | 4.63E-07 | 2.42E-05 |
| 8818 | 'DPM2' | -0.431367974 | 2.84E-04 | 0.004996592 |
| 8824 | 'CES2' | -0.454542431 | 0.001334491 | 0.016630263 |
| 8825 | 'LIN7A' | -0.941307355 | 3.73E-04 | 0.006107916 |
| 8837 | 'CFLAR' | -0.440769253 | 3.22E-04 | 0.00543866 |
| 8841 | 'HDAC3' | -0.344271658 | 0.001266259 | 0.015962521 |
| 88455 | 'ANKRD13A' | -0.300629566 | 0.001385523 | 0.017066031 |
| 8853 | 'ASAP2' | -0.547419422 | 2.31E-04 | 0.004242302 |
| 8874 | 'ARHGEF7' | 0.439257515 | 3.36E-06 | 1.28E-04 |
| 8879 | 'SGPL1' | 0.530212729 | 3.42E-04 | 0.005691722 |
| 8880 | 'FUBP1' | -0.316525814 | 0.003366647 | 0.033881742 |
| 8898 | 'MTMR2' | 0.432023234 | 0.002390746 | 0.026254581 |
| 8907 | 'AP1M1' | 0.386014867 | 0.001043136 | 0.013681502 |
| 892 | 'CCNC' | -0.694899762 | 3.51E-07 | 1.91E-05 |
| 8933 | 'RTL8C' | 0.379921166 | 0.001767916 | 0.020684615 |
| 8935 | 'SKAP2' | -1.55465019 | 1.47E-12 | 2.58E-10 |
| 8939 | 'FUBP3' | -0.329750038 | 5.30E-05 | 0.001287702 |
| 8974 | 'P4HA2' | -0.818689498 | 6.02E-04 | 0.008942168 |
| 8975 | 'USP13' | -0.444949576 | 1.44E-04 | 0.00293674 |
| 89781 | 'HPS4' | 0.433282492 | 6.09E-04 | 0.008997122 |
| 89782 | 'LMLN' | 0.855040475 | 4.38E-10 | 4.86E-08 |
| 89795 | 'NAV3' | -0.554561292 | 1.47E-05 | 4.49E-04 |
| 89797 | 'NAV2' | 1.005533284 | 1.57E-19 | 7.40E-17 |
| 8985 | 'PLOD3' | 0.452920955 | 1.49E-07 | 9.08E-06 |
| 89853 | 'MVB12B' | -0.630934379 | 1.36E-04 | 0.002794113 |
| 89866 | 'SEC16B' | 1.216874501 | 0.004913133 | 0.045633683 |
| 89891 | 'DYNC2I2' | 0.373686949 | 0.004321876 | 0.04108683 |
| 8991 | 'SELENBP1' | -0.670142067 | 0.002656898 | 0.028358246 |
| 900 | 'CCNG1' | -0.435983251 | 7.87E-05 | 0.001788291 |
| 90007 | 'MIDN' | 0.562564967 | 1.17E-06 | 5.21E-05 |
| 901 | 'CCNG2' | -0.654485176 | 1.17E-06 | 5.21E-05 |
| 90102 | 'PHLDB2' | -0.541080121 | 9.51E-04 | 0.012787589 |
| 9013 | 'TAF1C' | 0.407943958 | 8.08E-04 | 0.011327475 |
| 90135 | 'BTBD6' | 0.310149083 | 0.003660943 | 0.036116487 |
| 9019 | 'MPZL1' | -0.392873494 | 5.16E-07 | 2.64E-05 |
| 90317 | 'ZNF616' | 0.976844285 | 7.02E-07 | 3.41E-05 |
| 90333 | 'ZNF468' | 0.485395734 | 0.001715064 | 0.020160893 |
| 90338 | 'ZNF160' | 0.445991631 | 1.76E-04 | 0.0034516 |
| 90355 | 'MACIR' | 1.102457035 | 4.48E-06 | 1.62E-04 |
| 9039 | 'UBA3' | -0.632271897 | 5.49E-14 | 1.24E-11 |
| 90416 | 'CCDC32' | 0.766788098 | 7.20E-04 | 0.010300293 |
| 9044 | 'BTAF1' | 0.567524501 | 1.46E-09 | 1.51E-07 |
| 90507 | 'SCRN2' | -1.047684465 | 4.09E-07 | 2.18E-05 |
| 90550 | 'MCU' | -0.446116676 | 0.004555233 | 0.042778919 |
| 90557 | 'CCDC74A' | 0.725660841 | 7.53E-04 | 0.010698538 |
| 9057 | 'SLC7A6' | 0.350859347 | 4.85E-04 | 0.007498762 |
| 9070 | 'ASH2L' | -0.40417366 | 2.40E-04 | 0.004370124 |
| 90861 | 'JPT2' | 0.584175069 | 1.86E-07 | 1.10E-05 |
| 90993 | 'CREB3L1' | -0.558209164 | 0.003359511 | 0.03385001 |
| 91 | 'ACVR1B' | 0.329996085 | 0.001644203 | 0.019489648 |
| 91039 | 'DPP9' | 0.468636384 | 6.05E-07 | 3.02E-05 |
| 91151 | 'TIGD7' | 0.764285733 | 3.61E-04 | 0.005956788 |
| 9117 | 'SEC22C' | -0.393434204 | 0.003591058 | 0.035675514 |
| 9120 | 'SLC16A6' | 1.838340492 | 1.30E-05 | 4.04E-04 |
| 9128 | 'PRPF4' | 0.340522832 | 0.003739485 | 0.036699636 |
| 91300 | 'R3HDM4' | -0.513223199 | 3.05E-05 | 8.15E-04 |
| 91526 | 'ANKRD44' | -0.731649133 | 0.001632637 | 0.019393131 |
| 91584 | 'PLXNA4' | -0.83418907 | 2.88E-04 | 0.005047225 |
| 91608 | 'RASL10B' | -0.676602912 | 5.90E-04 | 0.008811501 |
| 91663 | 'MYADM' | -0.629491578 | 4.05E-04 | 0.006533313 |
| 9167 | 'COX7A2L' | -0.608223604 | 2.60E-07 | 1.49E-05 |
| 91754 | 'NEK9' | 0.465592115 | 3.23E-05 | 8.58E-04 |
| 9183 | 'ZW10' | 0.64530196 | 1.99E-05 | 5.76E-04 |
| 9189 | 'ZBED1' | -0.839544393 | 1.44E-07 | 8.83E-06 |
| 9197 | 'SLC33A1' | 0.50620469 | 3.01E-04 | 0.005193706 |
| 92 | 'ACVR2A' | -0.629125702 | 0.003872424 | 0.037699576 |
| 9200 | 'HACD1' | -0.739120921 | 0.003103767 | 0.031877839 |
| 9202 | 'ZMYM4' | -0.34424636 | 4.27E-06 | 1.56E-04 |
| 9208 | 'LRRFIP1' | -0.328175786 | 3.18E-04 | 0.005387313 |
| 9213 | 'XPR1' | -1.009777964 | 2.47E-18 | 1.02E-15 |
| 9221 | 'NOLC1' | 0.825179356 | 3.53E-10 | 4.08E-08 |
| 92285 | 'ZNF585B' | 0.96845919 | 6.02E-08 | 4.16E-06 |
| 92305 | 'TMEM129' | -0.416233599 | 3.88E-04 | 0.006320962 |
| 9236 | 'CCPG1' | 0.432063602 | 3.71E-04 | 0.00608107 |
| 9240 | 'PNMA1' | 0.459843013 | 1.66E-04 | 0.003281126 |
| 9260 | 'PDLIM7' | -0.722732518 | 2.27E-05 | 6.42E-04 |
| 9265 | 'CYTH3' | -0.794928368 | 1.80E-04 | 0.003515318 |
| 92703 | 'TMEM183A' | 0.250665868 | 0.003404769 | 0.03418444 |
| 92715 | 'DPH7' | 0.524305982 | 0.003084229 | 0.031753919 |
| 92745 | 'SLC38A5' | 0.910663464 | 2.82E-04 | 0.004958778 |
| 9275 | 'BCL7B' | 0.445750306 | 0.005191284 | 0.047595172 |
| 92822 | 'ZNF276' | 0.592829396 | 3.45E-04 | 0.005727873 |
| 93109 | 'TMEM44' | 0.396947198 | 0.005311012 | 0.048290175 |
| 9315 | 'NREP' | -0.978707319 | 0.001457691 | 0.017723771 |
| 93210 | 'PGAP3' | -0.468254185 | 0.001081321 | 0.014106139 |
| 9330 | 'GTF3C3' | -0.606524294 | 9.59E-07 | 4.38E-05 |
| 9331 | 'B4GALT6' | -0.886286836 | 1.67E-05 | 4.99E-04 |
| 9334 | 'B4GALT5' | -0.46546072 | 1.42E-04 | 0.002903313 |
| 9338 | 'TCEAL1' | -0.405400317 | 0.002640378 | 0.0282449 |
| 93380 | 'MMGT1' | -0.341951973 | 0.001617909 | 0.019245075 |
| 93487 | 'MAPK1IP1L' | -0.306016497 | 2.72E-04 | 0.004823898 |
| 9354 | 'UBE4A' | -0.38710458 | 9.00E-04 | 0.012322825 |
| 9361 | 'LONP1' | 0.37291282 | 0.002893655 | 0.030437092 |
| 93611 | 'FBXO44' | -0.592604497 | 2.63E-04 | 0.004693196 |
| 93624 | 'TADA2B' | 0.384270641 | 0.005296405 | 0.048263435 |
| 9380 | 'GRHPR' | -0.347538182 | 0.004452217 | 0.042029483 |
| 9382 | 'COG1' | 0.419477467 | 0.003222406 | 0.032720705 |
| 9394 | 'HS6ST1' | -0.893121978 | 0.002087032 | 0.02354039 |
| 9411 | 'ARHGAP29' | 0.492389438 | 1.87E-05 | 5.46E-04 |
| 9415 | 'FADS2' | -1.173810711 | 2.10E-26 | 2.10E-23 |
| 9416 | 'DDX23' | 0.387200068 | 3.21E-04 | 0.005436005 |
| 9424 | 'KCNK6' | -0.84706619 | 4.20E-18 | 1.66E-15 |
| 94274 | 'PPP1R14A' | -1.454382292 | 9.46E-09 | 8.08E-07 |
| 9442 | 'MED27' | -0.417756255 | 1.26E-04 | 0.002639008 |
| 9448 | 'MAP4K4' | 0.712596153 | 1.42E-19 | 7.09E-17 |
| 9459 | 'ARHGEF6' | -0.744951529 | 4.24E-05 | 0.001072251 |
| 9475 | 'ROCK2' | -0.484457508 | 1.95E-04 | 0.003742419 |
| 9477 | 'MED20' | -0.7842218 | 2.41E-05 | 6.74E-04 |
| 948 | 'CD36' | 1.001173259 | 0.002931398 | 0.030663325 |
| 9495 | 'AKAP5' | -1.612908026 | 0.001079064 | 0.014087508 |
| 9497 | 'SLC4A7' | 0.794778097 | 9.41E-14 | 2.00E-11 |
| 9500 | 'MAGED1' | -0.344481068 | 1.01E-06 | 4.59E-05 |
| 9516 | 'LITAF' | -0.742301221 | 6.15E-05 | 0.001461337 |
| 9525 | 'VPS4B' | -0.749301547 | 2.38E-10 | 2.81E-08 |
| 9537 | 'TP53I11' | -0.669786695 | 0.001696153 | 0.019980047 |
| 955 | 'ENTPD6' | 0.721066503 | 2.81E-11 | 4.05E-09 |
| 956 | 'ENTPD3' | -0.816514764 | 0.00325649 | 0.032907047 |
| 9562 | 'MINPP1' | 0.479500429 | 0.001168091 | 0.014973764 |
| 9563 | 'H6PD' | 0.403395509 | 0.003754001 | 0.0367596 |
| 9572 | 'NR1D1' | 0.773597752 | 4.64E-04 | 0.007219005 |
| 960 | 'CD44' | 0.812457196 | 0.00102154 | 0.013439731 |
| 9612 | 'NCOR2' | -0.589875709 | 1.91E-04 | 0.003683923 |
| 9636 | 'ISG15' | -1.215071375 | 3.14E-04 | 0.005354657 |
| 9637 | 'FEZ2' | -0.367864865 | 0.003742265 | 0.036705723 |
| 9640 | 'ZNF592' | 0.350489775 | 5.35E-04 | 0.008151227 |
| 9643 | 'MORF4L2' | -0.590551107 | 5.17E-10 | 5.59E-08 |
| 9644 | 'SH3PXD2A' | -1.060409086 | 2.13E-04 | 0.003992426 |
| 9646 | 'CTR9' | -0.388816016 | 1.06E-04 | 0.00228199 |
| 966 | 'CD59' | 0.558329924 | 2.22E-07 | 1.28E-05 |
| 9668 | 'ZNF432' | 0.890625184 | 1.28E-12 | 2.28E-10 |
| 967 | 'CD63' | 0.308211281 | 0.00531221 | 0.048290175 |
| 9672 | 'SDC3' | -0.426587932 | 0.002718105 | 0.028858435 |
| 968 | 'CD68' | 1.044763006 | 4.93E-14 | 1.13E-11 |
| 9686 | 'VGLL4' | 0.500505159 | 1.98E-06 | 8.18E-05 |
| 9689 | 'BZW1' | 0.26379444 | 0.001139698 | 0.014642921 |
| 9697 | 'TRAM2' | -0.422943185 | 1.75E-04 | 0.00342838 |
| 9711 | 'RUBCN' | 0.55775332 | 3.69E-05 | 9.64E-04 |
| 9728 | 'SECISBP2L' | -0.374009002 | 2.33E-04 | 0.004273708 |
| 9739 | 'SETD1A' | 0.491772054 | 4.70E-05 | 0.001165585 |
| 9747 | 'TCAF1' | -0.483344775 | 5.49E-06 | 1.94E-04 |
| 9748 | 'SLK' | 0.463889478 | 4.61E-04 | 0.007187379 |
| 9749 | 'PHACTR2' | 0.50650872 | 6.02E-05 | 0.001436991 |
| 975 | 'CD81' | 0.182850333 | 0.001442174 | 0.017593065 |
| 9770 | 'RASSF2' | -2.077761893 | 1.51E-05 | 4.60E-04 |
| 9771 | 'RAPGEF5' | -1.496326686 | 1.00E-10 | 1.27E-08 |
| 9779 | 'TBC1D5' | -0.420342841 | 0.003633148 | 0.0359048 |
| 9780 | 'PIEZO1' | -0.495397275 | 5.87E-04 | 0.008783348 |
| 9790 | 'BMS1' | 0.323303283 | 0.001920989 | 0.022092676 |
| 9802 | 'DAZAP2' | -0.411981083 | 2.10E-06 | 8.54E-05 |
| 9804 | 'TOMM20' | -0.282893731 | 2.10E-04 | 0.003944938 |
| 9812 | 'DELE1' | -0.538260125 | 2.04E-05 | 5.87E-04 |
| 9814 | 'SFI1' | 0.484550008 | 0.00398403 | 0.038481949 |
| 9821 | 'RB1CC1' | 0.297051185 | 0.00199985 | 0.0228144 |
| 9828 | 'ARHGEF17' | -0.502447748 | 3.45E-04 | 0.005727873 |
| 9836 | 'LCMT2' | -0.51343026 | 0.002227729 | 0.024782026 |
| 9842 | 'PLEKHM1' | 0.311647621 | 3.77E-04 | 0.00614434 |
| 9843 | 'HEPH' | -0.558040923 | 8.16E-05 | 0.001848589 |
| 9848 | 'MFAP3L' | -1.011944266 | 8.47E-05 | 0.001907504 |
| 9852 | 'EPM2AIP1' | 0.302291759 | 0.00145751 | 0.017723771 |
| 9860 | 'LRIG2' | 0.372799237 | 0.004440506 | 0.041956483 |
| 9865 | 'TRIL' | -3.236767112 | 0.00140683 | 0.017303404 |
| 9887 | 'SMG7' | 0.284397013 | 0.002657647 | 0.028358246 |
| 9889 | 'ZBED4' | 0.493805434 | 4.24E-04 | 0.006747964 |
| 9891 | 'NUAK1' | -0.837781946 | 6.05E-05 | 0.001441397 |
| 9895 | 'TECPR2' | 0.405329779 | 4.25E-04 | 0.006748591 |
| 9897 | 'WASHC5' | -0.42282022 | 1.29E-05 | 4.01E-04 |
| 9908 | 'G3BP2' | 0.852475322 | 1.52E-31 | 2.36E-28 |
| 9919 | 'SEC16A' | 0.313878861 | 2.02E-04 | 0.00383495 |
| 9922 | 'IQSEC1' | -0.466060433 | 0.001392524 | 0.017139838 |
| 9937 | 'DCLRE1A' | -0.777067494 | 4.44E-04 | 0.006982539 |
| 994 | 'CDC25B' | 0.591032016 | 5.12E-09 | 4.60E-07 |
| 9956 | 'HS3ST2' | 1.429603359 | 0.003008524 | 0.031210951 |
| 9960 | 'USP3' | 0.335793904 | 0.003254475 | 0.032907047 |
| 9962 | 'SLC23A2' | -1.276974103 | 6.37E-29 | 9.03E-26 |
| 9976 | 'CLEC2B' | 0.874504966 | 1.09E-04 | 0.002333438 |
| 998 | 'CDC42' | -0.26401625 | 2.49E-04 | 0.004486444 |
| 9990 | 'SLC12A6' | 0.41373124 | 0.001384761 | 0.017066031 |
| 9993 | 'DGCR2' | -0.455279942 | 6.55E-05 | 0.001547352 |
